# Cardiac Molecular Remodeling by Anticancer Drugs: Doxorubicin Affects More Metabolism While Mitoxantrone Impacts More Autophagy in Adult CD-1 Male Mice

**DOI:** 10.3390/biom13060921

**Published:** 2023-05-31

**Authors:** Sofia Reis Brandão, Ana Reis-Mendes, Margarida Duarte-Araújo, Maria João Neuparth, Hugo Rocha, Félix Carvalho, Rita Ferreira, Vera Marisa Costa

**Affiliations:** 1 Associate Laboratory i4HB—Institute for Health and Bioeconomy, Faculty of Pharmacy, University of Porto, 4050-313 Porto, Portugal; afreis.mendes@gmail.com (A.R.-M.); felixdc@ff.up.pt (F.C.); 2 Laboratory of Toxicology, UCIBIO-Applied Molecular Biosciences Unit, REQUIMTE, Department of Biological Sciences, Faculty of Pharmacy, University of Porto, 4050-313 Porto, Portugal; 3 LAQV-REQUIMTE, Department of Chemistry, University of Aveiro, 3810-193 Aveiro, Portugal; ritaferreira@ua.pt; 4 LAQV-REQUIMTE, Faculty of Pharmacy, University of Porto, 4050-313 Porto, Portugal; mdcma@icbas.up.pt; 5 Department of Imuno-Physiology and Pharmacology, Institute of Biomedical Sciences Abel Salazar, University of Porto, 4050-313 Porto, Portugal; 6 Laboratory for Integrative and Translational Research in Population Health (ITR), Research Centre in Physical Activity, Health and Leisure (CIAFEL), Faculty of Sports, University of Porto, 4200-450 Porto, Portugal; mjoao.neuparth@ipsn.cespu.pt; 7 TOXRUN—Toxicology Research Unit, University Institute of Health Sciences, CESPU, 4585-116 Gandra, Portugal; 8 Newborn Screening, Metabolism and Genetics Unit, Human Genetics Department, National Institute of Health Doutor Ricardo Jorge, 4000-053 Porto, Portugal; hugo.rocha@insa.min-saude.pt; 9 Department of Pathological, Cytological and Thanatological Anatomy, School of Health, Polytechnic Institute of Porto, 4200-072 Porto, Portugal

**Keywords:** cardiotoxicity, anticancer agents, molecular mechanisms, mitochondrial dynamics

## Abstract

Doxorubicin (DOX) and mitoxantrone (MTX) are classical chemotherapeutic agents used in cancer that induce similar clinical cardiotoxic effects, although it is not clear if they share similar underlying molecular mechanisms. We aimed to assess the effects of DOX and MTX on the cardiac remodeling, focusing mainly on metabolism and autophagy. Adult male CD-1 mice received pharmacologically relevant cumulative doses of DOX (18 mg/kg) and MTX (6 mg/kg). Both DOX and MTX disturbed cardiac metabolism, decreasing glycolysis, and increasing the dependency on fatty acids (FA) oxidation, namely, through decreased AMP-activated protein kinase (AMPK) and glyceraldehyde-3-phosphate dehydrogenase (GAPDH) content and decreased free carnitine (C0) and increased acetylcarnitine (C2) concentration. Additionally, DOX heavily influenced glycolysis, oxidative metabolism, and amino acids turnover by exclusively decreasing phosphofructokinase (PFKM) and electron transfer flavoprotein-ubiquinone oxidoreductase (ETFDH) content, and the concentration of several amino acids. Conversely, both drugs downregulated autophagy given by the decreased content of autophagy protein 5 (ATG5) and microtubule-associated protein light chain 3 (LC3B), with MTX having also an impact on Beclin1. These results emphasize that DOX and MTX modulate cardiac remodeling differently, despite their clinical similarities, which is of paramount importance for future treatments.

## 1. Introduction

Doxorubicin (DOX) and mitoxantrone (MTX) belong to the class of anthracyclines and anthracenediones, respectively, being classical chemotherapeutic agents. These drugs have been in clinical practice for several decades and have been widely used to successfully treat numerous solid cancers, leukemias, and lymphoma. Moreover, MTX was also approved for the treatment of multiple sclerosis. The most described anticancer mechanism of action for both DOX and MTX is the inhibition of topoisomerase II (TOP2). Nonetheless, other mechanisms may be relevant [1,2,3].

Conversely, these anticancer agents are not selective, and they affect non-target organs, resulting in severe adverse side effects, such as fatigue, alopecia, myelosuppression, mucositis, and cardiotoxicity [3]. The cardiotoxicity of anthracyclines has been known since the 1970s. The synthesis of MTX was expected to minimize the cardiotoxic effects of DOX while keeping its anticancer efficacy, which unfortunately was not the case. Taking into consideration the isotoxic/pharmacokinetic dose conversion between them, it has become accepted that one molecule of MTX sensibly exhibits the similar anticancer and cardiotoxic effects of three to four molecules of DOX [4,5,6]. Clinical studies show that both DOX and MTX have similar clinical cardiotoxic manifestations, including arrhythmias, electrocardiographic changes, myopericarditis, and left ventricular dysfunction (LVD), which may progress to heart failure [6,7,8]. Indeed, previous studies performed in mice have shown that DOX treatment led to decreased cardiac output, heart rate, stroke volume, and blood pressure, as well as systolic and diastolic left ventricle function and decreased ejection fraction [9,10,11]. For MTX, echocardiographic changes have been reported on patients treated for multiple sclerosis and acute myeloid leukemia, including reduced ejection fraction along with diastolic and systolic dysfunction as the major findings [12,13]. Thus, the patients requiring chemotherapy with these agents may have their life span and quality of life affected, as cardiotoxicity is an important and clinically limiting side effect of their use. Cardiotoxicity is, nowadays, the second cause of death in cancer patients [3,14]. Taking the notion that patients’ health may be extremely compromised, lifelong cumulative maximum recommended doses for these drugs were established for humans, namely 400–550 mg/m^2^ for DOX and 100–140 mg/m^2^ for MTX [15].

Classically, DOX-induced cardiotoxicity has been attributed to reactive oxygen species (ROS) formation due to its ability to enter redox cycling, leading to oxidative stress, lipid peroxidation, protein modification, DNA damage, cardiomyocyte apoptosis, mitochondrial damage, and disturbance of calcium and iron homeostasis. At present, new adverse outcome pathways (AOPs) have been described referring to the modulation of signaling pathways dependent or independent of cardiac TOP2 β inhibition to explain the cardiotoxicity of anthracyclines [15,16,17,18,19,20,21,22,23]. On the other hand, although early on the discovery of the mechanisms for cardiotoxicity of MTX they were considered similar to those of DOX, nowadays, it is established that they are dissimilar. MTX AOPs are far less studied when compared to DOX AOPs, but it seems that MTX leads to impaired cardiac ATP and iron homeostasis, and affects mitochondria metabolism [15,18,24,25,26].

Under healthy conditions, the heart muscle obtains approximately 60% of energy from fatty acids (FA), 35% from carbohydrates, and 5% from ketone bodies and amino acids. Among these energy sources, FA are the most important in the cardiac muscle since their oxidation allows higher levels of ATP, produced mainly in the mitochondria, which is essential to keep cardiac function and homeostasis [27,28]. Other signaling pathways, including autophagy, may be activated during metabolic stress, such as nutrient deprivation. Indeed, autophagy represents an adaptive process that allows cell survival in stressful conditions. Moreover, the specific elimination of damaged mitochondria through autophagy (mitophagy) allows for reducing mitochondria-mediated apoptosis and necrosis in the myocardium [29]. Considering the massive number of mitochondria inside the heart muscle, the regulation of mitochondrial homeostasis is essential to keep the normal cardiac function [28,30]. Mitochondria are known to be targets for both DOX and MTX, so it becomes pivotal to study them in the context of cardio-oncology. Indeed, dysregulation of these processes has been associated with heart failure in both patients and animal models, like what is seen for the cardiotoxicity of anticancer drugs [18,29,31].

Therefore, this study aimed to assess the effects of DOX and MTX on cardiac molecular mechanisms focusing on the heart’s metabolism, autophagy, and mitochondrial biogenesis, all key regulators of cardiac homeostasis. To this end, we used adult male CD-1 mice, which received multiple administrations of these drugs to mimic the human therapy, with maximum cumulative doses of 18 mg/kg and 6 mg/kg for DOX and MTX, respectively. These cumulative doses are clinically equivalent and pharmacologically relevant [4,5].

## 2. Materials and Methods

### 2.1. Chemicals

Generally, all reagents were of the highest grade available. Rabbit anti-microtubule-associated protein light chain 3 (LC3B, L7543), horseradish peroxidase-conjugated anti-goat (A5420) antibodies, acetyl coenzyme A (acetyl-CoA) sodium salt, bovine serum albumin (BSA), 5,5′-dithiobis-(2-nitrobenzoic acid) (DTNB), DOX hydrochloride, MTX dihydrochloride, phenylmethanesulfonyl fluoride (PMSF), phosphate-buffered saline (PBS), Ponceau S, protease inhibitors cocktail, sodium chloride (NaCl), Triton X-100, and Tween20 were obtained from Sigma-Aldrich (St. Louis, MO, USA). Deuterated acylcarnitine internal standard solutions were obtained from Isotope labs (Cambridge, MA, USA). Horseradish peroxidase-conjugated anti-mouse (NA931) or anti-rabbit (NA934) antibodies were obtained from GE Healthcare (Buckinghamshire, UK). Enhanced chemiluminescence (ECL) reagent was obtained from Bio-Rad (Hercules, CA, USA).

Goat polyclonal anti-sirtuin 3 (SIRT3, sc-49744), mouse monoclonal anti-Beclin1 (sc-48341), anti-B-cell lymphoma-2 (BCL2, sc-7382), anti-glycogen synthase kinase 3 β (GSK-3β, sc-377213), anti-mitofusin1 (Mfn1, sc-166644), anti-Parkin (sc-32282), rabbit polyclonal anti-heat shock protein 27 kDa (HSP27, sc-9012) and anti-mitochondrial transcription factor A (Tfam, sc-28200) antibodies were obtained from Santa Cruz Technologies (Dallas, Texas, USA). Mouse anti-heat shock protein 70 kDa (HSP70, SPA-810) antibody was obtained from Stress gen (Farmingdale, NY, USA).

Mouse monoclonal anti-AMP-activated protein kinase (AMPK, ab80039), anti-ATP synthase subunit β (ATPB, ab14730), anti-BCL2 interacting protein 3 (BNIP3, ab10433), anti-glucose transporter GLUT4 (GLUT4, ab48547), anti-peroxisome proliferator-activated receptor γ (PPARγ, ab41928), rabbit monoclonal anti-autophagy protein 5 (ATG5, ab108327), anti-BCL2 associated X-protein (BAX, ab32503), anti-phosphofructokinase (PFKM, ab154804), rabbit polyclonal anti-AMPK phosphorylated (pAMPK, ab23875), anti-electron transfer flavoprotein-ubiquinone oxidoreductase (ETFDH, ab91508), anti-glyceraldehyde-3-phosphate dehydrogenase (GAPDH, ab9485), anti-peroxisome proliferator-activated receptor α (PPARα, ab24509), and anti-peroxisome proliferator-activated receptor γ coactivator 1 α (PGC-1α, ab191838) antibodies were obtained from Abcam (Cambridge, UK).

### 2.2. Animals

Twenty-seven adult CD-1 male mice (*Mus musculus*) were obtained from Charles River Laboratories (L’Arbresle, France) and maintained in the Institute of Biomedical Sciences Abel Salazar (ICBAS-UP) rodent animal house facility, following previously described procedures and conditions [32]. The experimental protocol was performed in accordance with the European Council Directive (2010/63/EU). The project was approved by the local animal welfare body (ORBEA, project nº 140/2015) and by the national competent authorities—General Directorate of Food and Veterinary (DGAV, reference nº 0421/000/000/2016).

### 2.3. Experimental Design

Mice were divided into the following three groups: control (CTRL, *n* = 9), DOX (*n* = 9), and MTX (*n* = 9). All animals received six intraperitoneal (i.p.) injections of either saline solution (0.9% NaCl, CTRL group), DOX (cumulative dose of 18 mg/kg of DOX), or MTX (cumulative dose of 6 mg/kg of MTX) solutions prepared in 0.9% NaCl in sterile conditions, distributed twice a week for three weeks. The CTRL group received 0.9% NaCl i.p. injections in the same volume and conditions as the drug-exposed groups.

The injection schedule was selected to mimic human cancer therapy consisting of multiple administrations at separated time-points [3,33]. The cumulative doses equivalence to humans was achieved using the allometric scaling and the conversion factor 37 for body area surface between mice and humans, as recommended by the US Food and Drug Administration [34]. The 18 mg/kg of DOX and the 6 mg/kg of MTX in adult mice corresponded roughly to 100 mg/m^2^ or 30 mg/m^2^ in humans, respectively, and were much lower than the maximum lifetime doses recommended for patients [15]. One week after the last i.p. injection, mice were deeply anesthetized with isoflurane and sacrificed by exsanguination.

### 2.4. Blood Collection and Serum Analysis

After reaching deep anesthesia, the abdominal cavity was opened to expose the inferior vena cava. Blood was collected in tubes containing an inert clotting agent (Vetlab ZT Plain + Gel, 1.1 mL fill, Vetlab Supplies, West Sussex, UK) and was allowed to clot at room temperature for at least 45 min. The clot was pelleted by centrifugation (900*g* ×, 10 min, room temperature), and the serum obtained was aliquoted and stored at −80 °C until the analysis. An aliquot was used to determine serum total protein, albumin, and glucose levels, as well as alanine aminotransferase (ALAT), aspartate aminotransferase (ASAT), and creatine kinase-MB (CK-MB) activity using an AutoAnalyzer (Prestige 24i, Cormay PZ, Diamond Diagnostics, Holliston, MA, USA).

### 2.5. Heart Collection and Homogenization

After blood collection, hearts were rapidly excised, carefully dried, and weighed. A slice comprising the two cardiac ventricles was homogenized in lysis buffer [100 mM potassium phosphate, pH 7.4, 0.1% (*v*/*v*) Triton X-100, with protease inhibitors cocktail (1:400) and PMSF (1:1000); 50 mg of tissue/mL of buffer] using a Teflon pestle on a tight-fitting Potter-Elvehjem glass homogenizer at 0–4 °C. The cardiac homogenates were aliquoted and stored at −80 °C for further biochemical analysis. The protein content of the homogenate was assessed with the commercial method DC Protein Assay (Bio-Rad, Hercules, CA, USA), following the manufacturer’s recommendations and using BSA as standard.

### 2.6. Histological Analysis

Histological assessment of the heart was performed as previously reported [35,36]. Hematoxylin and eosin staining was performed on heart sections to assess tissue damage. The images were taken with a Carl Zeiss Imager A1 light microscope equipped with an AxioCam MRc 5 digital camera (Oberkochen, Germany).

### 2.7. Western Blotting Analysis

The amount of protein (30 μg) from each group was loaded on a 12.5% SDS-PAGE according to Laemmli [37] and then blotted onto a nitrocellulose membrane (Amersham Protran, GE Healthcare, Germany) for 1 h at 200 mA in transfer buffer. Membranes were blocked with 5% (*w*/*v*) non-fat dry milk in Tris-buffered saline with Tween 20 (TBS-T) and then incubated overnight at 4 °C with the primary antibody diluted in 5% (*w*/*v*) non-fat dry milk in TBS-T, pending on the antibody used (Supplementary Table S1). The proteins searched were AMPK, ATG5, ATPB, BAX, BCL2, Beclin1, BNIP3, ETFDH, GAPDH, GLUT4, GSK-3β, HSP27, HSP70, LC3B, Mfn1, pAMPK, Parkin, PFKM, PGC-1α, PPARα, PPARγ, SIRT3, and Tfam. Membranes were washed with TBS-T and then incubated with the appropriate secondary horseradish peroxidase-conjugated antibody diluted 1:1000 or 1:2000 in 5% (*w*/*v*) non-fat dry milk in TBS-T. Membranes were exposed with ECL reagent according to the manufacturer’s instructions. The immunoreactive bands were detected using the ChemiDoc XRS+ Imaging System (Bio-Rad, Hercules, CA, USA), and the images obtained were analyzed with Image Lab software version 6.0.1 (Bio-Rad, Hercules, CA, USA) to retrieve the optical density of each sample in each membrane. Then, the band intensity values obtained in each membrane were normalized to total protein using the Ponceau S staining and uniformized using the CTRL samples as reference, being expressed as relative content. One representative blot is presented in the figure results, but all the blots obtained are indicated in Supplementary Figure S2. The protein normalization was performed using Ponceau S staining since most housekeeping markers are affected in the cardiac hypertrophy/dysfunction caused by anticancer drugs [38,39,40,41,42]. The Ponceau S staining of each membrane is presented in Supplementary Figure S2.

### 2.8. Acylcarnitines and Amino Acids Analysis

Quantification of acylcarnitines and amino acids was conducted through tandem mass spectrometry (MS/MS), according to Petucci et al. [43]. Cardiac homogenates (100 μL) were vortexed and centrifuged at 20,000 *g* (10 °C, 5 min). The supernatant (100 μL) was distributed into a 96-well plate and mixed with 100 μL of methanol containing deuterated acylcarnitine internal standard solutions. The mixture was dried under a nitrogen flow and shortly thereafter derivatized to the corresponding methyl esters by incubation with 60 μL of 3N methanolic HCl (70 °C, 15 min). Finally, samples were dried using nitrogen flow and reconstituted with 200 μL of 80% acetonitrile (0.01% formic acid) for flow injection MS/MS in an API 4000 QTRAP (Sciex, Washington, DC, USA).

### 2.9. Determination of Citrate Synthase (CS) Activity

CS activity was measured according to Coore *et al.* [44]. In brief, the acetyl-CoA connects with oxaloacetate, producing citrate and coenzyme A (CoA). The free thiol groups of CoA react with DTNB, generating 2-nitro-5-thiolbenzoate anion that has a strong absorption at 412 nm (molar extinction coefficient of 13.6 mM^−1^ ⋅ cm^−1^). The absorbance was read for approximately 2 min at 30 °C in a microplate reader (Multiskan GO, Thermo Fischer Scientific, Northumberland, UK). The values obtained were normalized to total protein.

### 2.10. Statistical Analysis

Data are presented as mean ± standard deviation (SD). Statistical analysis was performed with GraphPad Prism software (version 6.0.1, GraphPad software Inc, La Jolla, CA, USA), and the experimental groups were compared using ordinary one-way ANOVA followed by Tukey’s multiple comparisons test (if otherwise, it is mentioned in the figure legend). Results were considered different when *p*-value<0.05. For *p*-value < 0.1 and > 0.05, a tendency was assumed.

## 3. Results

### 3.1. DOX Had a Greater Impact on Whole-Body Weight and Serum Biomarkers of Heart Damage Than MTX

All groups started with nine animals; however, four mice of the DOX group died in the two last weeks of the protocol (44% of mortality), pointing to greater aggressiveness than that observed for MTX, despite the cumulative doses administrated being clinically equivalent [4,5]. Post-mortem examination of these mice revealed pleural effusions and a dilated myocardium, but as they did not reach the final point of the experiment, they were excluded from the analysis.

Considering the remaining animals, none of the drugs changed the whole-body weight (Table 1), despite the DOX group showing a tendency to have this parameter decreased when compared to CTRL (*p* = 0.09). Moreover, no differences were seen for heart weight and tibial length, as well as for the heart weight-to-whole-body weight and heart weight-to-tibial length ratios among groups.

Neither DOX nor MTX influenced the levels of serum total protein, glucose, and albumin compared to the CTRL group (Table 2). Additionally, the activity of ALAT showed a trend to be increased in DOX compared to MTX (*p* = 0.08), despite no significant differences were found when comparing both groups with CTRL. Similarly, no differences were seen in ASAT activity among groups. Nevertheless, the ASAT-to-ALAT ratio showed a tendency to be increased in MTX compared to the CTRL group (*p* = 0.07). In fact, as can be seen in the Supplementary Data, MTX changed the liver structure to a higher extent than DOX (please check Supplementary Data and Figure S1). Regarding the heart, DOX showed a trend to increase the activity of the cardiac isoform of CK, CK-MB, compared to CTRL (*p* = 0.08), while MTX did not affect this marker of cardiac damage.

### 3.2. DOX Had a Higher Effect on Cardiac Structure Than MTX

**Figure 1:**
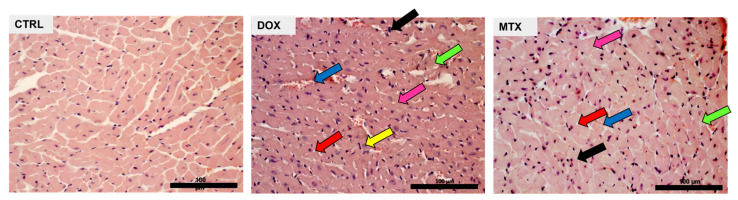
Effect of DOX and MTX on heart structure assessed by light microscopy. Representative light micrographs obtained through hematoxylin and eosin staining from CTRL, DOX, and MTX mice are depicted (representative images of three animals analyzed per group). The CTRL group showed normal morphology and structure. DOX group presented cellular oedema (pink arrow), necrotic zones (green arrow), large and uncondensed nucleus (yellow arrow), vacuolization (red arrow), inflammatory infiltration (black arrow), and vascular congestion (blue arrow). MTX group showed cellular oedema (pink arrow), necrotic zones (green arrow), vacuolization (red arrow), inflammatory infiltration (black arrow), and vascular congestion (blue arrow). Images were taken at 40× magnification (scale bar = 100 µm). For additional quantitative data, please consult references [35] and [36] for DOX and MTX, respectively.

Both DOX and MTX induced structural alterations, as detailed in our previous works using the same experimental paradigm [35,36]. In the heart, DOX led to interstitial oedema, perinuclear vacuolization, and vascular congestion, as well as interstitial inflammatory cell infiltration and marked necrotic zones, demonstrating the ability to cause cardiac disorganization and degeneration (Figure 1). MTX also led to interstitial inflammatory cell infiltration and necrotic zones, along with cytoplasmic vacuolization of cardiomyocytes, cellular oedema, and vascular congestion. Although both drugs influenced cardiac structure, DOX appears to cause more alterations in this experimental paradigm.

### 3.3. DOX Had a Higher Effect on Cardiac Oxidative Metabolism Than MTX

**Figure 2:**
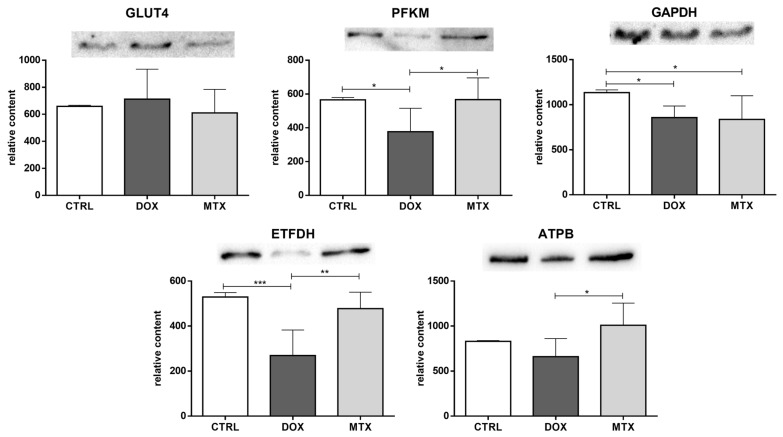
Effect of DOX and MTX on heart metabolism markers when assessed by Western blot in cardiac homogenates. The protein content displayed was normalized to total protein using Ponceau S staining. Representative images of the Western blot obtained are presented, and the Ponceau S staining for each protein is presented in Supplementary Figure S2. Values are expressed as mean ± SD (*n* = 5–6) of relative content, and statistical analyses were performed with one-way ANOVA followed by Tukey’s multiple comparisons test: * *p* < 0.05, ** *p* < 0.01, *** *p* < 0.001. GLUT4: glucose transporter GLUT4, PFKM: phosphofructokinase, GAPDH: glyceraldehyde-3-phosphate dehydrogenase, ETFDH: electron transfer flavoprotein-ubiquinone oxidoreductase, ATPB: ATP synthase subunit β.

The content of the glucose transporter GLUT4 was not changed by any of the drugs tested (Figure 2). PFKM content decreased in the DOX group, while the content of GAPDH was decreased in both DOX and MTX groups compared to CTRL, suggesting the downregulation of glycolysis. DOX group also had decreased ETFDH content compared to CTRL, pointing to the downregulation of FA oxidation, given the role of this dehydrogenase in transferring electrons from FA oxidation to the ubiquinone pool [28]. Additionally, the content of PFKM, ETFDH, and ATPB was decreased in the DOX group compared to MTX, despite no differences found when comparing that group with the CTRL one.

**Figure 3:**
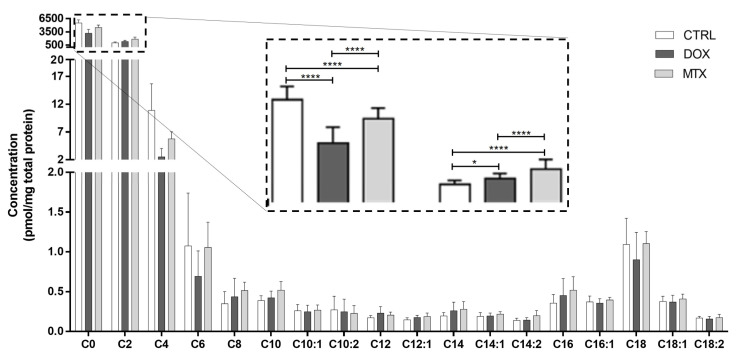
Effect of DOX and MTX on the cardiac acylcarnitine profile assessed by MS/MS. Values are expressed as mean ± SD (*n* = 5–6) of concentration, and statistical analyses were performed with two-way ANOVA followed by Tukey’s multiple comparisons test: * *p* < 0.05, **** *p* < 0.0001. C0: carnitine, C2: acetylcarnitine, C4: butyrylcarnitine, C6: hexanoylcarnitine, C8: octanoylcarnitine, C10: decanoylcarnitine, C10:1: decenoylcarnitine, C10:2: decadienoylcarnitine C12: dodecanoylcarnitine, C12:1: dodecenoylcarnitine, C14: myristoylcarnitine, C14:1: tetradecenoylcarnitine, C14:2: tetradecadienoylcarnitine, C16: palmitoylcarnitine, C16:1: palmitoleoylcarnitine, C18: stearoylcarnitine, C18:1: oleoylcarnitine, C18:2: linoleylcarnitine.

To better explore the modulation of FA oxidation by DOX and MTX, cardiac acylcarnitine profiling was conducted (Figure 3). Both drug-exposed groups decreased the concentration of free carnitine (C0) compared to CTRL. In contrast, acetylcarnitine (C2) was increased in the two groups compared to CTRL. Moreover, the concentration of C0 and C2 was decreased in the DOX group when compared to MTX. For the other acylcarnitines measured, no differences were found among the groups.

**Figure 4:**
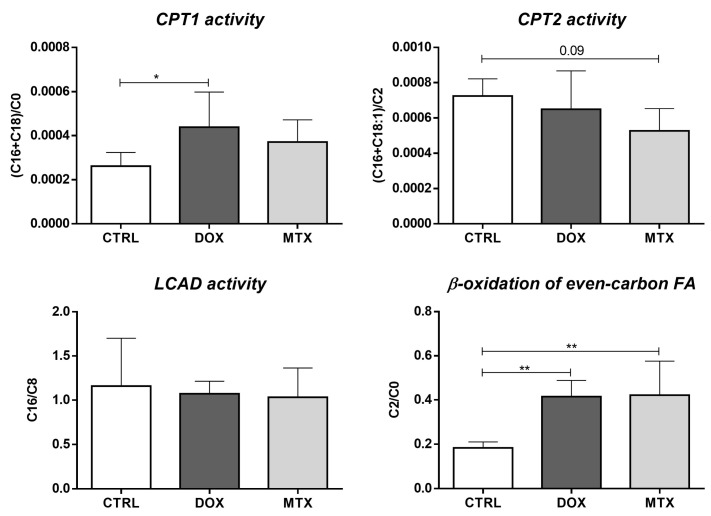
Effect of DOX and MTX on the activity of cardiac enzymes involved in FA oxidation assessed through the ratios among acylcarnitine species: carnitine (C0), acetylcarnitine (C2), octanoylcarnitine (C8), palmitoylcarnitine (C16), stearoylcarnitine (C18) and oleoylcarnitine (C18:1). Values are expressed as mean ± SD (*n* = 5–6), and statistical analyses were performed with one-way ANOVA followed by Tukey’s multiple comparisons test: * *p* < 0.05, ** *p* < 0.01.

Furthermore, the activity of enzymes involved in FA oxidation can be evaluated through the ratios of specific acylcarnitines [45]. Herein, we estimated the activity of carnitine palmitoyl-transferase 1 (CPT1) and 2 (CPT2), long-chain acyl-CoA dehydrogenase (LCAD) along with the β-oxidation of even-carbon FA, using the ratios among C0, C2, octanoylcarnitine (C8), palmitoylcarnitine (C16), stearoylcarnitine (C18) and oleoylcarnitine (C18:1) (Figure 4). DOX group seemed to increase the activity of CPT1, given by the ratio of C16+C18 over C0, while the MTX group showed a tendency to decrease the activity of CPT2 ([C16+C18:1]/C2 ratio, *p* = 0.09). However, no differences were observed for LCAD activity (C16/C8 ratio) after DOX or MTX. Nonetheless, both DOX and MTX seemed to increase the oxidation of FA with even-carbon (C2/C0 ratio).

**Figure 5:**
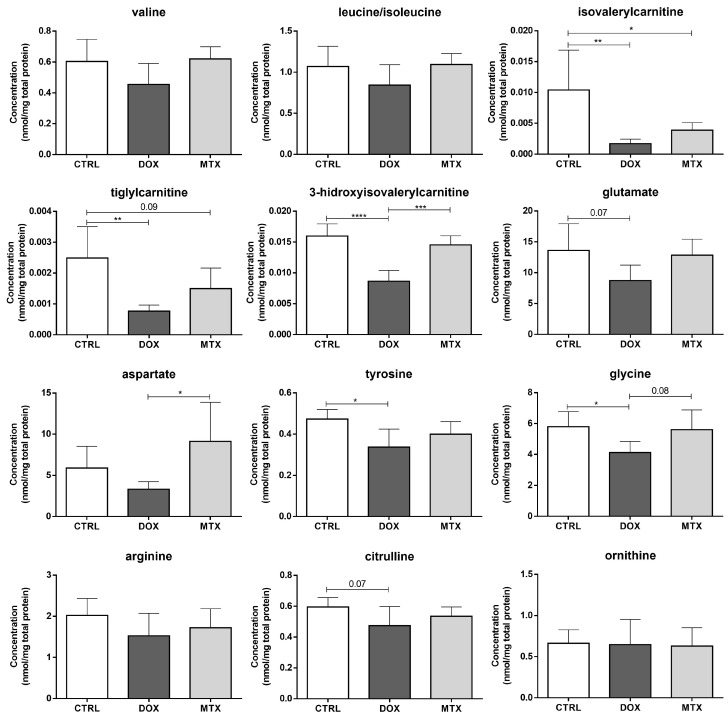
Effect of DOX and MTX on cardiac amino acids profile, when assessed by MS/MS. Values are expressed as mean ± SD (*n* = 5–6) of concentration, and statistical analyses were performed with one-way ANOVA followed by Tukey’s multiple comparisons test: * *p* < 0.05, ** *p* < 0.01, *** *p* < 0.001, **** *p* < 0.0001.

Regarding the amino acids profile after DOX and MTX, no significant differences were found within the following groups regarding the branched-chain amino acids (BCAA): valine, leucine, and isoleucine (Figure 5). Nevertheless, the metabolites isovalerylcarnitine and tiglylcarnitine, which are intermediates in the catabolism of leucine and isoleucine, respectively, were decreased after DOX and MTX compared to the CTRL group. In addition, the 3-hidroxyisovalerilcarnitine, an intermediate derived from the previous ones [46], was significantly decreased in the DOX-treated group compared to CTRL and MTX groups. Moreover, DOX showed a tendency to decrease the concentration of glutamate compared to CTRL (*p* = 0.07), while tyrosine and glycine levels were significantly decreased in the DOX group. Additionally, the concentration of aspartate was decreased in DOX compared to MTX, despite no differences regarding those groups with the CTRL. A trend toward decreased concentration of glycine in the DOX group was seen when compared to MTX (*p* = 0.08). Concerning the arginine-citrulline pathway, no differences were seen for arginine nor for ornithine, despite a tendency for decreased citrulline concentration observed for DOX compared to the CTRL group (*p* = 0.07).

**Figure 6:**
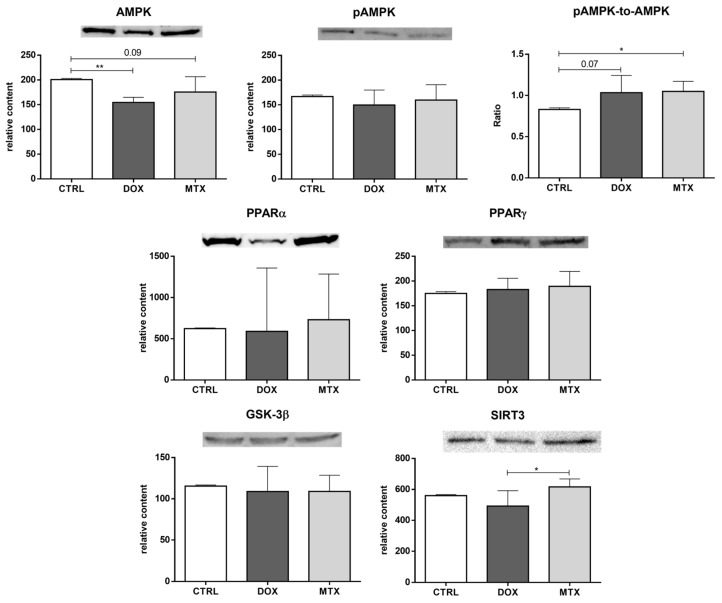
Effect of DOX and MTX on heart metabolism regulation when markers were assessed by Western blot in cardiac homogenates. The protein content displayed was normalized to total protein using Ponceau S staining. Representative images of the Western blot obtained are presented, and the Ponceau S staining for each protein is presented in Supplementary Figure S2. Values are expressed as mean ± SD (*n* = 5–6) of relative content, and statistical analyses were performed with one-way ANOVA followed by Tukey’s multiple comparisons test: * *p* < 0.05, ** *p* < 0.01. AMPK: AMP-activated protein kinase, pAMPK: AMPK phosphorylated, PPARα: peroxisome proliferator-activated receptor α, PPARγ: peroxisome proliferator-activated receptor γ, GSK-3β: glycogen synthase kinase 3 β, SIRT3: sirtuin 3.

Additionally, several metabolic modulators of the previous energy pathways were assessed (Figure 6). DOX decreased the content of AMPK, while MTX induced a tendency towards decreased values of this sensor of cellular energy status [47], compared to the CTRL group (*p* = 0.09). Despite no differences for pAMPK content, MTX increased the pAMPK-to-AMPK ratio, and DOX showed a tendency towards increased values of this ratio compared to the CTRL group (*p* = 0.07), which is suggestive of AMPK activation. For the other metabolic modulators evaluated, PPARα, PPARγ, and GSK-3β, no differences were found among the groups. Additionally, the content of SIRT3 was decreased in the DOX group compared to MTX, despite no differences found when comparing those groups with the CTRL group.

### 3.4. DOX Affected Cardiac Mitochondrial Biogenesis Differently from MTX

**Figure 7:**
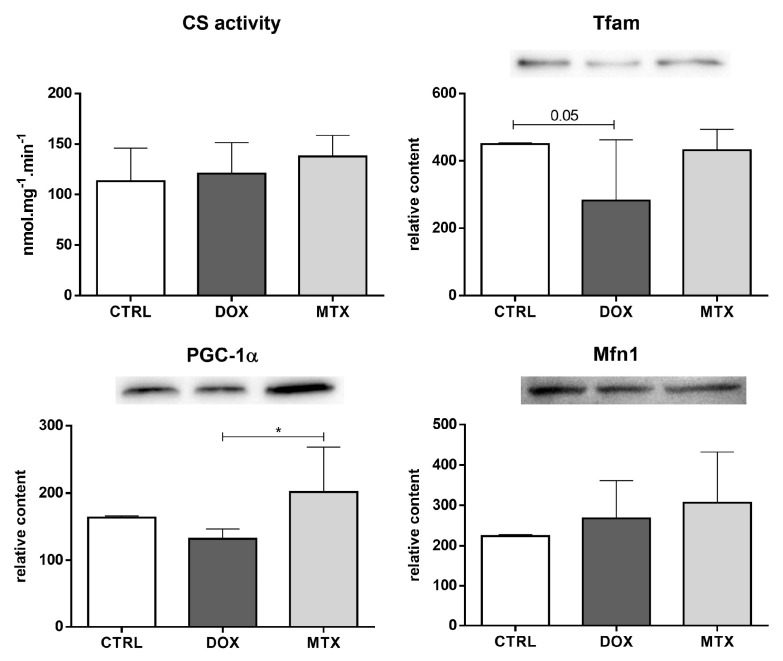
Effect of DOX and MTX on heart mitochondrial density and biogenesis when markers were assessed by a spectrophotometric assay (CS activity) and Western blot in cardiac homogenates. The protein content displayed was normalized to total protein using Ponceau S staining. Representative images of the Western blot obtained are presented, and the Ponceau S staining for each protein is presented in Supplementary Figure S2. Values are expressed as mean ± SD (*n* = 5–6) of activity or relative content, and statistical analyses were performed with one-way ANOVA followed by Tukey’s multiple comparisons test: * *p* < 0.05. CS: citrate synthase, Tfam: mitochondrial transcription factor A, PGC-1α: peroxisome proliferator-activated receptor γ coactivator 1 α, Mfn1: mitofusin1.

The activity of CS, which is considered a rough marker of mitochondrial density [48], was not altered in any of the drug-exposed groups (Figure 7). Nevertheless, the content of Tfam was decreased in the DOX group compared to CTRL. No other differences regarding the mitochondrial biogenesis markers, namely on PGC-1α and Mfn1, were seen when comparing the drugs to the CTRL group. However, DOX had decreased content of PGC-1α compared to the MTX group.

### 3.5. MTX Had a Bigger Impact on Cardiac Autophagy Than DOX

**Figure 8:**
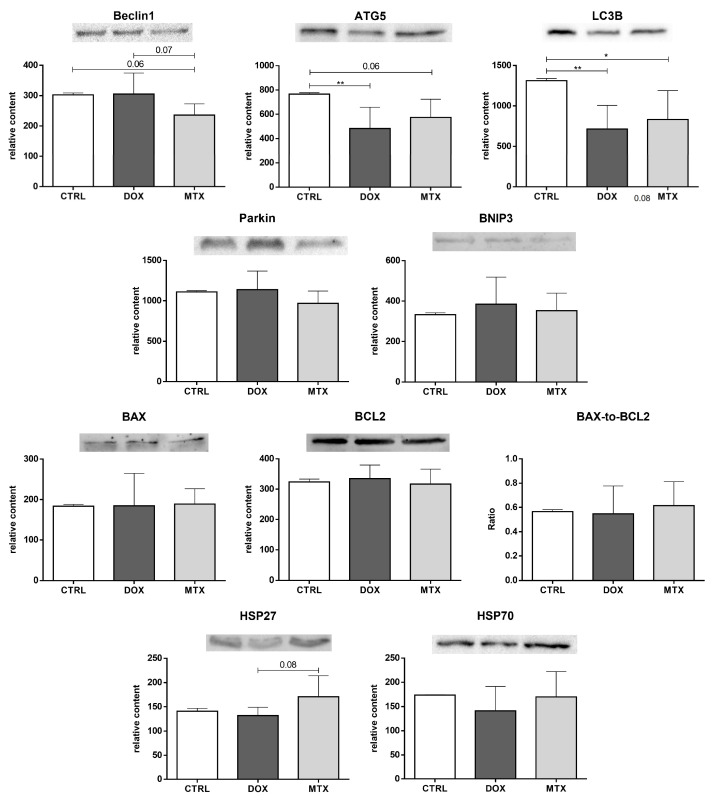
Effect of DOX and MTX on heart autophagy and apoptosis markers when assessed by Western blot in cardiac homogenates. The protein content displayed was normalized to total protein using Ponceau S staining. Representative images of the Western blot obtained are presented, and the Ponceau S staining for each protein is presented in Supplementary Figure S2. Values are expressed as mean ± SD (*n* = 5–6) of relative content, and statistical analyses were performed with one-way ANOVA followed by Tukey’s multiple comparisons test: * *p* < 0.05, ** *p* < 0.01. ATG5: autophagy protein 5, LC3B: microtubule-associated protein light chain 3, BNIP3: BCL2 interacting protein 3, BAX: BCL2 associated X-protein, BCL2: B-cell lymphoma-2, HSP27: heat shock protein 27 kDa, HSP70: heat shock protein 70 kDa.

Beclin1 content showed a trend to be exclusively decreased by MTX compared to the CTRL group (*p* = 0.06, Figure 8). On the contrary, the content of ATG5 was decreased in the DOX group, although MTX showed a trend to decrease it compared to the CTRL group (*p* = 0.06). Both DOX and MTX groups had decreased LC3B content compared to CTRL. Additionally, the MTX group showed a trend to decrease the content of Beclin1 compared to the DOX group (*p* = 0.07). No differences were found in the content of Parkin and BNIP3 among groups.

Moreover, for the pro-apoptotic, BAX, and anti-apoptotic, BCL2, proteins, as well as for their ratio, no significant changes were observed. In addition, MTX showed a tendency to increase the content of HSP27 compared to the DOX group (*p* = 0.08), but no differences were found when comparing it with the CTRL group. No changes were found for the HSP70 content among the groups.

## 4. Discussion

Both DOX and MTX lead to cardiotoxic effects that may progress to heart failure [6,7,8]. Despite the similarities between these two anticancer agents in the pharmacodynamics and clinical evidence of cardiotoxicity, the mechanisms behind their toxicity seem to diverge [18]. In this work, the cardiac molecular remodeling after DOX and MTX using clinically relevant and pharmacologically equivalent doses of both agents (18 and 6 mg/kg, respectively) was assessed. The selection of these doses was based on the isotoxic/pharmacokinetic dose conversion between DOX and MTX, assuming that one molecule of MTX is known to have similar anticancer and cardiotoxic effects to three molecules of DOX [4,5,6]. Nevertheless, four mice of the DOX group died during the protocol (44% of mortality), suggesting a greater impact of DOX on the animal’s survival/toxicity than MTX, at least considering the end-time selected in this study.

The administration of the two drugs did not significantly affect the morphometric parameters of the animals, although DOX showed some tendency to decrease whole-body weight. Indeed, at sacrifice, DOX and MTX animals presented ascites that may have masked significant decreases in body weight. Moreover, the administration of DOX and MTX did not affect the concentration or activity of most biochemical parameters measured in serum. Despite no statistical differences seen among groups, the CK-MB activity showed a trend to be increased in DOX, being suggestive of heart damage [49]. For MTX, the findings observed for CK-MB activity were like the ones in previous studies of our group and no meaningful changes were seen [33,36]. Nevertheless, damage to cardiac tissue was observed after both drugs considering the histological assessment, with DOX appearing to cause more structural alterations, which is in line with the CK-MB results. Data reported in the literature showed links between alterations in cardiac structure and perturbed function of the heart after 6–7.5 mg/kg and 3–30 mg/kg cumulative doses of MTX and DOX, respectively [9,10,11,12,13,25,32,35,36]. These findings show that other markers of damage may be more sensitive toward the injury inflicted on the heart rather than the classical parameters detected in serum, as we did not see any meaningful differences in the classical serum markers of cardiotoxicity, for instance, on MTX.

**Figure 9:**
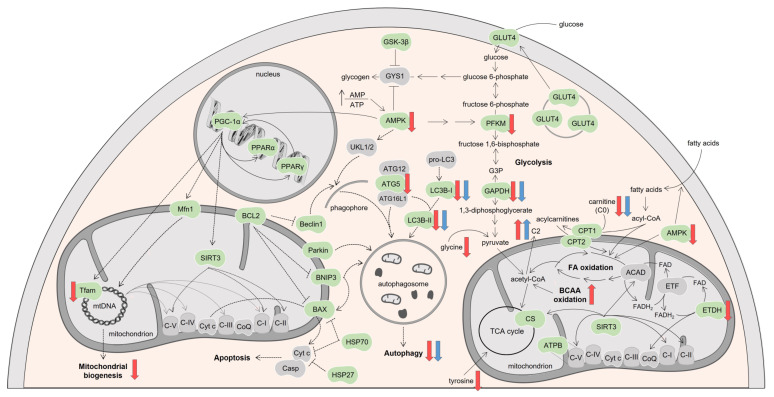
Schematic representation of the molecular pathways modulated by DOX and MTX. Proteins searched in this study are indicated in green, while the ones in grey belong to the pathway but were not investigated. Proteins found upregulated (up arrows) or downregulated (down arrows) by DOX or MTX compared to CTRL are indicated with red or blue arrows, respectively. Figure was made with *Servier medical art*. ACAD: acyl-CoA dehydrogenases, AMP: adenosine monophosphate, AMPK: AMP-activated protein kinase, ATG: autophagy protein, ATP: adenosine triphosphate, ATPB: ATP synthase subunit β, BAX: B-cell lymphoma-2 associated X-protein, BCAA: branched-chain amino acids, BCL2: B-cell lymphoma-2, BNIP3: BCL2 interacting protein 3, C2: acetylcarnitine, C-I: nicotinamide adenine dinucleotide-ubiquinone oxidoreductase, C-II: succinate-ubiquinone oxidoreductase, C-III: ubiquinone-cytochrome c oxidoreductase, C-IV: cytochrome c oxidase, C-V: F1Fo ATP synthase, Casp: caspase, CoQ: ubiquinone, CPT: carnitine palmitoyl-transferase, CS: citrate synthase, Cyt c: cytochrome c, ETF: electron transfer flavoprotein, ETFDH: ETF-ubiquinone oxidoreductase, FA: fatty acids, FAD: flavin adenine dinucleotide, FADH2: reduced form of FAD, G3P: glyceraldehyde-3-phosphate, GAPDH: G3P dehydrogenase, GLUT4: glucose transporter, GSK-3β: glycogen synthase kinase 3 β, GYS1: muscle glycogen synthase, HSP: heat shock protein, LC3B: microtubule-associated protein light chain 3, Mfn1: mitofusin1, mtDNA: mitochondrial DNA, PFKM: phosphofructokinase, PGC-1α: peroxisome proliferator-activated receptor γ coactivator 1 α, PPAR: peroxisome proliferator-activated receptor, SIRT3: sirtuin 3, TCA: tricarboxylic acid, Tfam: mitochondrial transcription factor A, ULK1/2: complex of serine/threonine-protein kinases ULK1 and ULK2.

As stated in the introduction section, cardiac function depends predominantly on the ATP produced by mitochondrial oxidative phosphorylation (OXPHOS) using FA as the main source of reducing equivalents. Nevertheless, other substrates, such as glucose and amino acids, may also be used as energy substrates [27,28]. Glucose is transported into cardiomyocytes by glucose transporters, mainly GLUT4 (Figure 9). This transporter locates in intracellular storage vesicles, being translocated to the plasma membrane upon insulin or other stimulation, and represents an important mechanism of cardiac cells to regulate glucose uptake in response to body changes [50]. We saw no differences in the GLUT4 transporter after the administration of the two drugs; however, the levels of GLUT4 may not reflect its density at the membrane since whole heart muscle extracts were used for its assessment. Still, the content of the glycolytic enzymes PFKM and GAPDH was decreased. PFKM is responsible for the irreversible phosphorylation of fructose 6-phosphate to fructose 1,6-bisphosphate (1,6-BP), while GAPDH catalyzes the interconversion of glyceraldehyde-3-phosphate (G3P), originated by aldolase from fructose 1,6-BP, into 1,3-diphosphoglycerate [51,52]. DOX decreased both PFKM and GAPDH, whereas MTX only decreased GAPDH. Therefore, DOX seems to have a higher impact on the glycolytic pathway than MTX, blunting its use. These data seem to contradict other works that have reported upregulation of glycolysis after DOX as a consequence of the decline in the oxidation of FA [41,42,53,54]. These works, however, were performed in rats using a higher cumulative dose and a different approach from our study to assess the glycolysis flux [41,53] or in distinct cellular models derived from rat [54] and human [42] cardiac cells.

Moreover, administration of DOX decreased the content of ETFDH, but the increased ratio between C2 and C0 suggests increased oxidation of FA of even-carbon. In addition, the activity of CPT1 was increased by DOX, whereas CPT2 activity showed a trend to be decreased by MTX. These proteins are essential for the transport of LCFA across the mitochondrial membrane. CPT1 converts the acyl-CoA ester derived from LCFA to their corresponding acylcarnitine metabolites at the outer membrane, while CPT2 recovers them from carnitine on the inner membrane [55]. Nonetheless, the activity of these enzymes was inferred based on the ratios among long-chain acylcarnitines (C16, C18, and C18:1) and C0 and C2 [45]. Since no meaningful changes were observed for these long-chain acylcarnitines, the differences in CPT1 and CPT2 activities may be originated from important changes in C0 and C2. Furthermore, neither variations were found in the remaining acylcarnitines assessed (C4 - C18:2) nor for the activity of LCAD, extrapolated by the ratio between C16 and C8 [45]. Moreover, no differences were seen for OXPHOS after DOX or MTX given by ATPB content, which is one subunit of the catalytic part (F1) of the F1Fo ATP synthase (complex V, C-V) (35). In other works, this subunit was previously reported as downregulated after both DOX or MTX in heart mitochondria of male mice [32], with DOX also downregulating the ATPB expression in ventricular cardiomyocytes derived from Sprague-Dawley rats [41] and in human cardiac 3D microtissues [56].

Despite acylcarnitines being the main energy source for cardiac muscle [28], it seems that alternative pathways may be used in our cardio-oncology protocol. Amino acids are an alternative energy source for cardiac muscle [28]. Although no differences in valine, leucine, and isoleucine levels were found, the concentration of the resulting metabolites isovalerylcarnitine and tiglylcarnitine was decreased by both DOX and MTX. The former amino acids, while not changed, may act as regulators of other energy molecules [57]. Altogether, the data suggest increased import of BCAA and/or muscle proteolysis, as previously reported for DOX [58]. The 3-hidroxyisovalerylcarnitine concentration was also significantly decreased by DOX, pointing to a bigger impact of DOX on cardiac metabolism. This metabolite is an intermediate formed from 2-methylcrotonyl-CoA or tiglyl-CoA, which are the products of the third step of leucine or isoleucine metabolism, respectively. Isovalerylcarnitine is derived from isovaleryl-CoA, which is the metabolite that originates 2-methylcrotonyl-CoA on leucine catabolism. Similarly, tiglylcarnitine is the corresponding acylcarnitine of tiglyl-CoA that results from the catabolism of isoleucine [46].

Other amino acids may fuel the cardiac cells, such as glutamate and aspartate, which are converted into α-ketoglutarate and oxaloacetate, the key players in the tricarboxylic acid (TCA) cycle [59,60]. Indeed, increased uptake of glutamate was previously reported in heart failure [61]. DOX showed a trend for decreased glutamate concentration compared to CTRL, with no differences for aspartate. No differences were seen for MTX for either amino acid. The metabolism of tyrosine, whose concentration was decreased after DOX administration, also results in TCA cycle intermediates, namely fumarate. Conversely, the metabolism of alanine and glycine results in pyruvate production [59,60]. Glycine concentration was decreased by DOX treatment. Regarding the arginine-citrulline pathway that is important to keep ROS at controlled levels [62], no remarkable differences were discovered herein, although DOX showed a trend to decrease citrulline concentration. Thus, it seems that amino acids may represent a complementary energy source in cardiac muscle after DOX administration.

AMPK is a key enzyme that regulates the activity of metabolic proteins involved in lipid and glucose oxidation by phosphorylating them. For instance, AMPK phosphorylates and activates phosphofructokinase 2, the enzyme that converts fructose 6-phosphate to fructose-2,6-diphosphate, being this last stimulator of PFKM. Thus, AMPK activates glycolysis [47]. Herein, decreased content of AMPK was observed after administration of DOX (Figure 9), suggesting a pivotal role of DOX for affecting energetic metabolism. Indeed, downregulation of AMPK was previously reported in a human cardiac cell line incubated with DOX, with no similar findings for MTX [18,42].

A key nuclear target of AMPK is PGC-1α, which, once activated, interacts with other nuclear transcriptional factors such as PPARα and PPARγ and regulates the expression of several genes that encode for proteins involved in FA and glucose utilization [47,50,63]. We saw no differences in PGC-1α compared to the CTRL group; thus, it was no surprise that no changes in PPARα and PPARγ were found after the administration of DOX and MTX (Figure 9). Similarly, no meaningful differences in the content of GSK-3β were observed after the administration of the two drugs. This enzyme plays important roles in heart development and function, and also in the regulation of the muscle glycogen synthase (GYS1), which acts on glycogen production [51,64,65]. Moreover, the content of the deacetylase SIRT3, which regulates the activity of key proteins of FA oxidation and OXPHOS, such as ATPB and other subunits of OXPHOS complexes I, II, and V [66], showed no differences compared to the CTRL group.

The administration of the anticancer drugs did not alter the activity of CS, a rough indicator of mitochondrial density [48], which reflects the balance between biogenesis and clearance [67]. Therefore, it may be proposed that mitochondrial number is not altered in response to DOX and MTX. Nevertheless, in a previous study from our group, CS activity decreased after a lower dose of DOX (9 mg/kg) and an equal dose of MTX. In that study, we also used an aged mice (18–20 months old) group that did not receive any agent [32]. A higher number of animals in the CS activity assessment was used in that study compared to the animals used herein. Thus, such differences may not be strictly compared between the two studies because of the added group when statistics are made [32]. Decreased content of Tfam was observed after DOX administration compared with the CTRL group (Figure 9). This mitochondrial factor is the main regulator of the copy number of mitochondrial DNA (mtDNA) that is indicative of mitochondrial transcription [68]. Therefore, decreased expression of Tfam suggests reduced mtDNA expression and, eventually, downregulation of OXPHOS in cardiac cells after DOX. Tfam is regulated at the transcriptional level by the coactivator PGC-1α along with other nuclear factors, such as nuclear respiratory factors (NRF) 1 and 2 [47,68]. Although no differences were found for PGC-1α in DOX compared to CTRL, the content of NRF was not assessed, which may suggest a bigger role of the last factors for the coactivation of Tfam. PGC-1α is also a transcriptional coactivator of Mfn1, which, among other proteins, regulates mitochondrial fusion and leads to elongated interconnected networks of mitochondria with diverse mtDNA [69]. As for PGC-1α, no differences were found for Mfn1 after the administration of the two anticancer agents when compared to the CTRL group. Moreover, PGC-1α regulates the expression of SIRT3, which besides its action on mitochondrial metabolism, also stimulates mitochondrial biogenesis, enhancing mitochondrial efficiency [63,68]. As stated, we had no differences in PGC-1α reporting to CTRL, but mitochondrial biogenesis is balanced by autophagy, which can be seen as a mitochondrial quality process [47,67,68,69,70] that we researched next.

Under stress conditions, such as increased AMP/ATP ratio, AMPK is activated and may phosphorylate the complex of serine/threonine-protein kinases ULK1 and ULK2 (ULK1/2), which is essential for the induction of autophagy. It was previously reported that anthracyclines inhibit AMPK, blocking the initiation of autophagy [71]. The phosphorylation of this complex leads to its activation and translocation to the membrane of the endoplasmic reticulum, where it recruits the Beclin1 complex [47,63,70]. This complex produces phosphatidylinositol-3-phosphate that is located on the phagophore surface and consequently allows the recruitment of other autophagic proteins [70]. In this study, ULK1/2 was not assessed, but the content of Beclin1 showed a tendency to be decreased after administration of MTX, while no impact was found for DOX, evidencing distinct modulation of this protein by the two drugs (Figure 9). Despite not being statistically significant, this finding is in line with a recent study of the group that demonstrated that MTX decreased the content of Beclin1 on human differentiated AC16 cells [72], thus suggesting the blockage of this route of autophagy by MTX.

Next, in the autophagy process, the phagophores formed are elongated by ubiquitin-like proteins that work in a coordinated fashion to sequester cytoplasmic constituents and organelles inside the autophagosomes. ATG5 is conjugated with other ATG proteins, namely ATG12 and ATG16L1, to form a complex that expands the phagophore membrane [70]. This complex also helps in the movement of LC3B-I (cytosolic form) to the phagophore membrane and in its conjugation with phosphatidylethanolamine to LC3B-II in the membrane of the autophagosome. Therefore, the content of LC3B-II correlates directly with the extent of autophagosome formation, thus making it an autophagosome marker [70,73]. The administration of both DOX and MTX decreased the content of LC3B (Figure 9). Additionally, DOX decreased the ATG5 content, while MTX only showed a trend for its decrease. In the literature, both upregulation and downregulation of autophagy proteins have been reported after DOX, depending on the models used, while for MTX, little is known [42,71,74,75,76]. Nevertheless, ATG5 content was found to decrease for 1, 5, and 10 μM of MTX on human differentiated AC16 cells, with MTX also affecting other autophagy proteins [72]. Herein, we showed that both DOX and MTX blunt autophagy, although we cannot determine if it was protective or deleterious to the heart.

Specific players of mitophagy that consist of the elimination of damaged mitochondria, namely Parkin and BNIP3, were researched [77,78,79], but no differences were found in their content after the administration of both anticancer agents (Figure 9). Briefly, Parkin and BNIP3 may localize in the outer mitochondrial membrane where they connect with LC3B-II and thus initiate the autophagy of damaged mitochondria [77,78,79]. Despite no meaningful differences for these mitophagy markers, they have been associated with anthracycline-induced cardiotoxicity [71]. Even so, in our experimental study, we assessed important autophagy proteins, namely Beclin1, ATG5, and LC3B, which act at different steps, as well as one of the greatest regulators of autophagy, the AMPK. Thus, to some extent, we searched for the several pathways of this biological process and observed accordance among them, being able to infer that both anticancer agents decreased autophagy. Namely, DOX disturbed the elongation of the phagophore and formation of LC3B-II and thus the autophagosome formation, while MTX impacted these steps and phagophore formation (although not significantly), critical for the initiation of the autophagy process. No decrease was observed for CS activity after DOX and MTX administration but decreased content of Tfam points to reduced mitochondrial biogenesis. Therefore, it can be presumed that mitochondrial number is not being decreased because autophagy is occurring to a lesser extent, but possibly an accumulation of damaged mitochondria instead of their elimination is occurring. The accumulation of damaged mitochondria is also corroborated by the elevated number of alterations noted for cardiac metabolism, mainly in DOX.

Regardless of the role of BNIP3 on mitophagy, it also plays a part in the programmed cardiac cell death through the intrinsic/mitochondrial pathway. Indeed, once in the mitochondrial outer membrane, BNIP3 stimulates the pore formation by pro-apoptotic proteins, such as BAX, which leads to membrane permeabilization allowing cytochrome c (Cyt c) release and apoptosis activation [78]. DOX and MTX did not affect the BAX content being in accordance with the lack of differences observed for BNIP3. Moreover, BNIP3 heterodimerizes with anti-apoptotic proteins, namely BCL2, which blocks the activation of BAX [78]. As for BAX and BNIP3, no differences were detected in the BCL2 content after the administration of the two drugs (Figure 9), suggesting no impact of DOX and MTX on the mitochondrial apoptotic pathway in our *in vivo* model, although some studies suggest a correlation between cardiac apoptosis, autophagy, and chemotherapy [29,31,78].

HSP proteins have plenty of cardiac functions, such as protein proper folding and trafficking across organelles [68,80,81,82]. We assessed the content of HSP27, which has been described as an anti-apoptotic protein since it interacts with Cyt c and prevents the activation of caspases, as well as the release of other pro-apoptotic effectors [80]. No significant differences were found after DOX and MTX administration (Figure 9); however, in a previous study, overexpression of HSP27 was found to block apoptosis and, therefore, to protect the cardiac function against heart damage caused by DOX [81].

Additionally, no differences in HSP70 content were observed after the administration of DOX and MTX. HSP70 has been associated with the blocking of several steps in the apoptotic process, namely the ones played by BAX, Cyt c, and caspases [68,83]. A previous study, where DOX was given to male mice, suggested that HSP70 is cardioprotective because it inhibits the mitochondrial apoptotic pathway harshly activated by DOX [84]. Besides its role in apoptosis, HSP70 appears to be involved in the fusion of autophagosomes with endosomes and lysosomes to form the autolysosomes that ultimately degrade their matter, the final step of autophagy [70]. Nonetheless, the contribution of HSP70 to autophagy is smaller compared to the other autophagy proteins assessed [70], which may explain the absence of changes for HSP70 herein.

## 5. Conclusions

To conclude, both DOX and MTX affected differently cardiac molecular remodeling, although both agents decreased cardiac glycolysis through decreased content of GAPDH. Moreover, DOX and MTX decreased the concentration of C0 and increased C2, suggesting more reliance on FA oxidation. Both drugs also decreased the concentration of intermediates of isoleucine and leucine catabolism and the content of AMPK, the main regulator of metabolic status, highlighting metabolic disturbances, although with DOX impacting AMPK, and thus the overall energetic metabolism, to a greater extent. Nevertheless, DOX strongly affected the cardiac metabolism since it exclusively decreased the PFKM content and the concentration of several amino acids, suggesting downregulation of glycolysis and more dependency on the later metabolites as energy sources. The findings for Tfam suggest decreased mitochondrial biogenesis after DOX. Both anticancer agents downregulated autophagy given by the decreased content of ATG5 and LC3B, suggestive of the accumulation of dysfunctional mitochondria when linked to other data of the work. In addition, MTX also showed a predisposition to decrease the content of Beclin1, essential for the initiation of the autophagy process and consequently implying a distinct impact of MTX on this cellular process. Therefore, despite DOX and MTX presenting similar clinical cardiotoxic manifestations and both affecting the energetic metabolism and autophagy process, the cardiac molecular remodeling was differently stimulated. Namely, it seems that DOX influenced energetic metabolism with a greater magnitude and MTX the autophagy process. Thus, more studies comparing both agents are needed to better elucidate the distinct molecular mechanism behind their cardiotoxicity and take adequate and drug-related protective measures.

## Figures and Tables

**Table 1:** Morphometric parameters measured one week after the last i.p. injection. Values are expressed as mean ± SD (*n* = 9 for CTRL and MTX, *n* = 5 for DOX), and statistical analyses were performed with the one-way ANOVA followed by Tukey’s multiple comparisons test.

Morphometric Parameter	CTRL	DOX	MTX
Whole-body weight (g)	42.671 ± 3.450	37.727 ± 6.163	41.264 ± 2.906
Heart weight (g)	0.228 ± 0.048	0.229 ± 0.037	0.227 ± 0.031
Tibial length (cm)	1.90 ± 0.08	1.84 ± 0.05	1.84 ± 0.07
Heart weight-to-whole-body weight (mg/g)	5.30 ± 0.81	6.35 ± 2.20	5.49 ± 0.63
Heart weight-to-tibial length (g/cm)	0.120 ± 0.025	0.125 ± 0.021	0.123 ± 0.016

**Table 2:** Biochemical parameters measured on blood serum. Values are expressed as mean ± SD (*n* = 9 for CTRL and MTX, *n* = 5 for DOX) of levels or activity, and statistical analyses were performed with the one-way ANOVA followed by Tukey’s multiple comparisons test.

Serum Biochemical Parameter	CTRL	DOX	MTX
Total protein (g/L)	47.84 ± 3.42	42.72 ± 3.34	43.76 ± 6.26
Glucose (mg/dL)	359.2 ± 57.47	269.7 ± 115.0	333.2 ± 102.0
Albumin (g/L)	38.41 ± 2.15	36.28 ± 2.22	36.31 ± 4.29
ALAT (U/L)	48.41 ± 19.61	70.52 ± 30.02	40.06 ± 23.89
ASAT (U/L)	26.31 ± 7.42	41.00 ± 16.64	31.09 ± 13.67
ASAT-to-ALAT (U/L)	0.62 ± 0.24	0.63 ± 0.22	0.89 ± 0.27
CK-MB (U/L)	66.40 ± 20.36	92.44 ± 26.60	76.50 ± 16.50

ALAT: alanine aminotransferase, ASAT: aspartate aminotransferase, CK-MB: creatine kinase-MB.

## Data Availability

The data presented in this study are available on request from the corresponding author.

## Supplementary Materials

The following supporting information can be downloaded at: https://www.mdpi.com/2218-273X/13/6/921/s1, Supplementary Data: Histological assessment of liver damage; Figure S1: Effect of DOX and MTX on the liver structure assessed by light microscopy. Representative light micrographs obtained from three animals per group were subjected to hematoxylin and eosin staining. The CTRL group showed normal morphology and structure. DOX and MTX groups presented necrotic zones (green arrow), vacuolization (microvesicular steatosis, red arrow), sinusoidal dilation (orange arrow), inflammatory infiltration (black arrow), and vascular congestion (blue arrow). Images were taken at 40× magnification (scale bar = 100 μm); Figure S2: Ponceau S and Western blots images for each protein evaluated; Table S1: Specification of the dilutions and catalog number for each primary antibody.
