# Supplementary material for: Cardiac Molecular Remodeling by Anticancer Drugs: Doxorubicin Affects More Metabolism While Mitoxantrone Impacts More Autophagy in Adult CD-1 Male Mice
Source: Biomolecules. 2023 May 31;13(6):921. doi: 10.3390/biom13060921 (PMC10296231; doi:10.3390/biom13060921)

GLUT4 - Ponceau S

This study

Samples order from the molecular weight marker: **MTX | DOX18 | CTRL** | DOX9-1 | CTRL-2 | DOX9-2

Other study

1

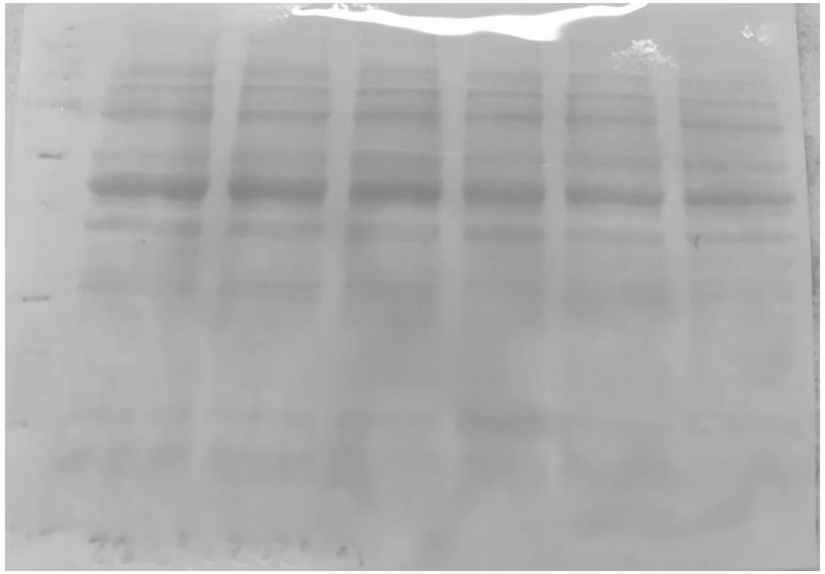

2

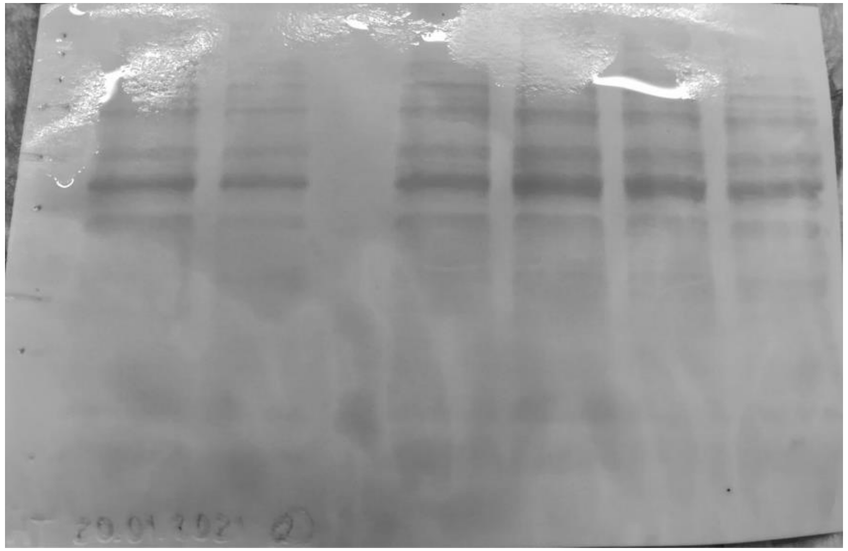

3

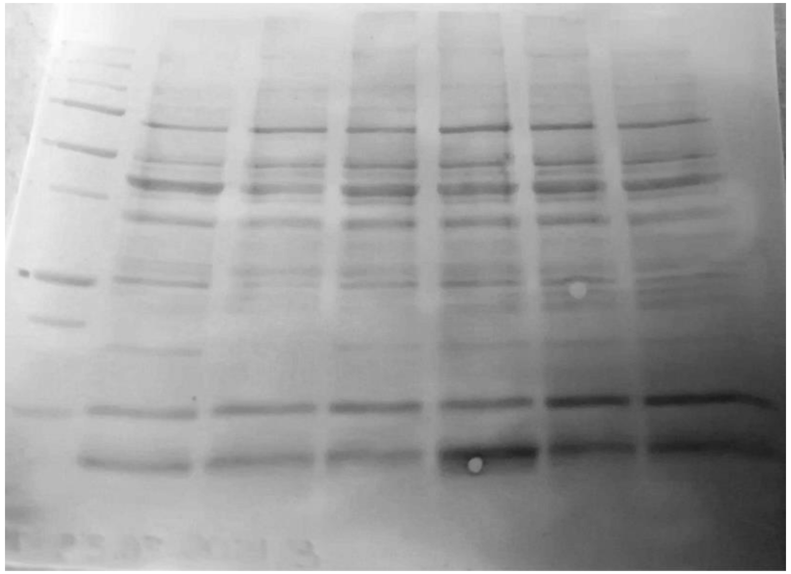

4

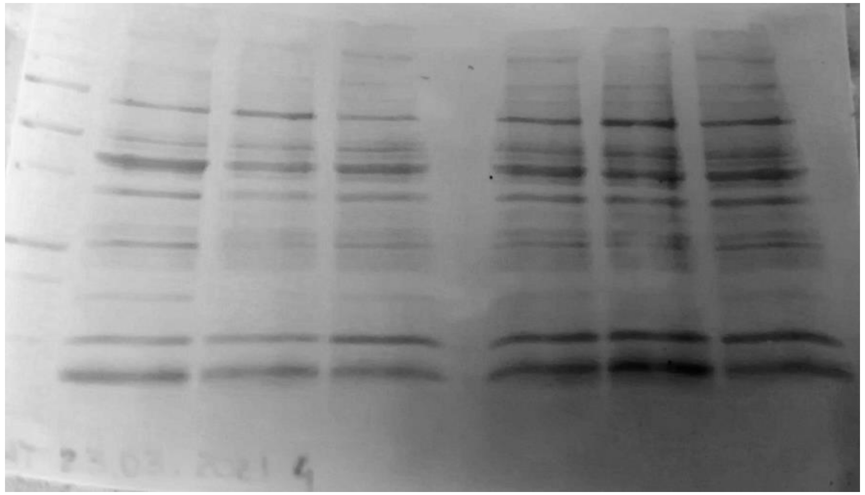

5

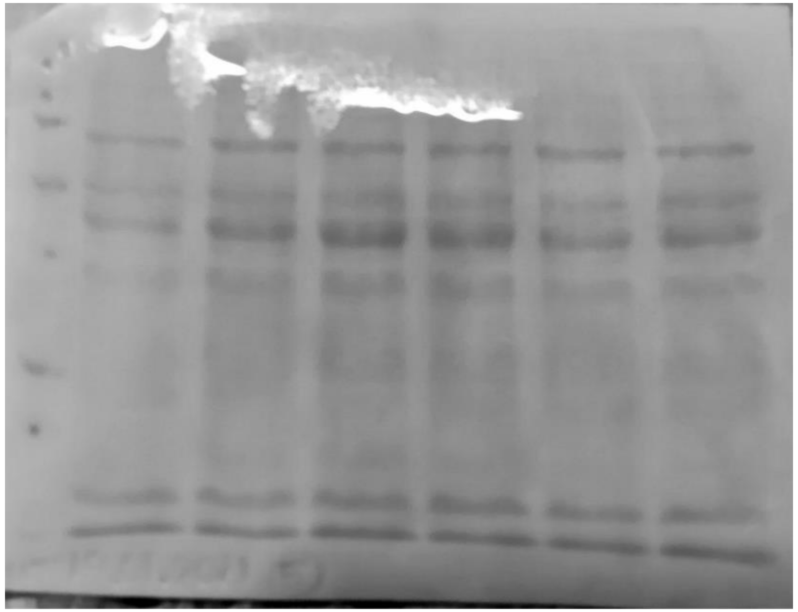

6

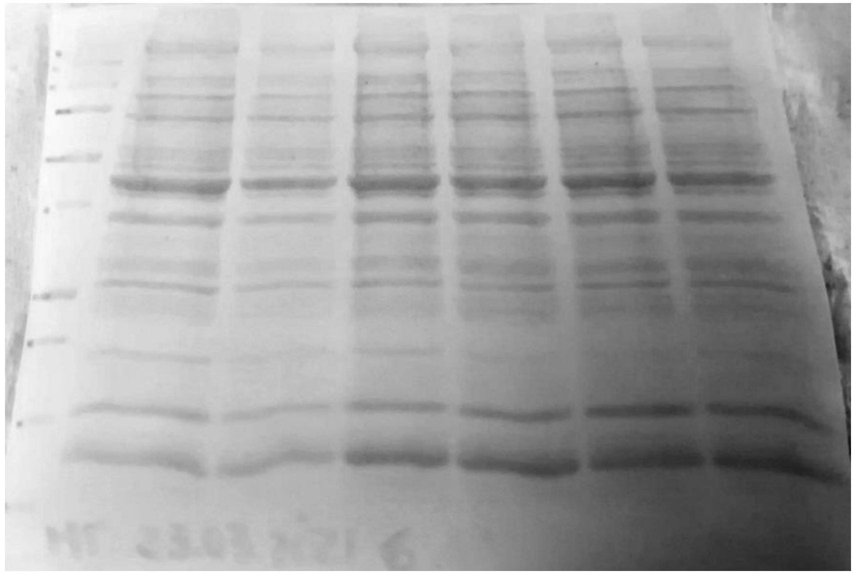

GLUT4 - Blot

This study

Samples order from the molecular weight marker: **MTX | DOX18 | CTRL** | DOX9-1 | CTRL-2 | DOX9-2

Other study

1

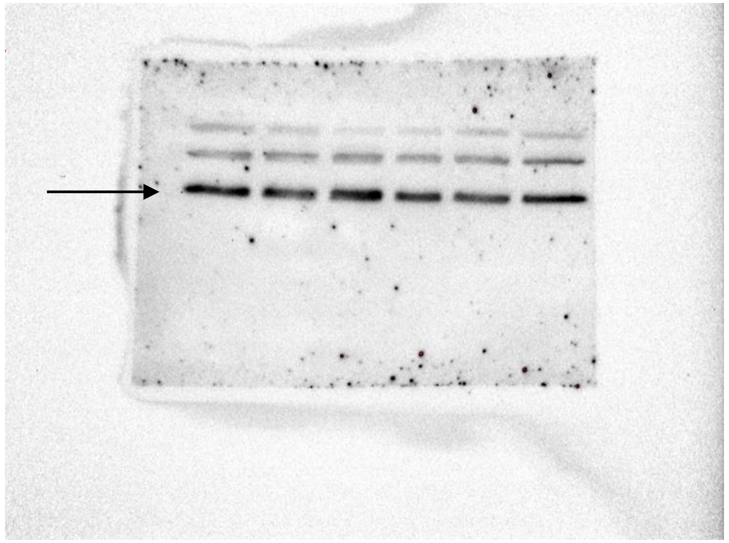

2

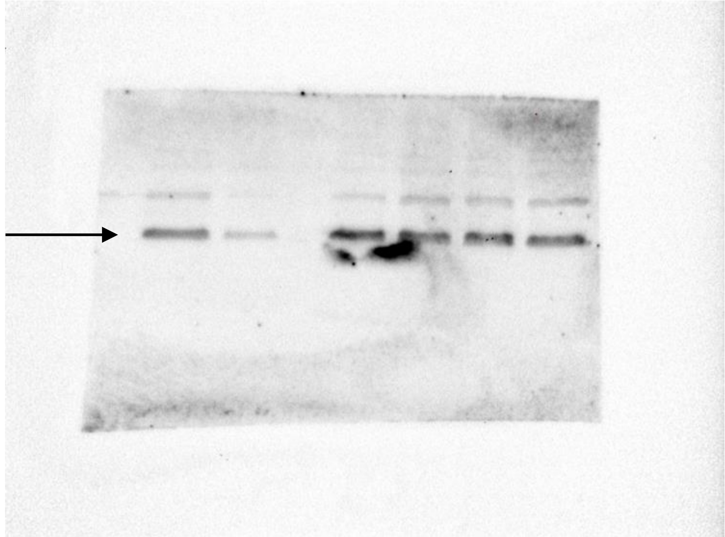

3

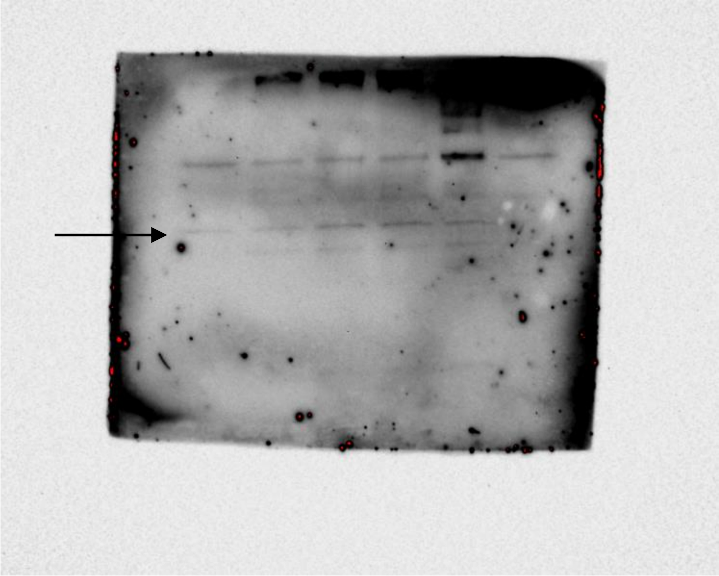

4

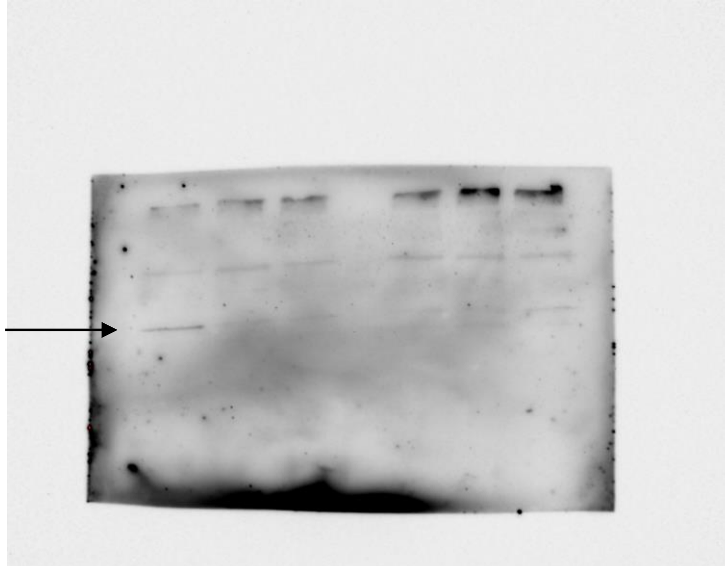

5

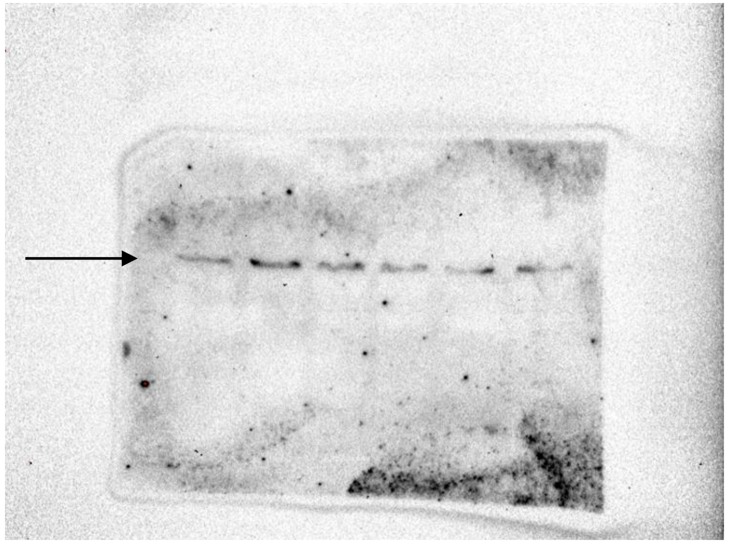

6

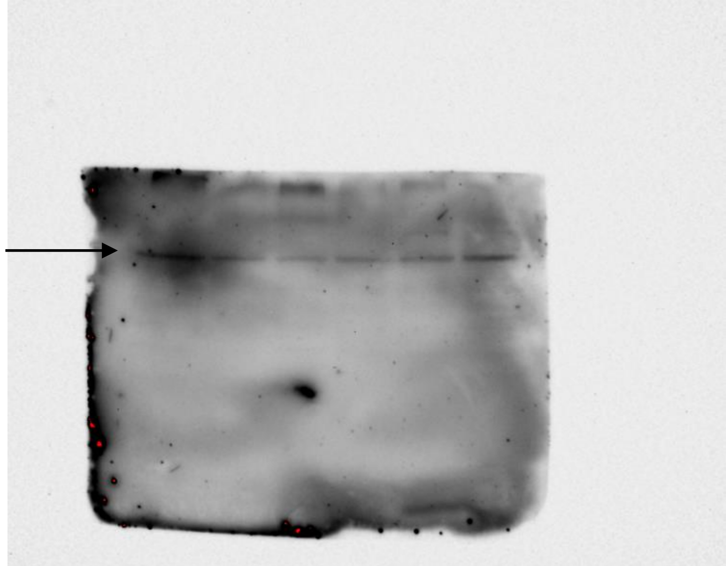

PFKM - Ponceau S

This study

Samples order from the molecular weight marker: **MTX | DOX18 | CTRL** | DOX9-1 | CTRL-2 | DOX9-2

Other study

1

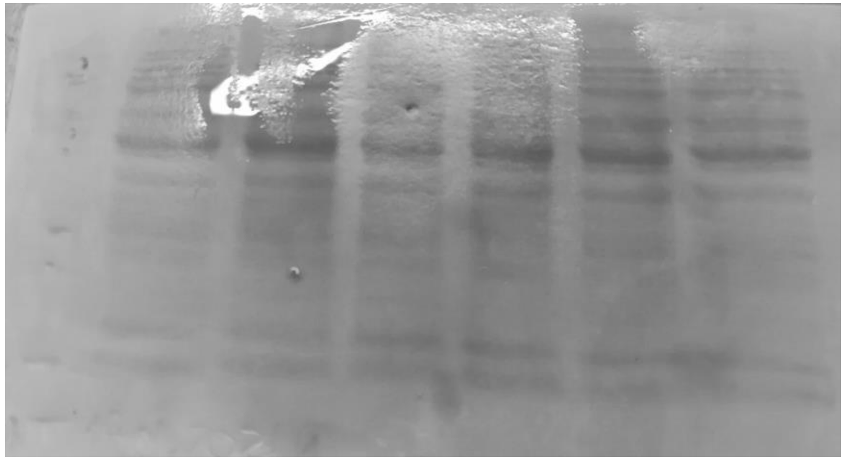

2

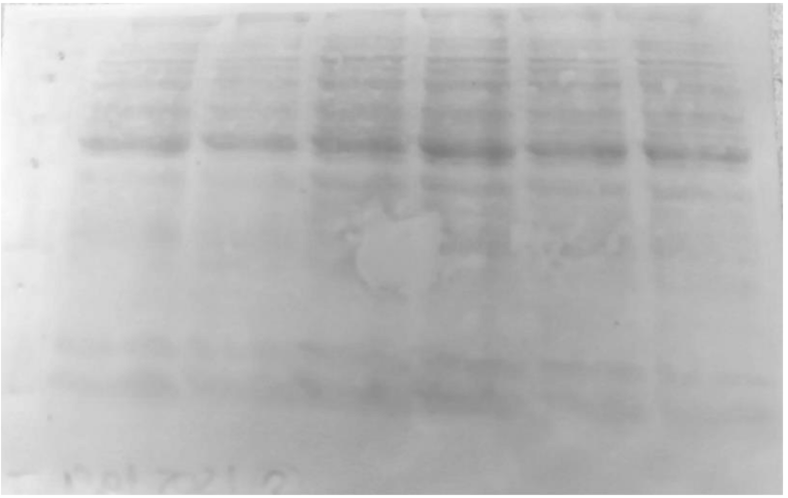

3

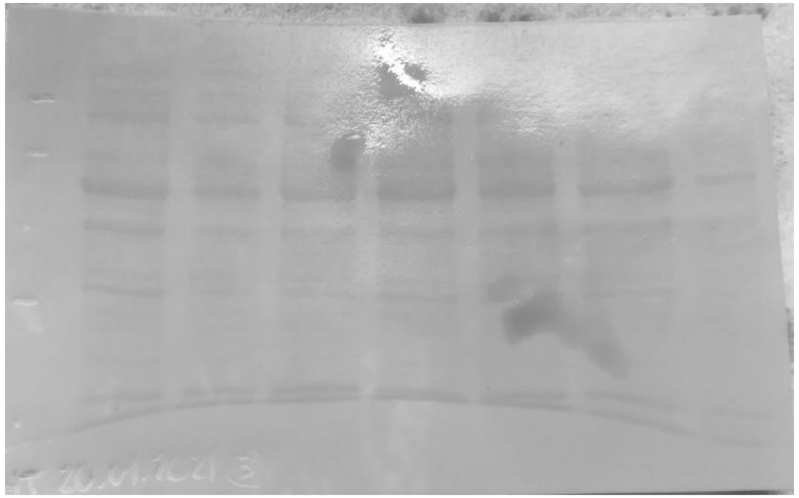

4

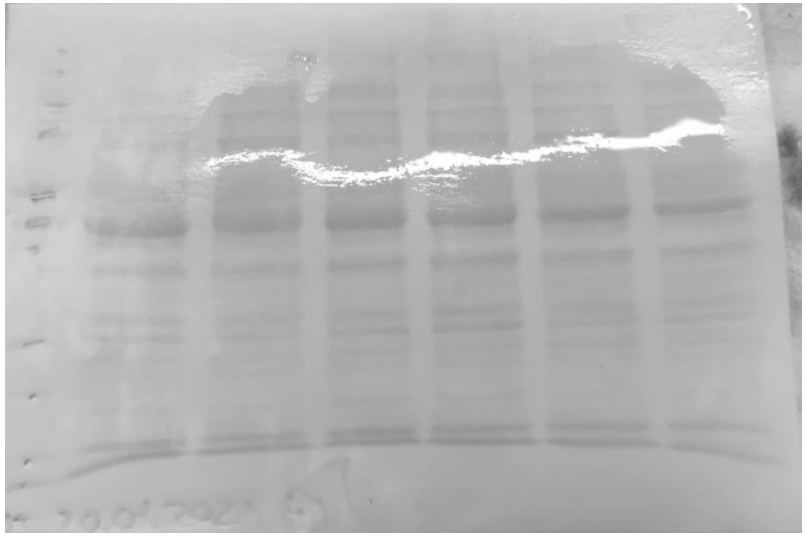

5

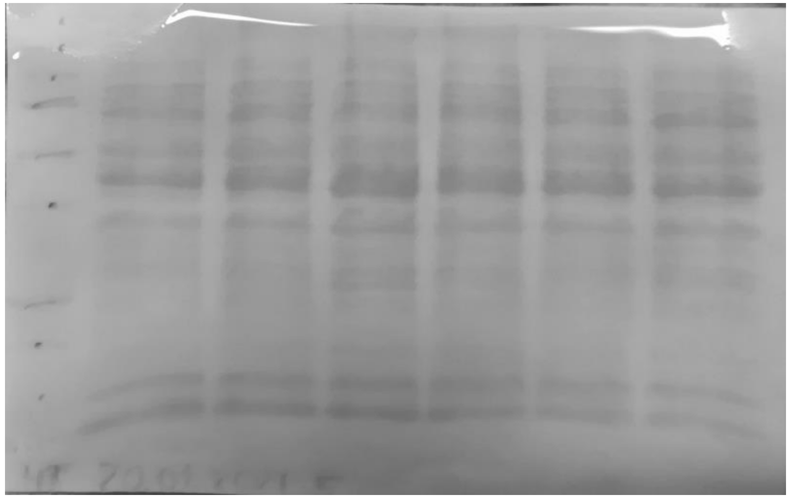

6

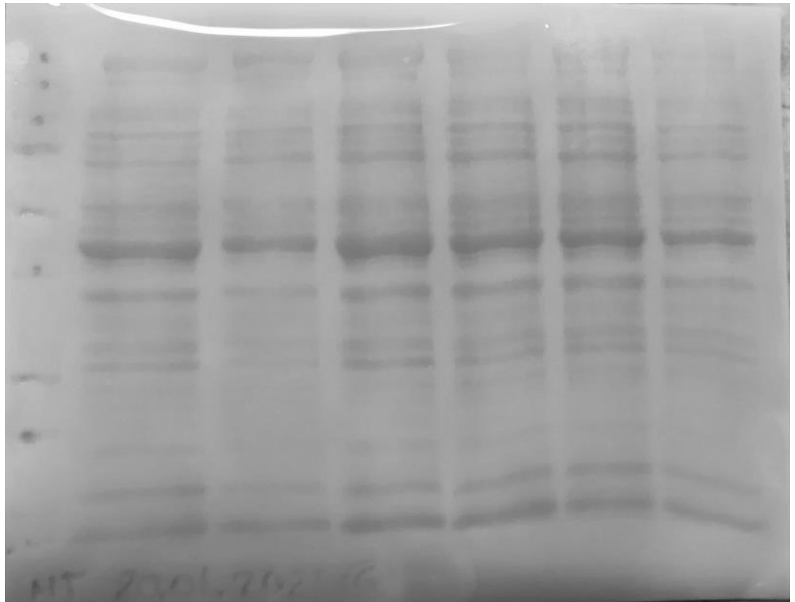

PFKM - Blot

This study

Samples order from the molecular weight marker: **MTX | DOX18 | CTRL** | DOX9-1 | CTRL-2 | DOX9-2

Other study

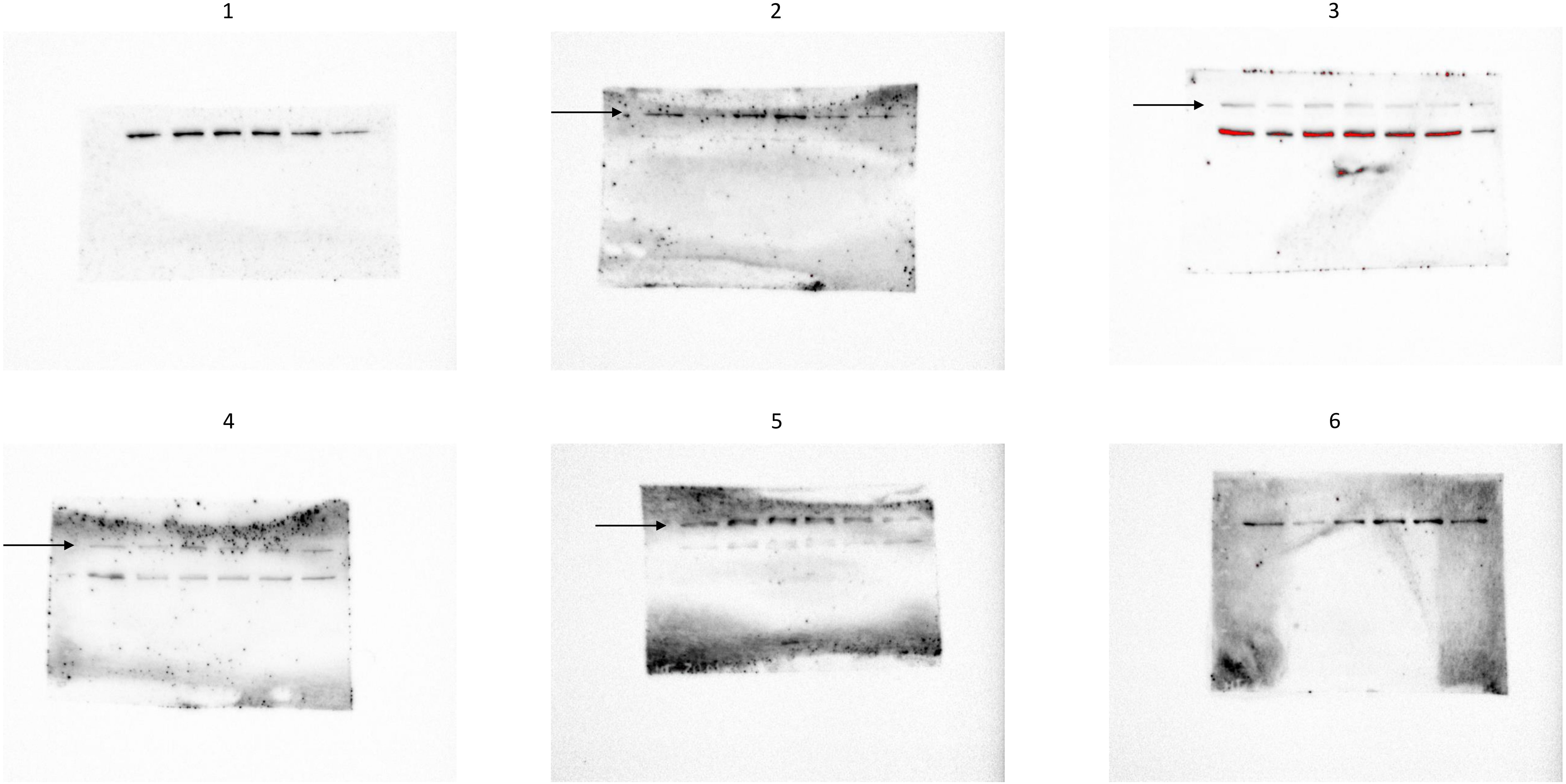

GAPDH - Ponceau S

This study

Samples order from the molecular weight marker: **MTX | DOX18 | CTRL** | DOX9-1 | CTRL-2 | DOX9-2

Other study

1

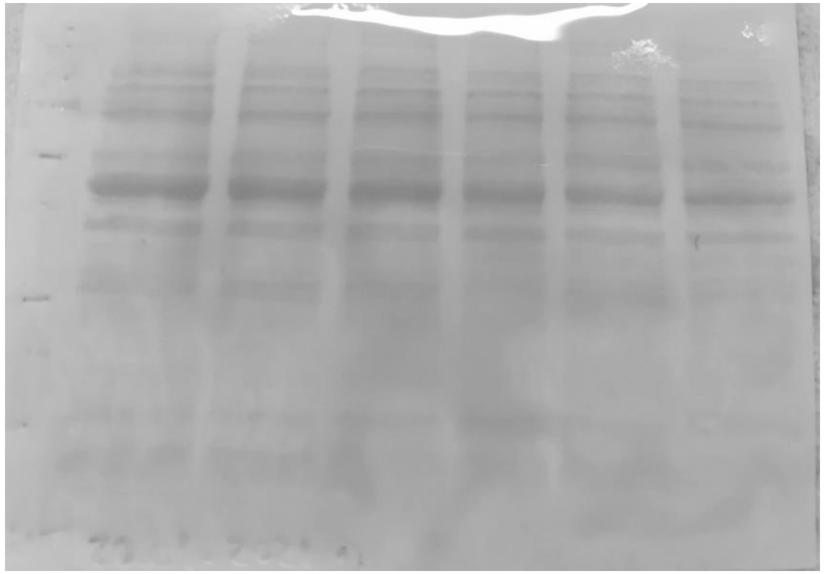

2

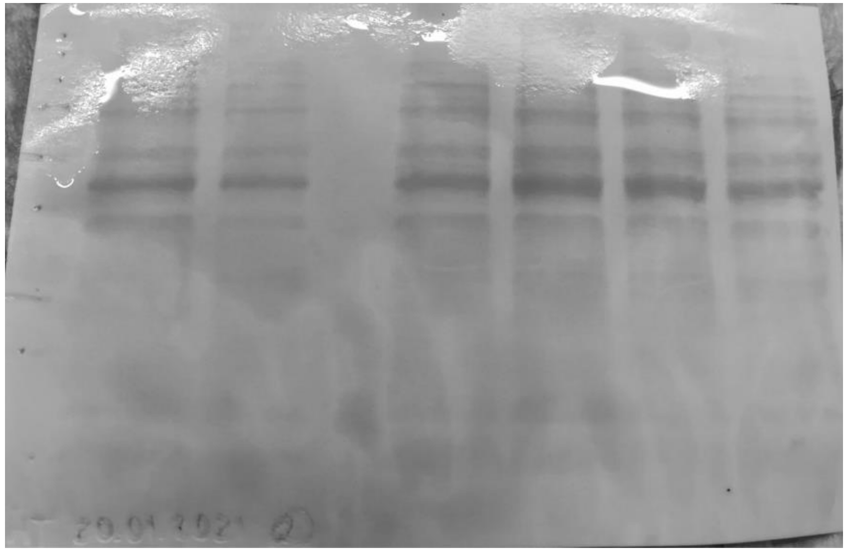

3

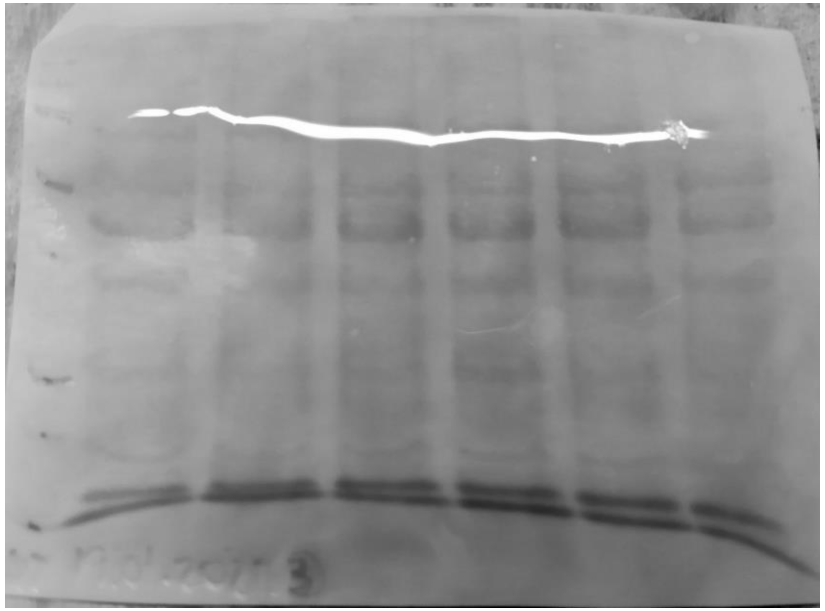

4

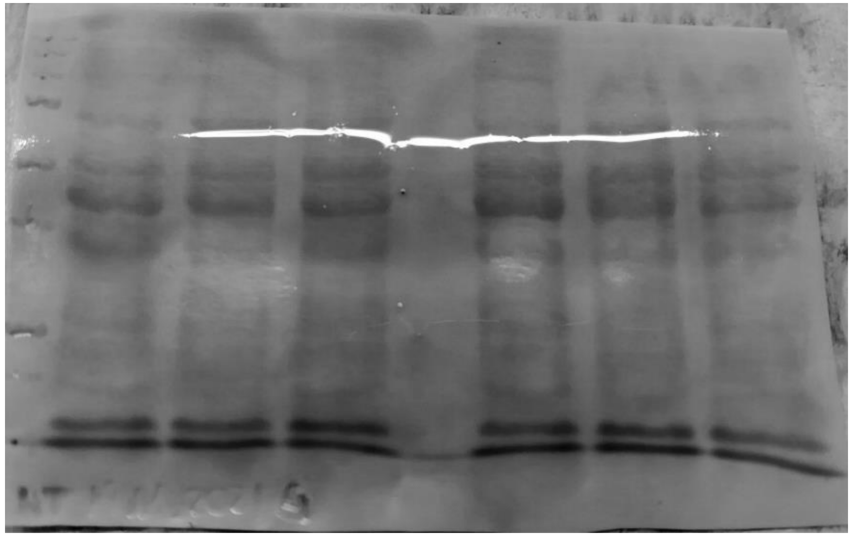

5

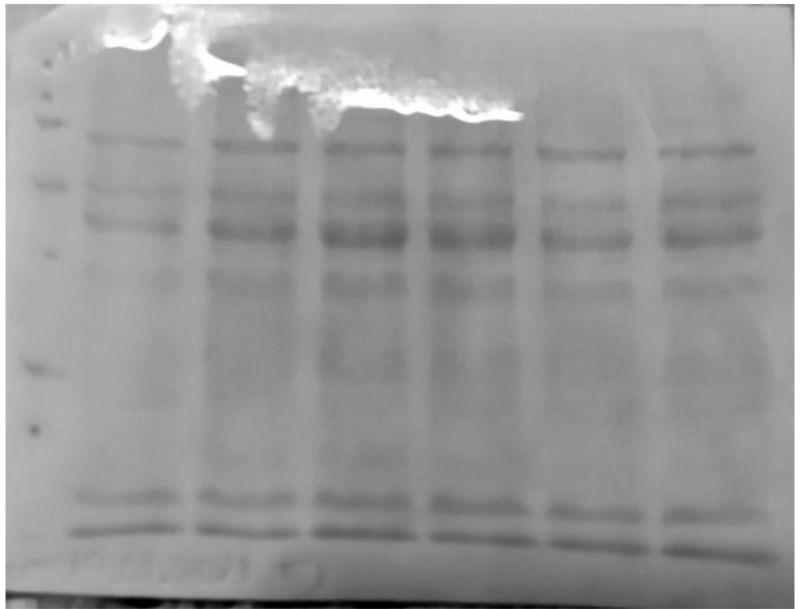

6

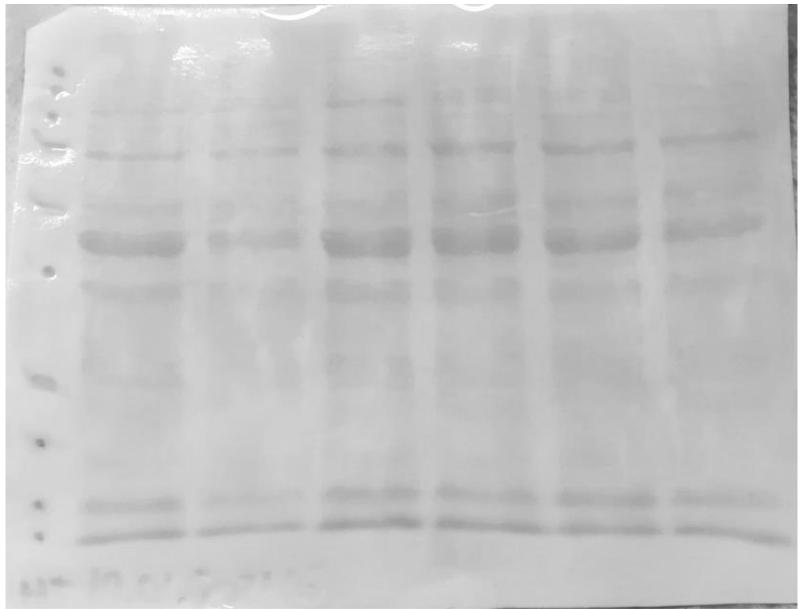

GAPDH - Blot

This study

Samples order from the molecular weight marker: **MTX | DOX18 | CTRL** | DOX9-1 | CTRL-2 | DOX9-2

Other study

1

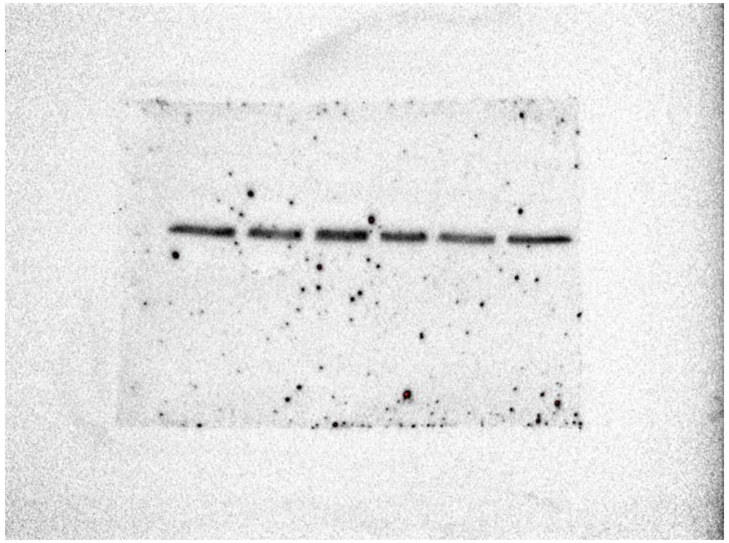

2

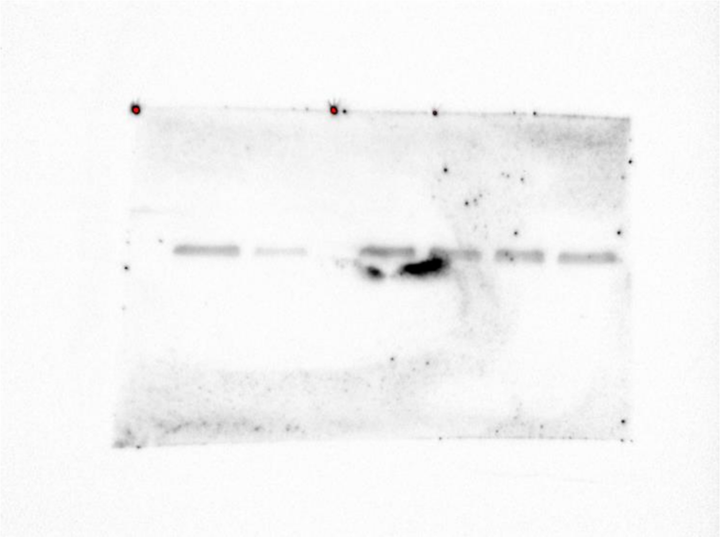

3

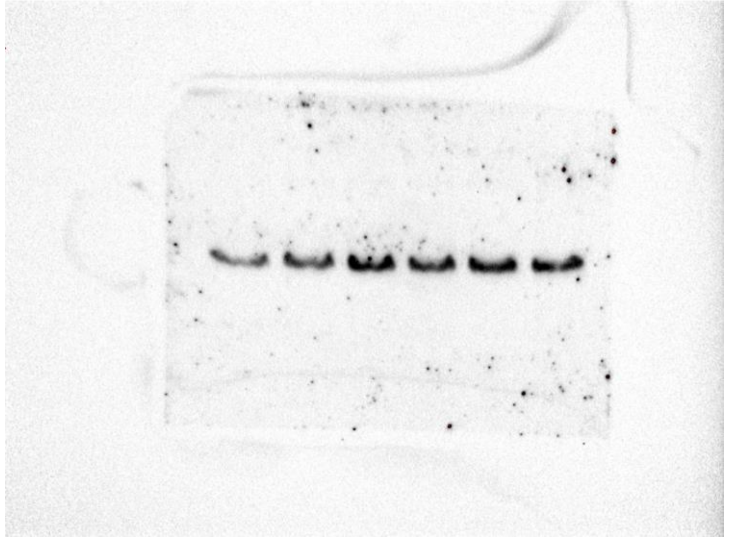

4

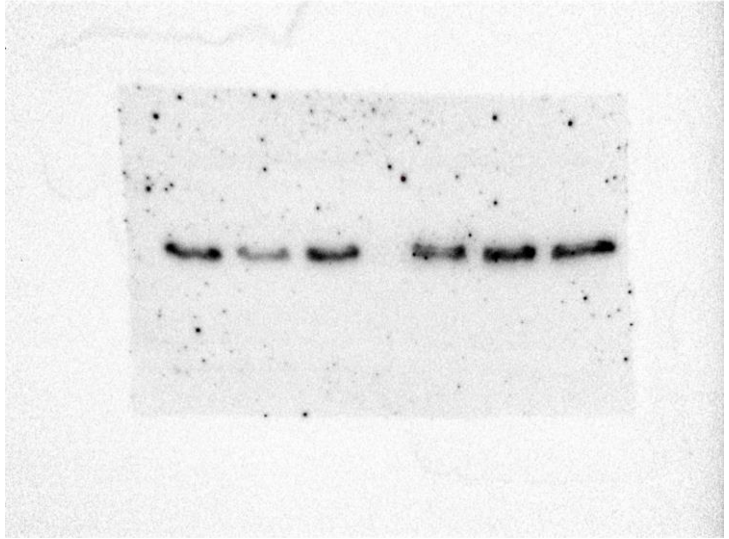

5

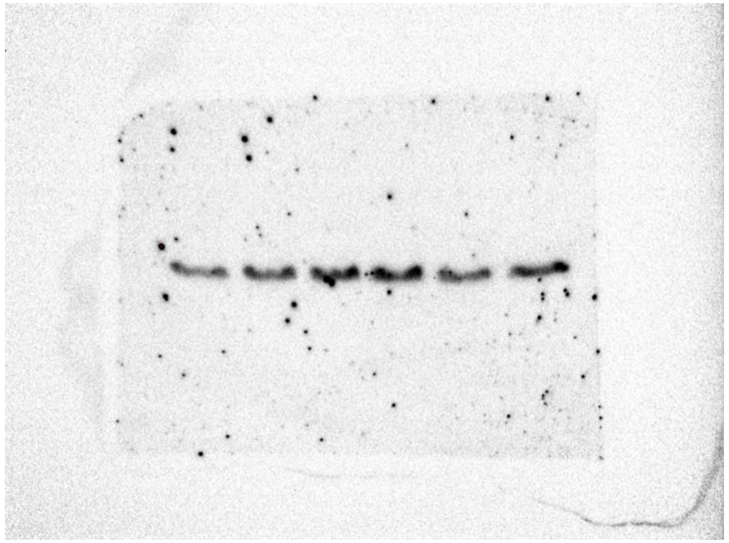

6

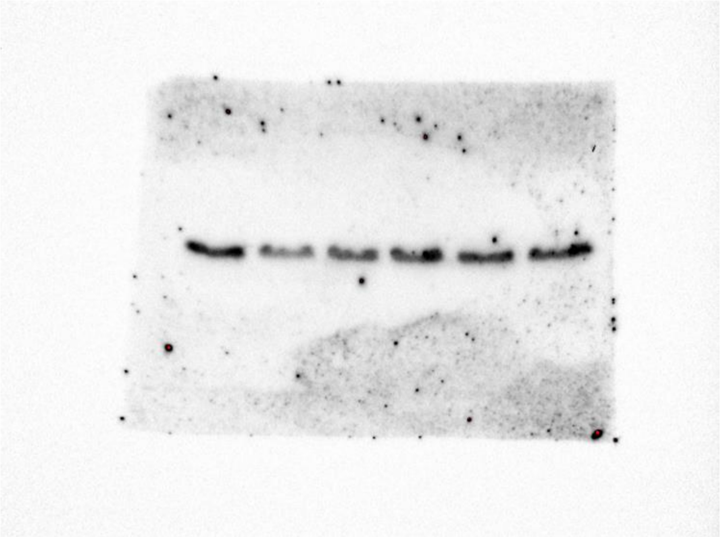

ETFDH - Ponceau S

This study

Samples order from the molecular weight marker: **MTX | DOX18 | CTRL** | DOX9-1 | CTRL-2 | DOX9-2

Other study

1

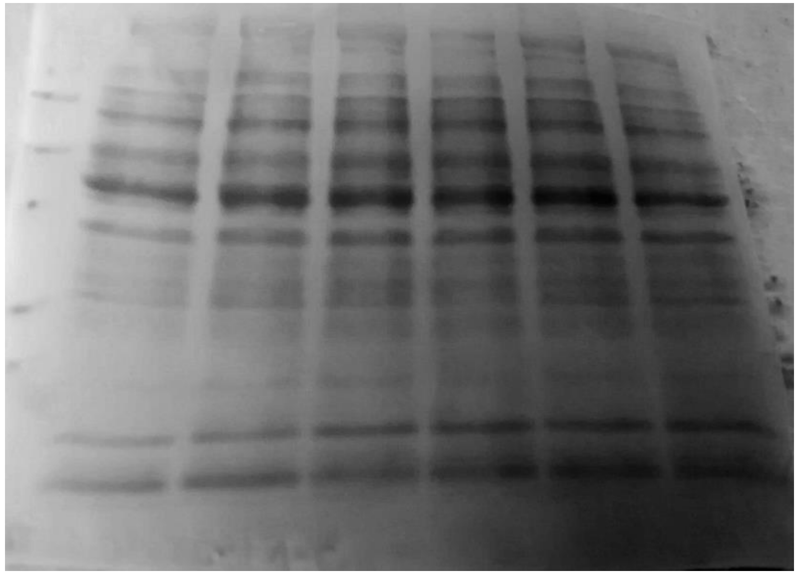

2

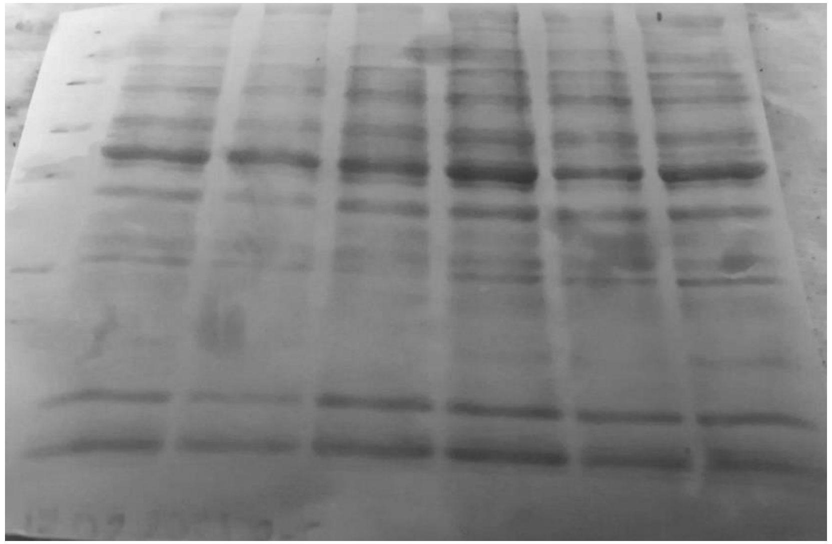

3

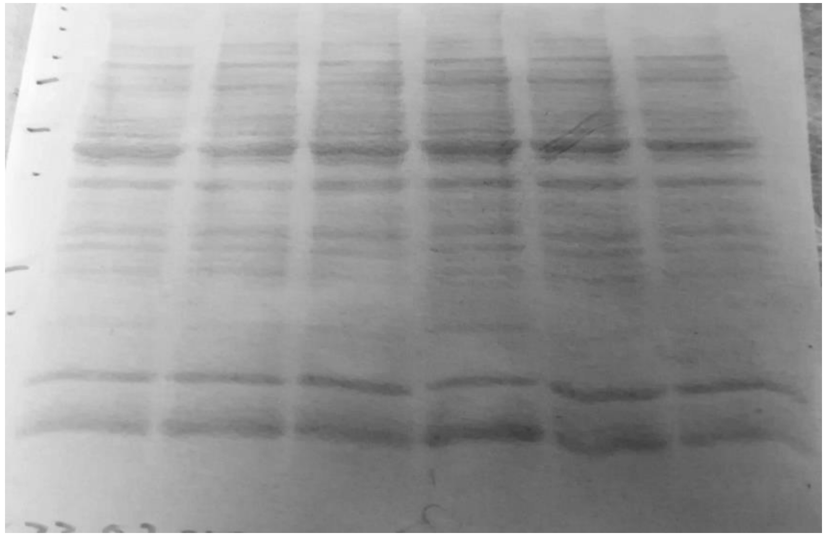

4

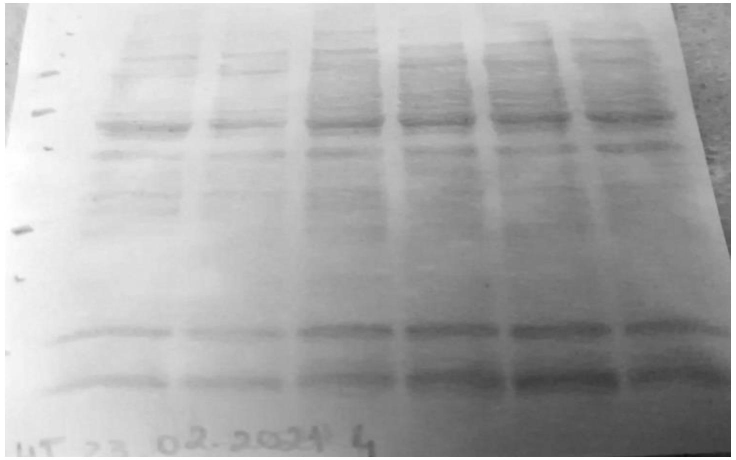

5

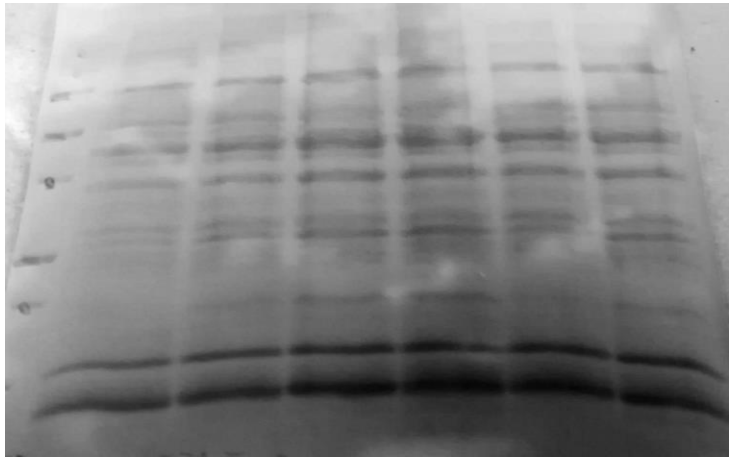

6

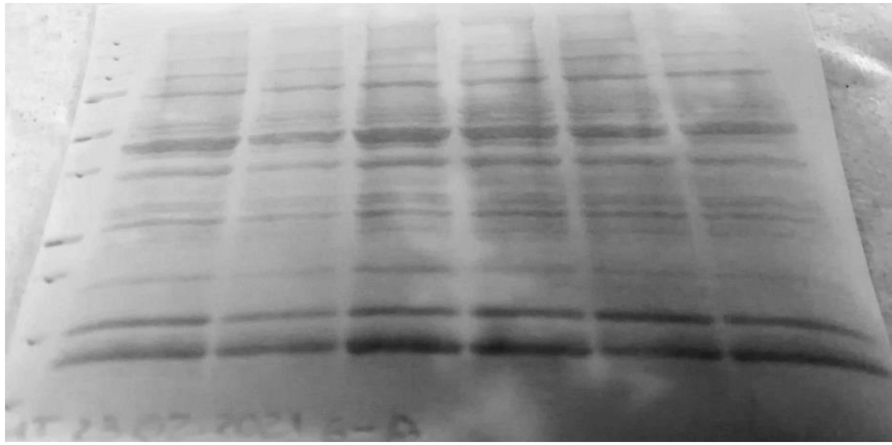

ETFDH - Blot

This study

Samples order from the molecular weight marker: **MTX | DOX18 | CTRL** | DOX9-1 | CTRL-2 | DOX9-2

Other study

1

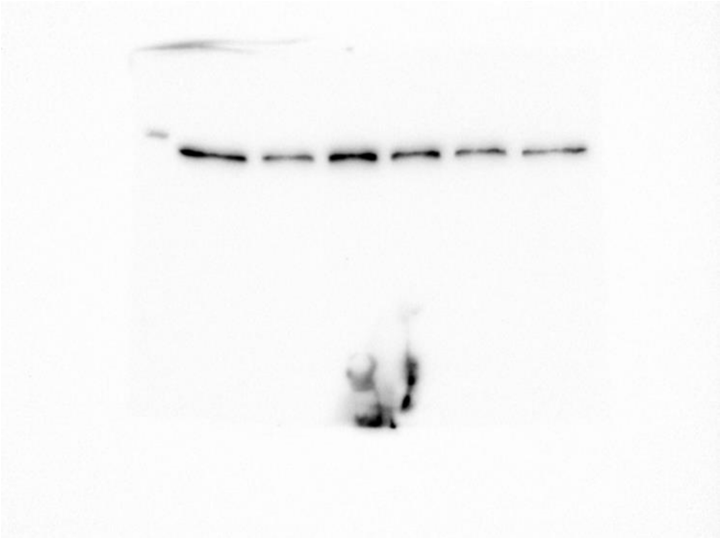

2

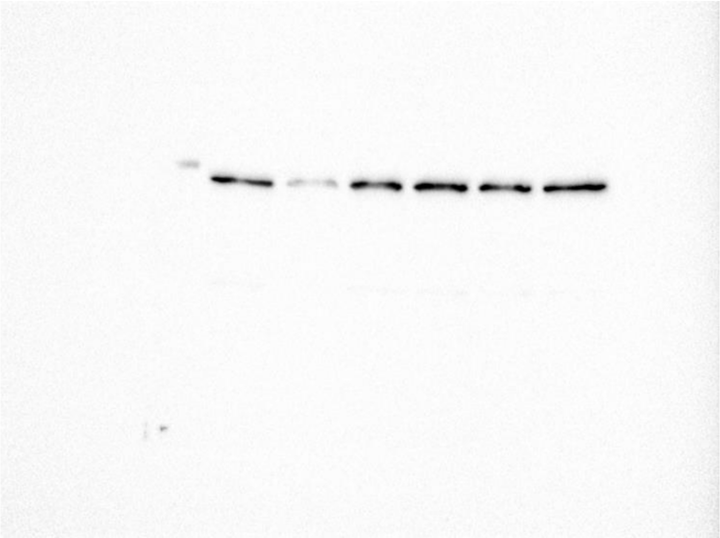

3

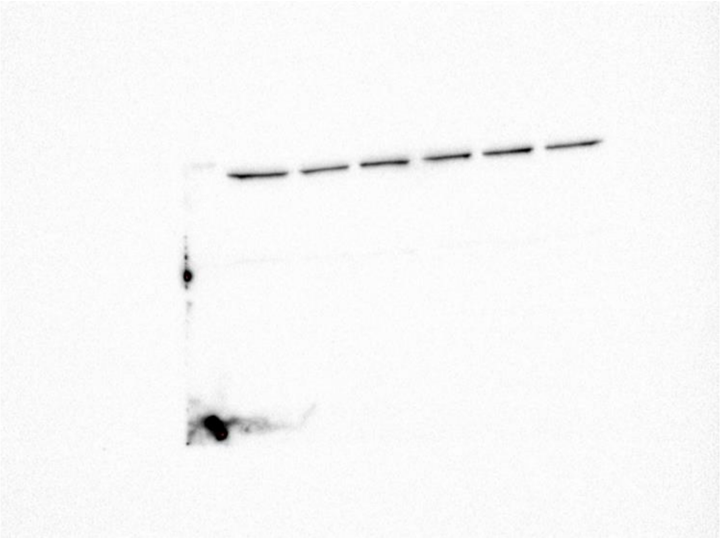

4

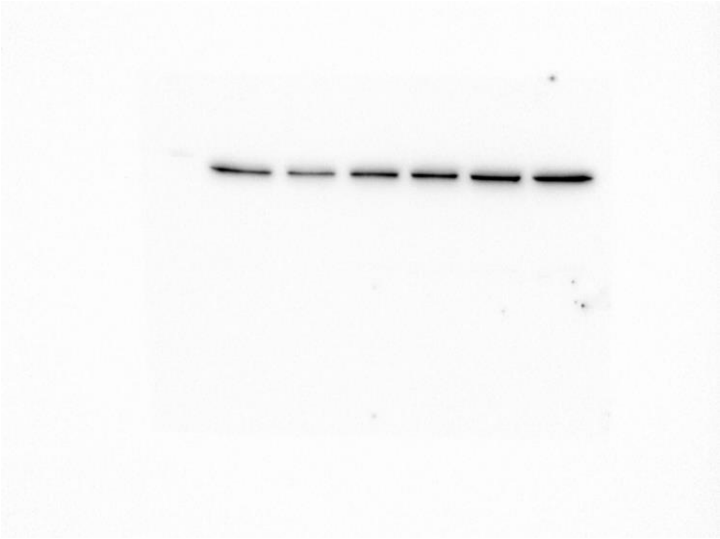

5

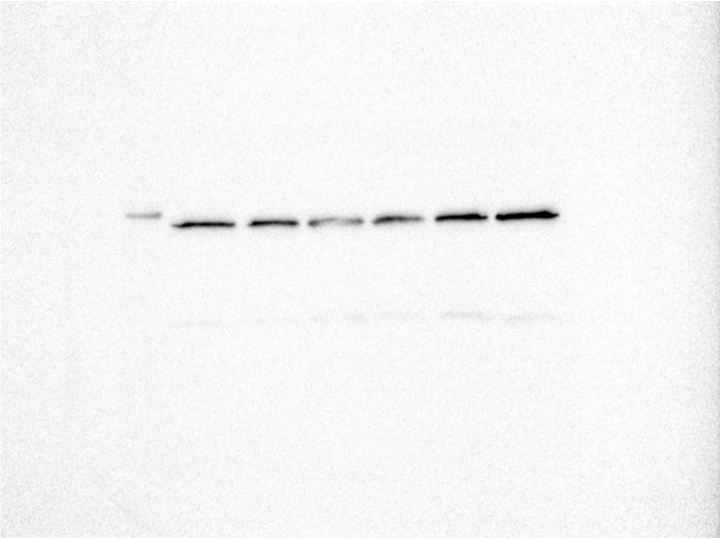

6

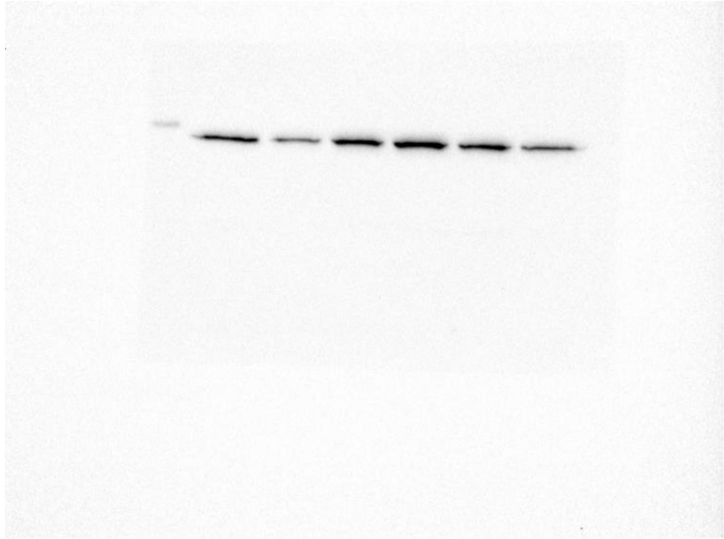

ATPB - Ponceau S

This study

Samples order from the molecular weight marker: **MTX | DOX18 | CTRL** | DOX9-1 | CTRL-2 | DOX9-2

Other study

1

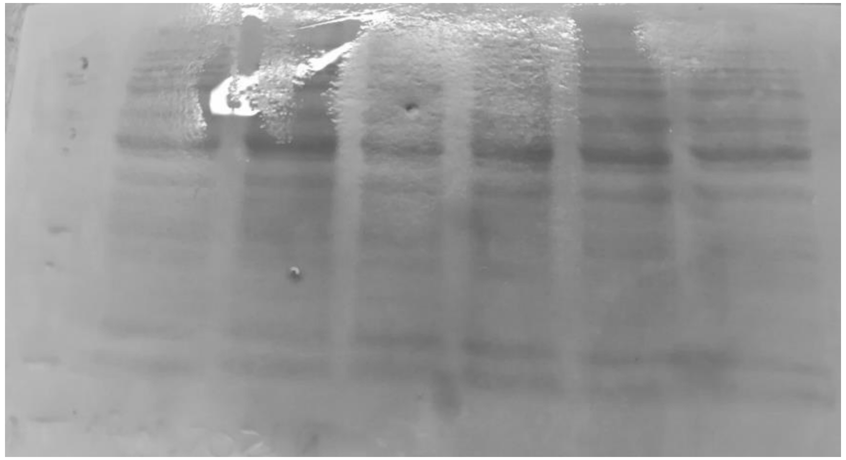

2

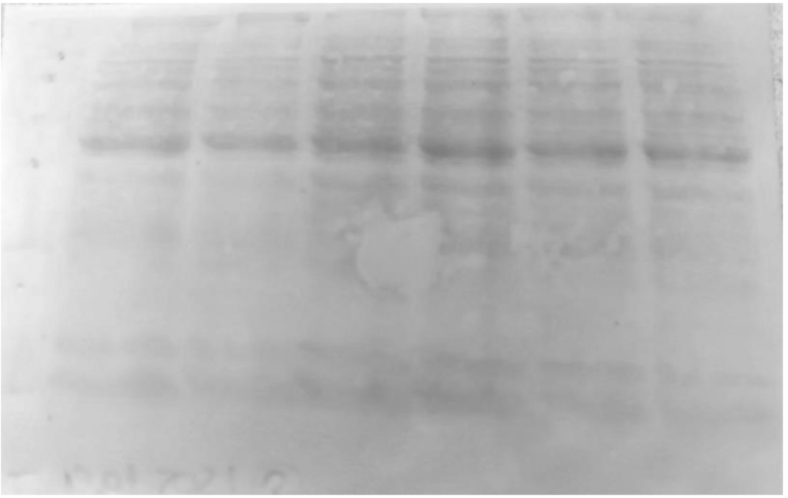

3

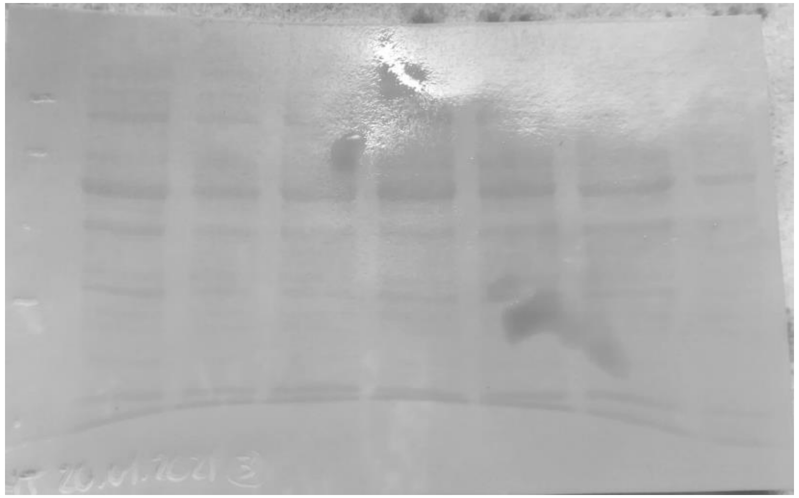

4

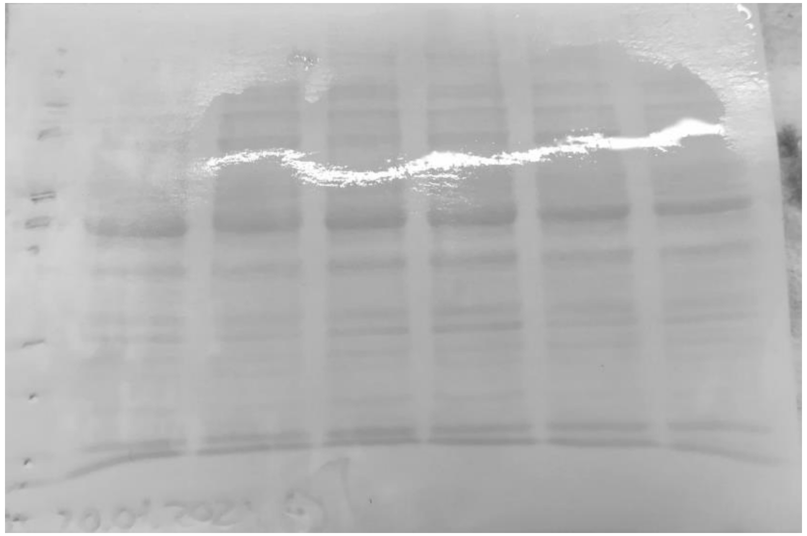

5

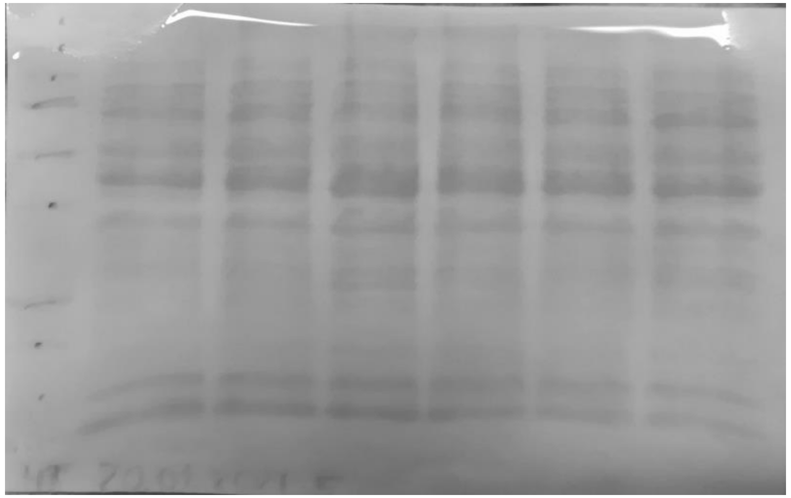

6

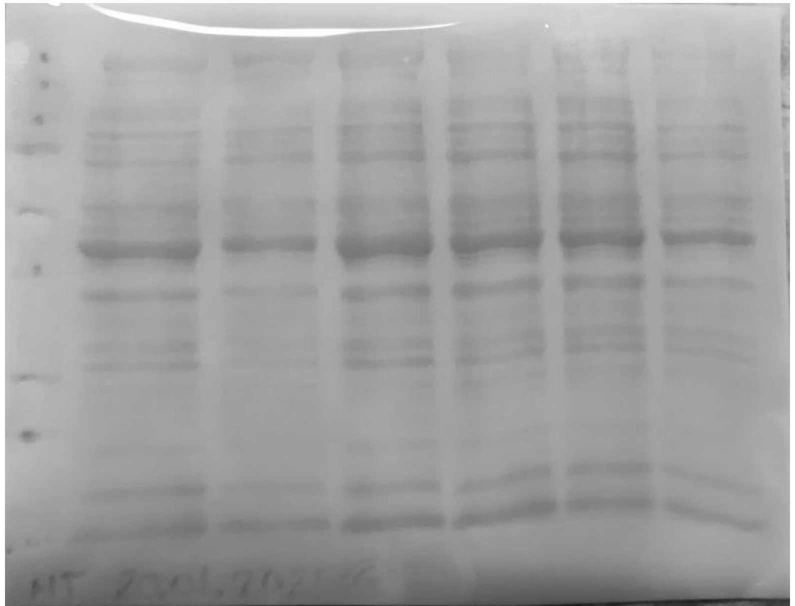

ATPB - Blot

This study

Samples order from the molecular weight marker: **MTX | DOX18 | CTRL** | DOX9-1 | CTRL-2 | DOX9-2

Other study

1

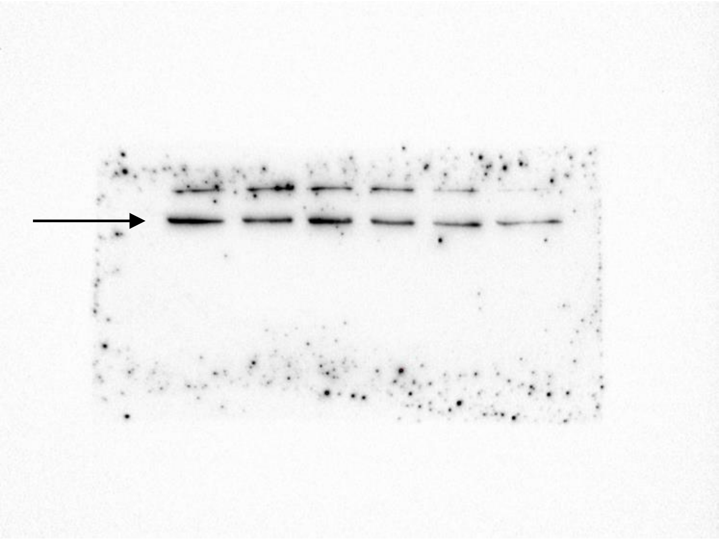

2

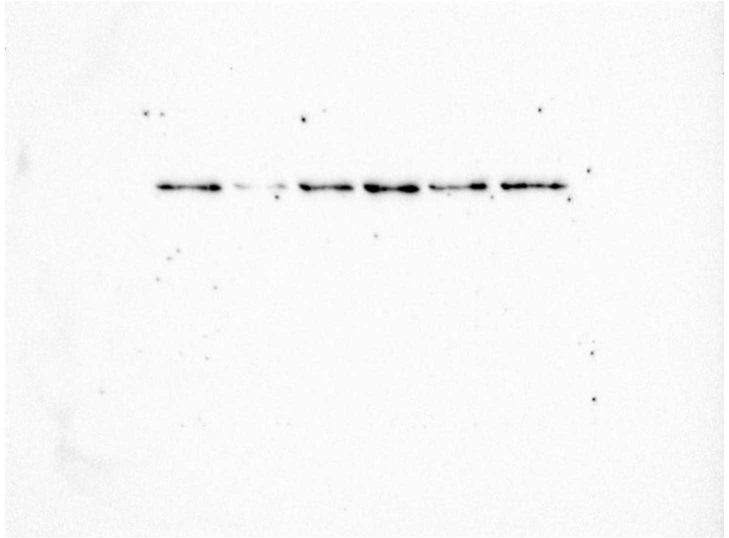

3

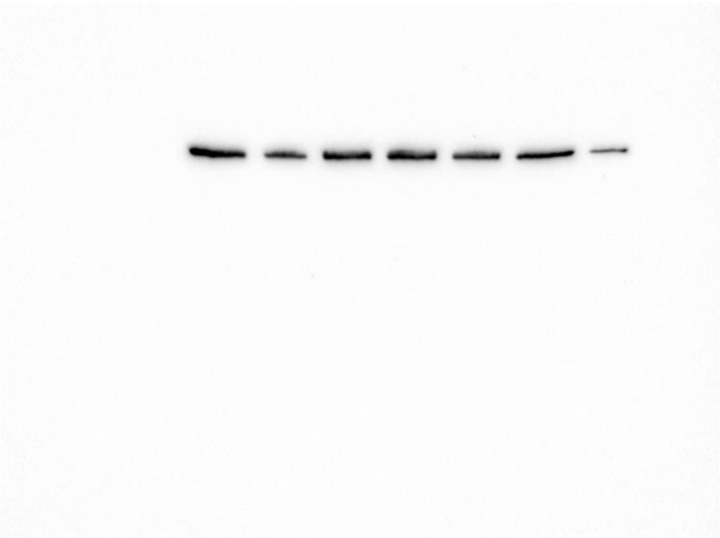

4

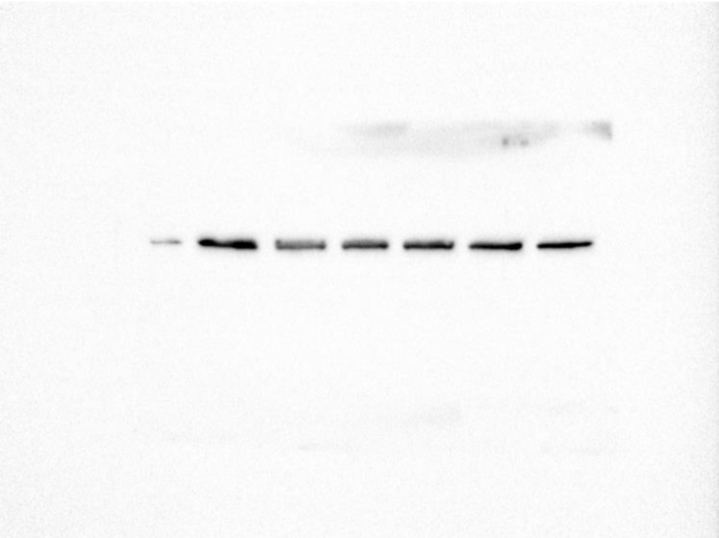

5

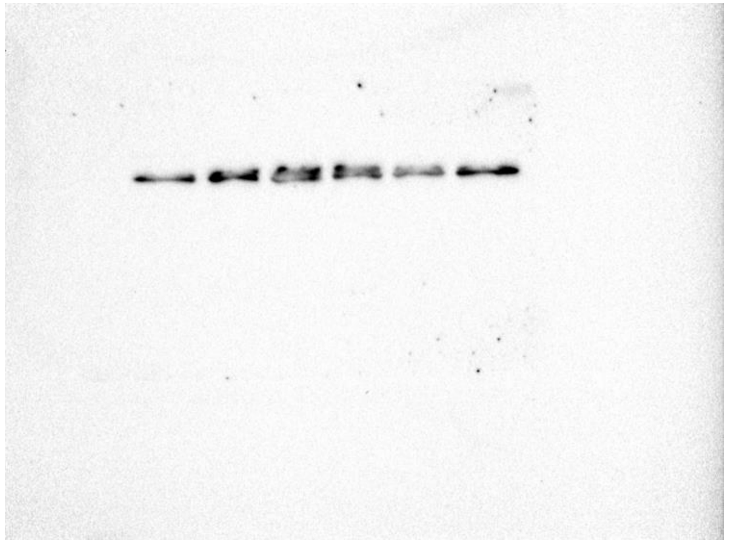

6

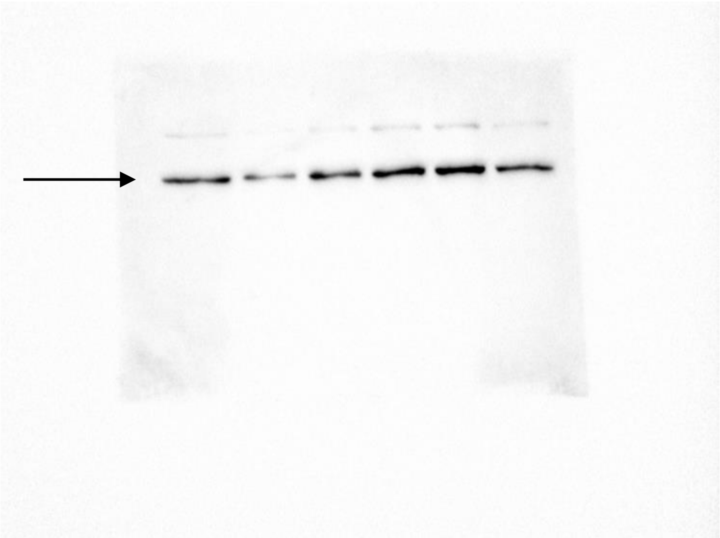

AMPK - Ponceau S

This study

Samples order from the molecular weight marker: **MTX | DOX18 | CTRL** | DOX9-1 | CTRL-2 | DOX9-2

Other study

1

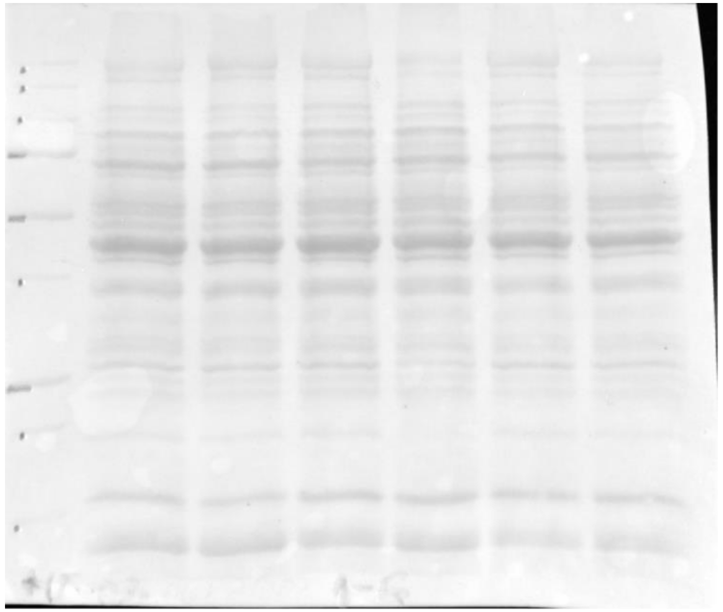

2

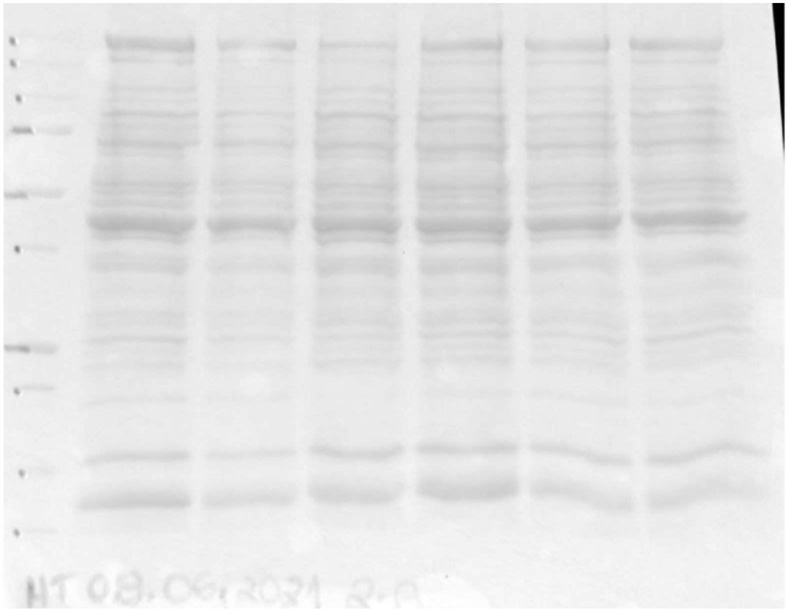

3

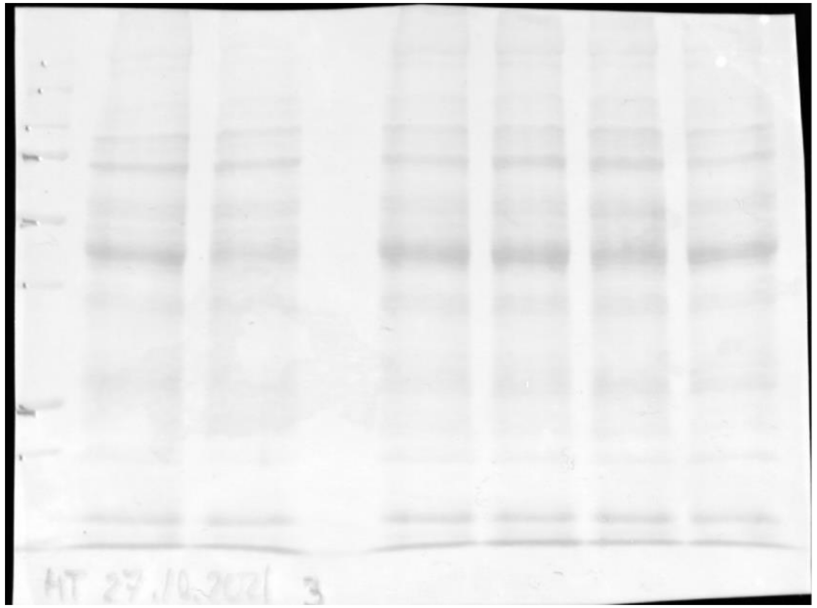

4

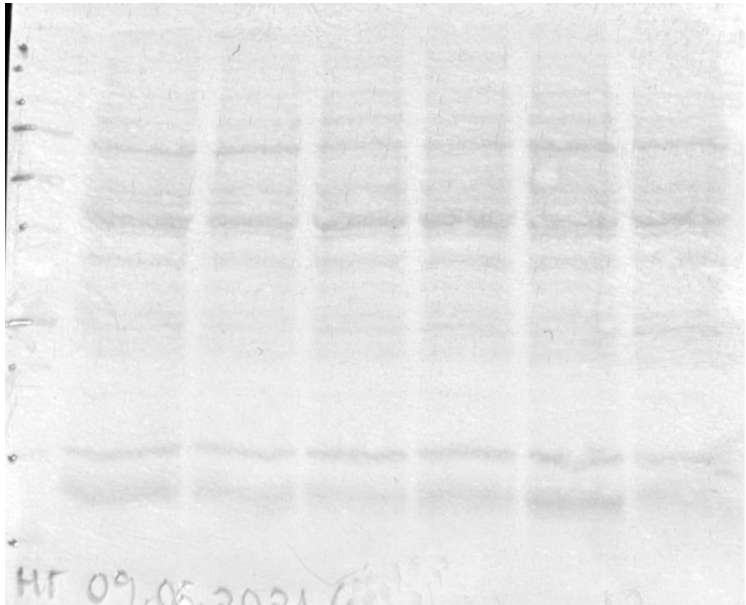

5

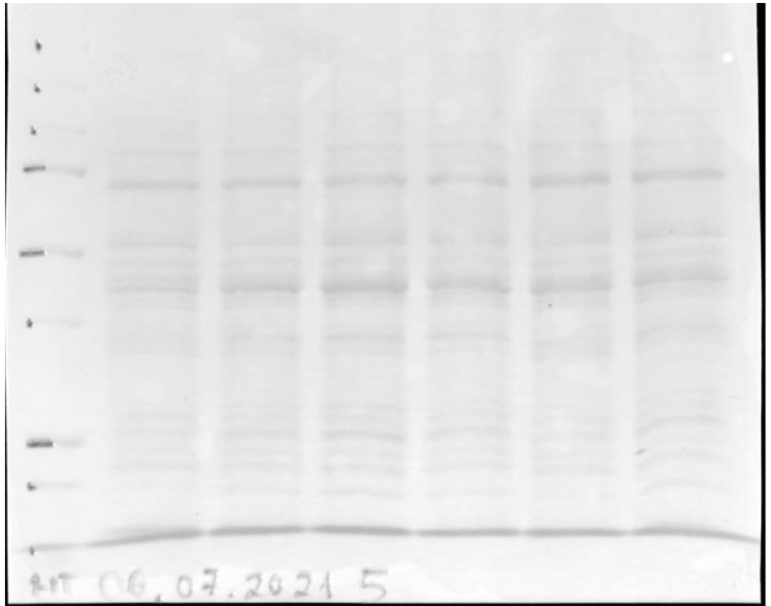

6

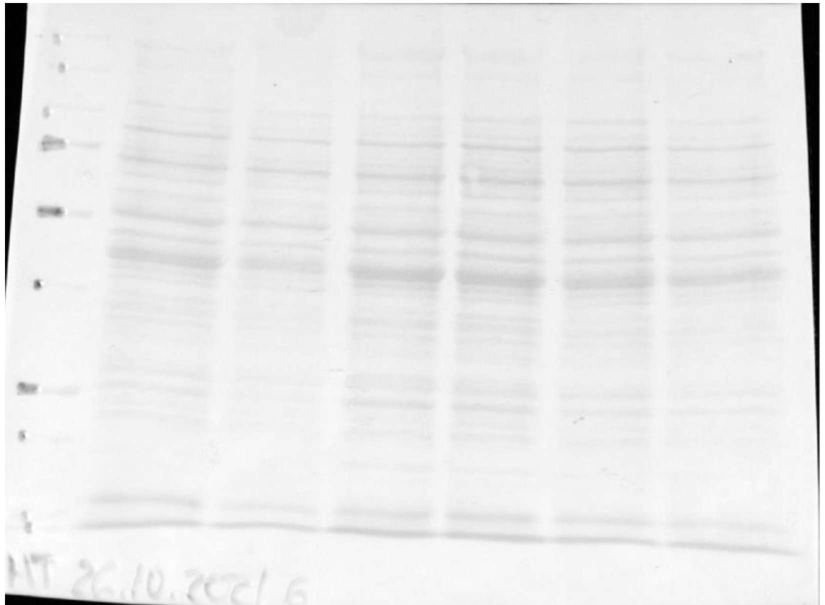

AMPK - Blot

This study

Samples order from the molecular weight marker: **MTX | DOX18 | CTRL** | DOX9-1 | CTRL-2 | DOX9-2

Other study

1

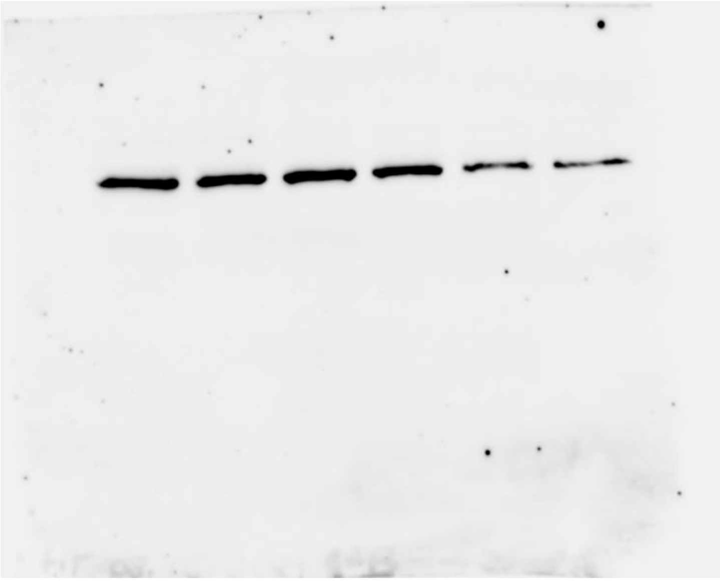

2

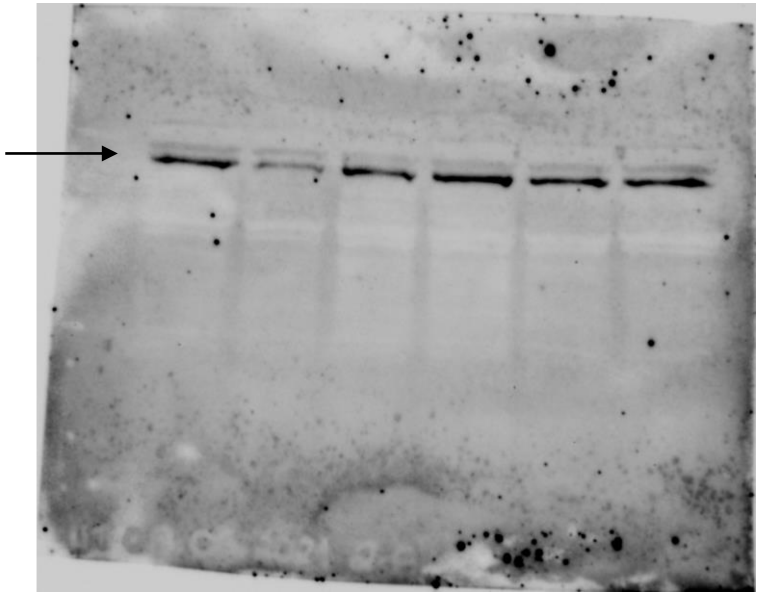

3

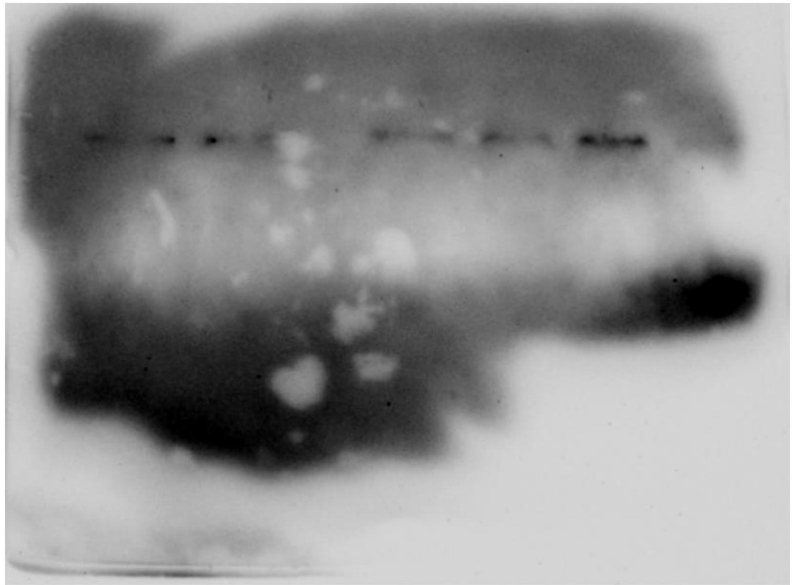

4

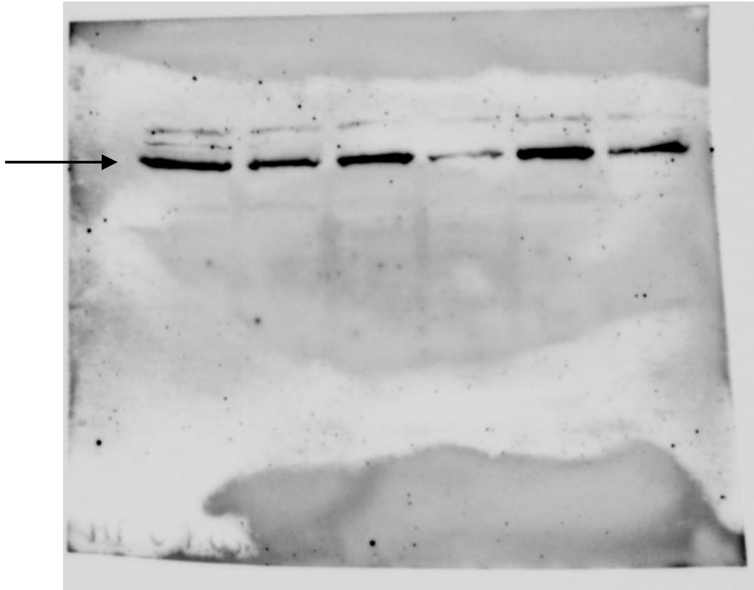

5

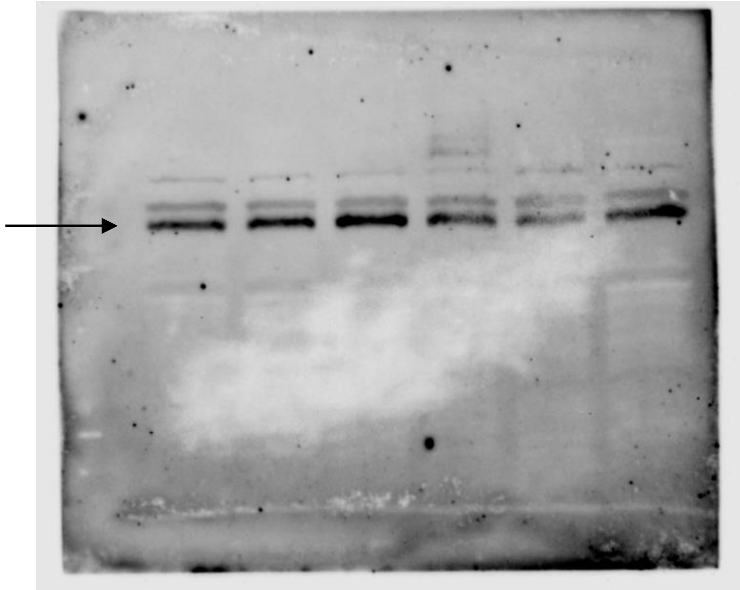

6

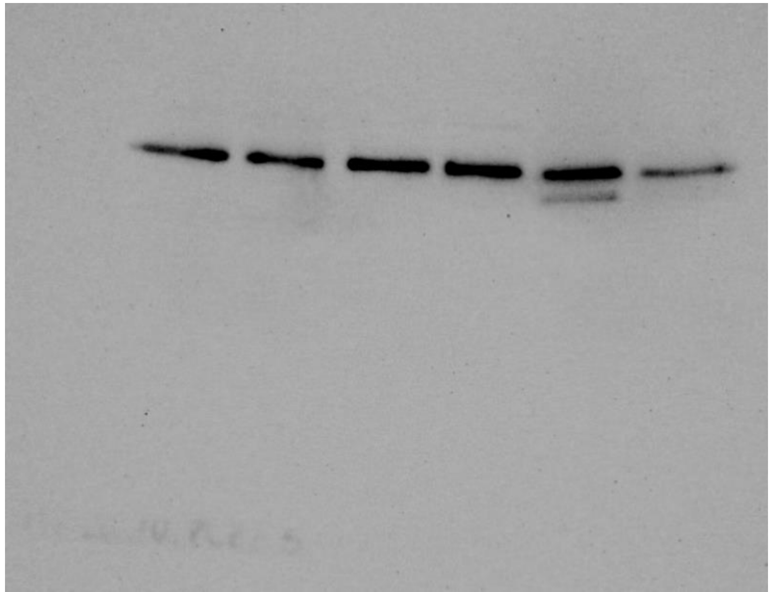

pAMPK - Ponceau S

This study

Samples order from the molecular weight marker: **MTX | DOX18 | CTRL** | DOX9-1 | CTRL-2 | DOX9-2

Other study

1

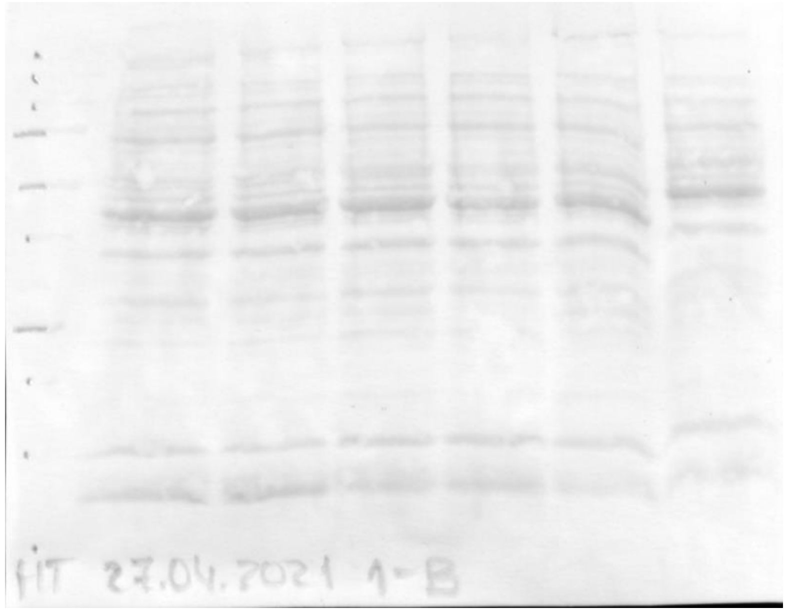

2

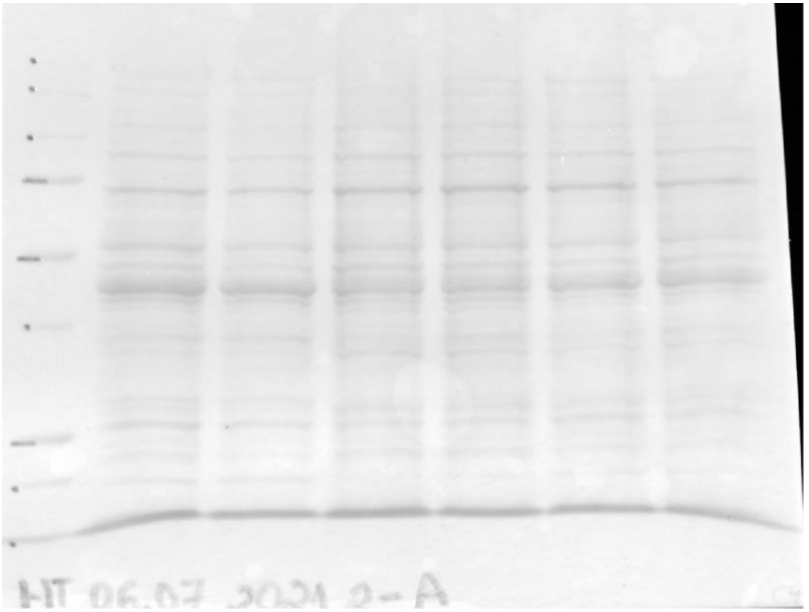

3

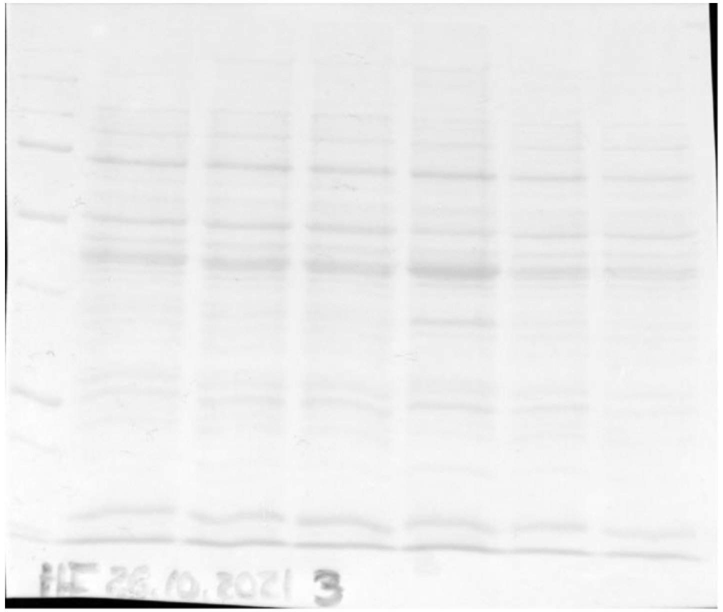

4

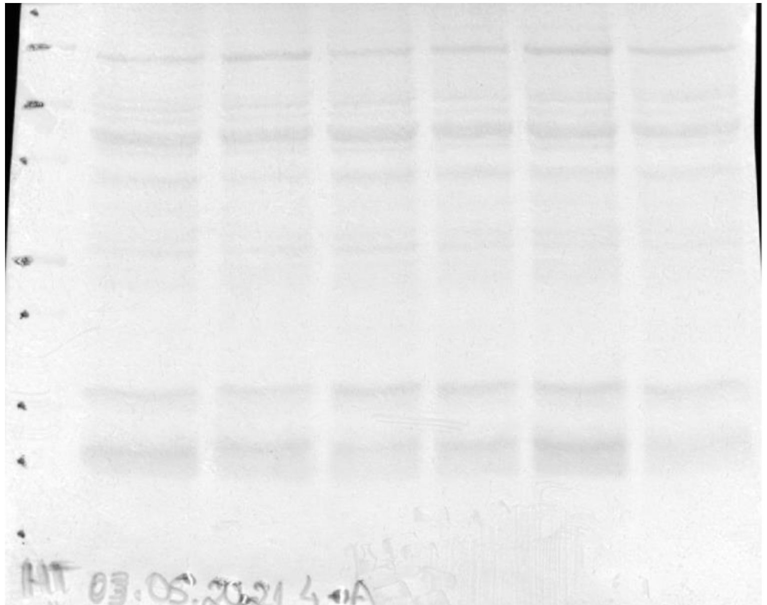

5

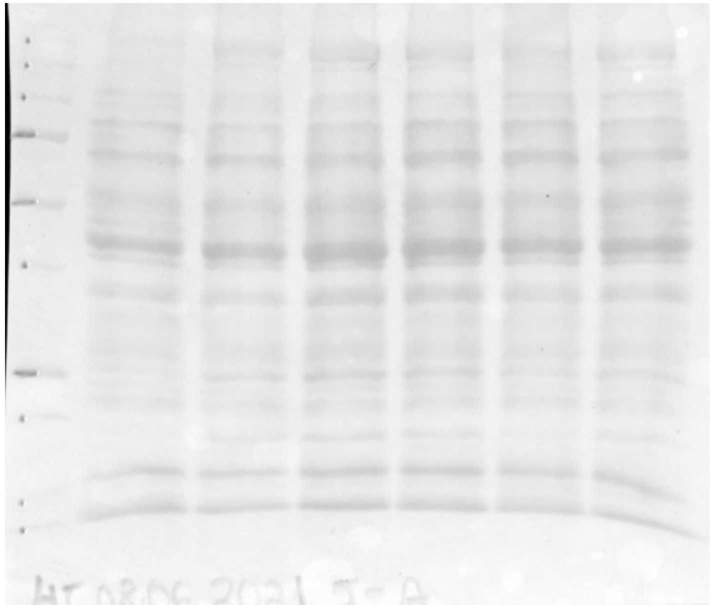

6

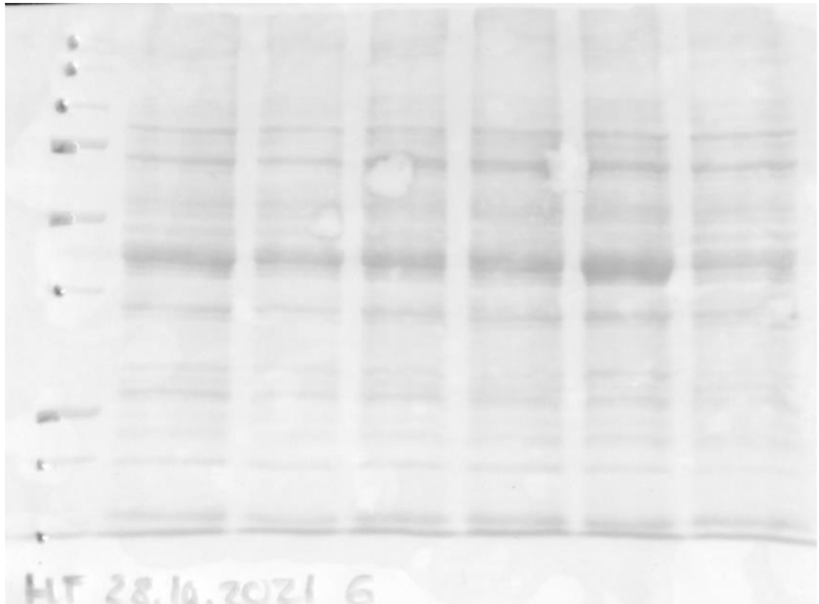

pAMPK - Blot

This study

Samples order from the molecular weight marker: **MTX | DOX18 | CTRL** | DOX9-1 | CTRL-2 | DOX9-2

Other study

1

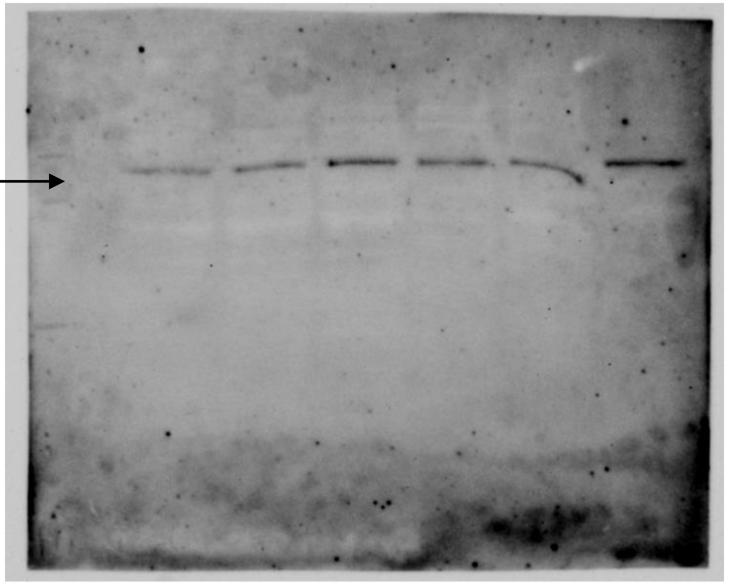

2

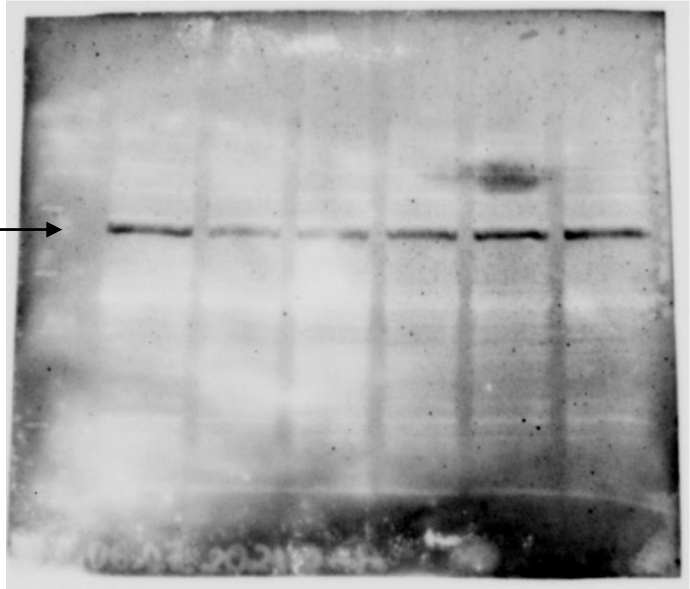

3

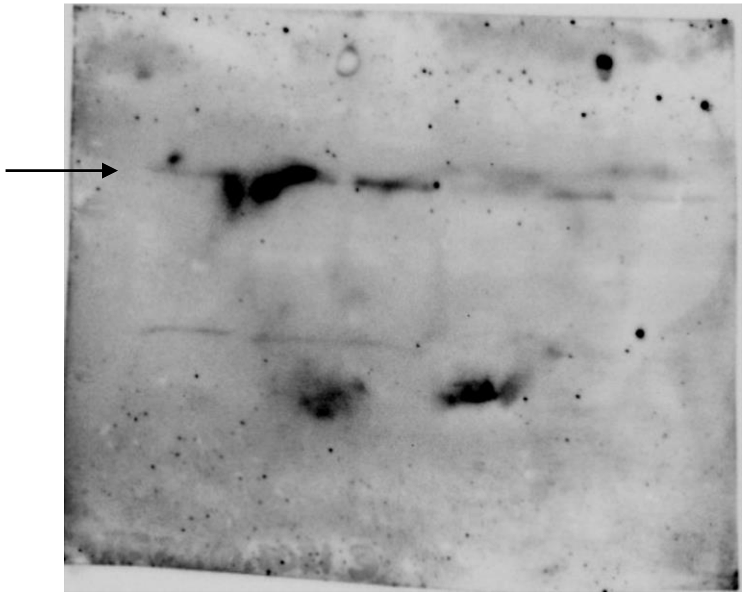

4

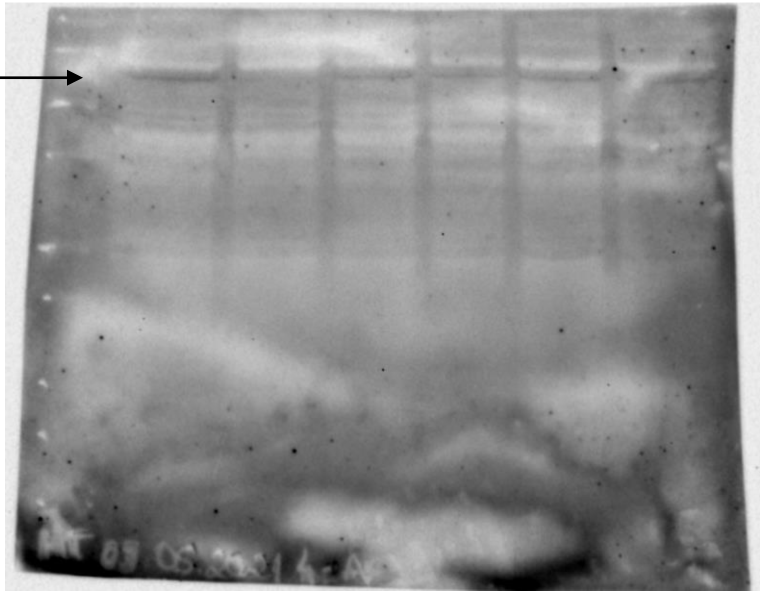

5

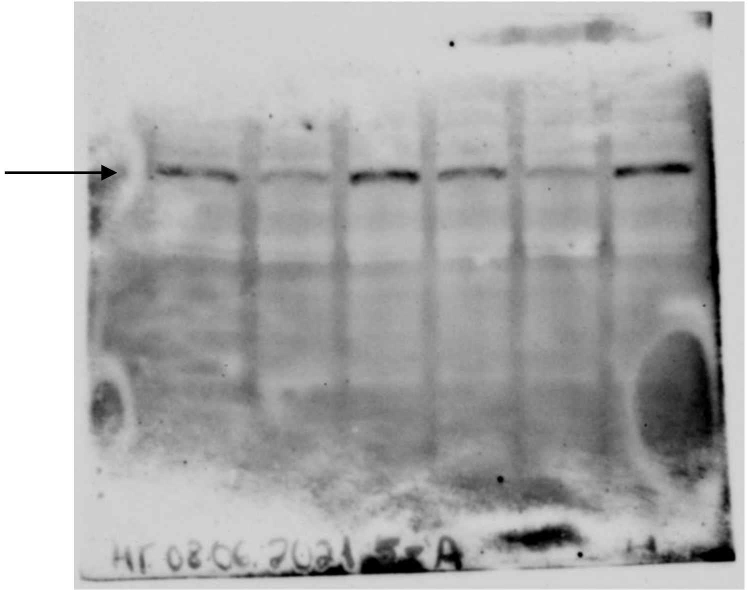

6

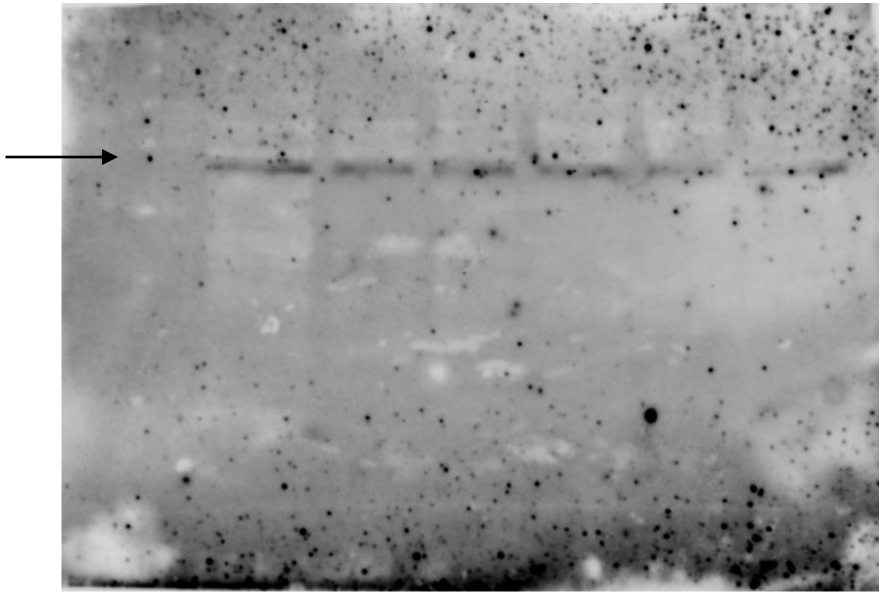

PPARα - Ponceau S

This study

Samples order from the molecular weight marker: **MTX | DOX18 | CTRL** | DOX9-1 | CTRL-2 | DOX9-2

Other study

1

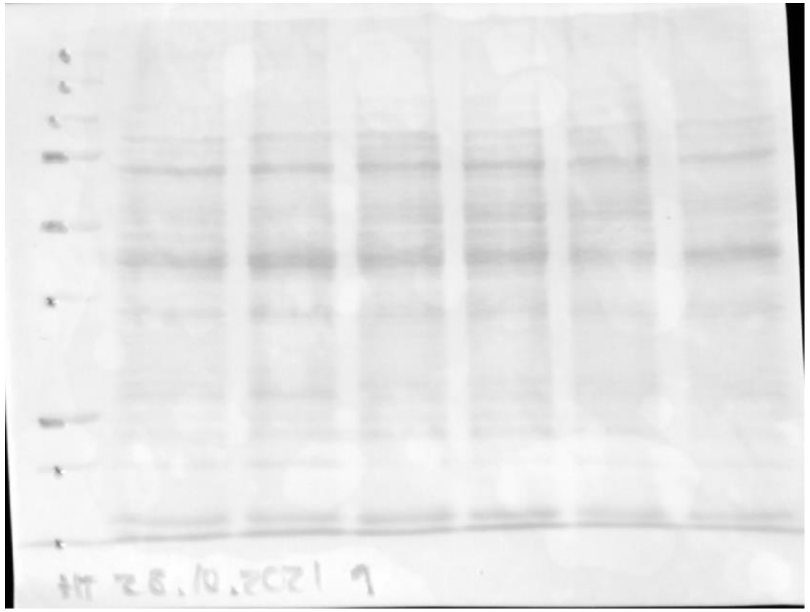

2

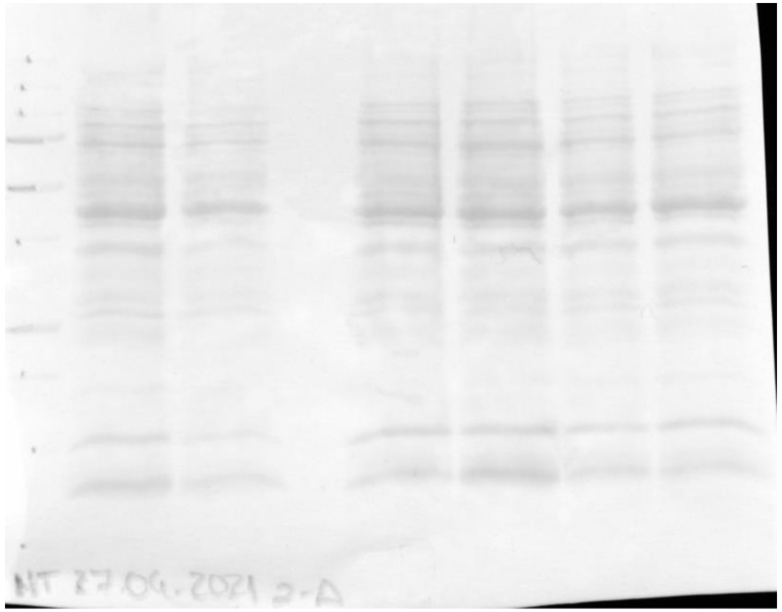

3

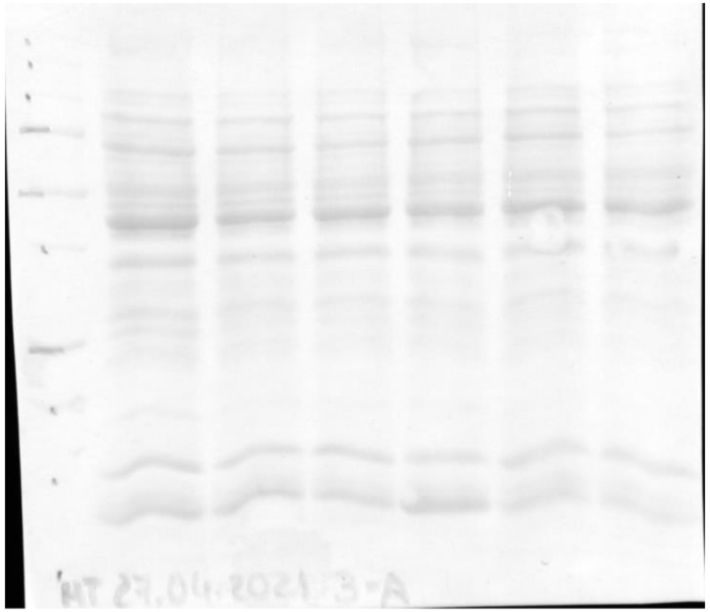

4

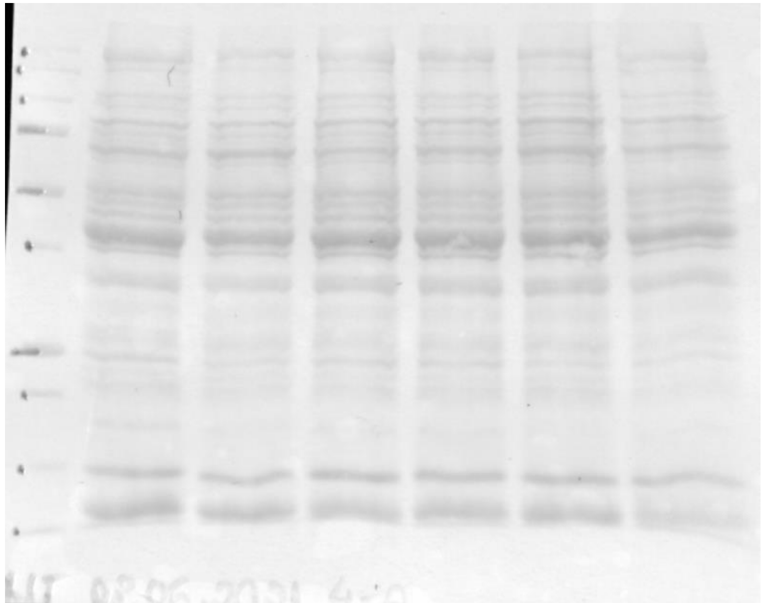

5

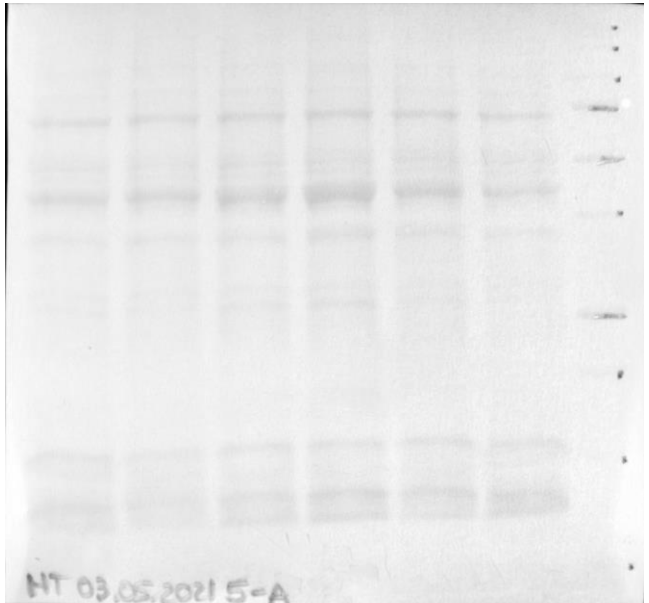

6

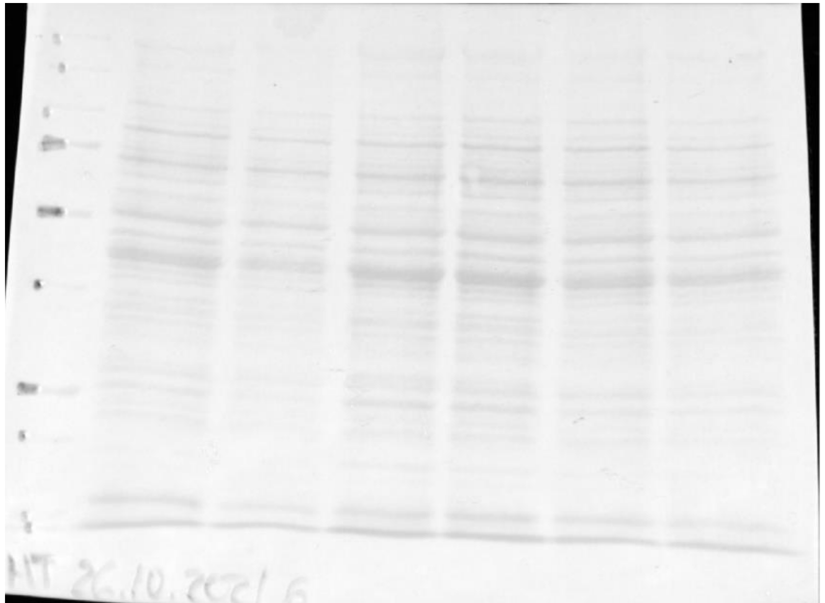

PPARα - Blot

This study

Samples order from the molecular weight marker: **MTX | DOX18 | CTRL** | DOX9-1 | CTRL-2 | DOX9-2

Other study

1

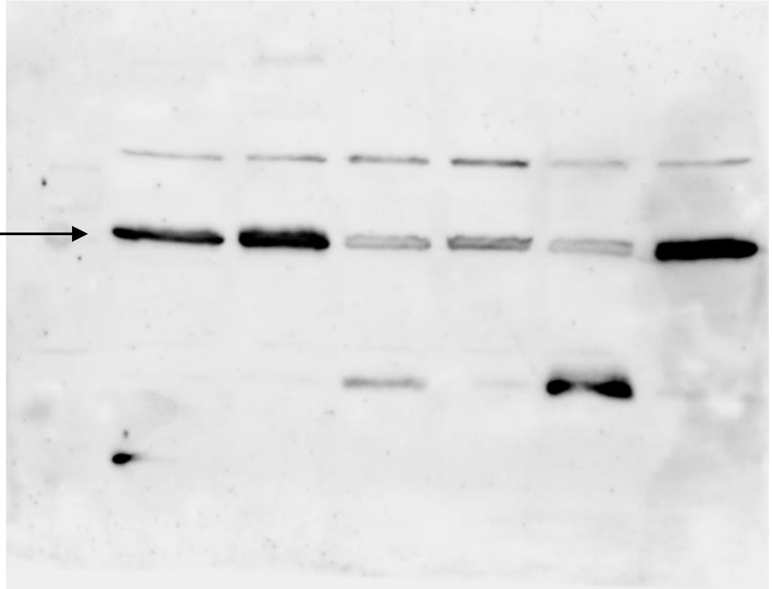

2

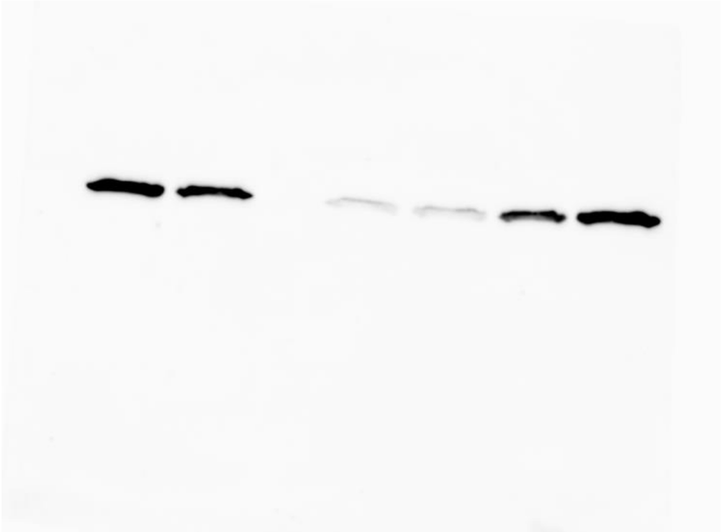

3

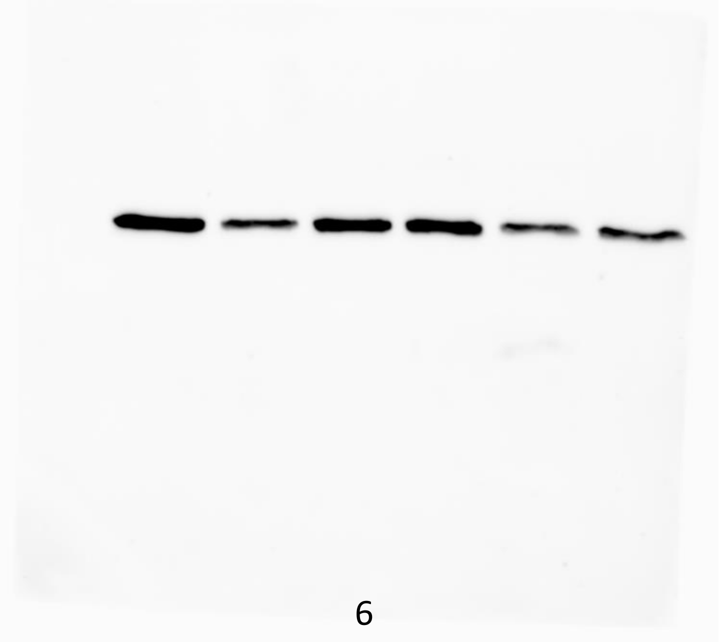

4

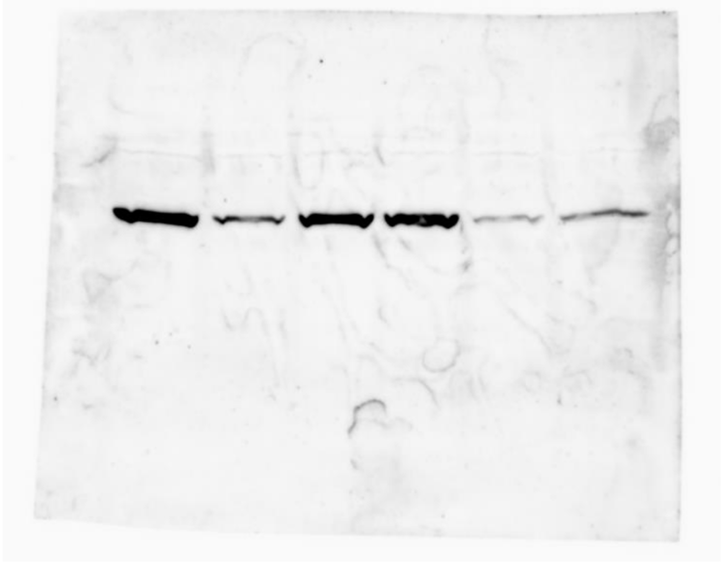

5

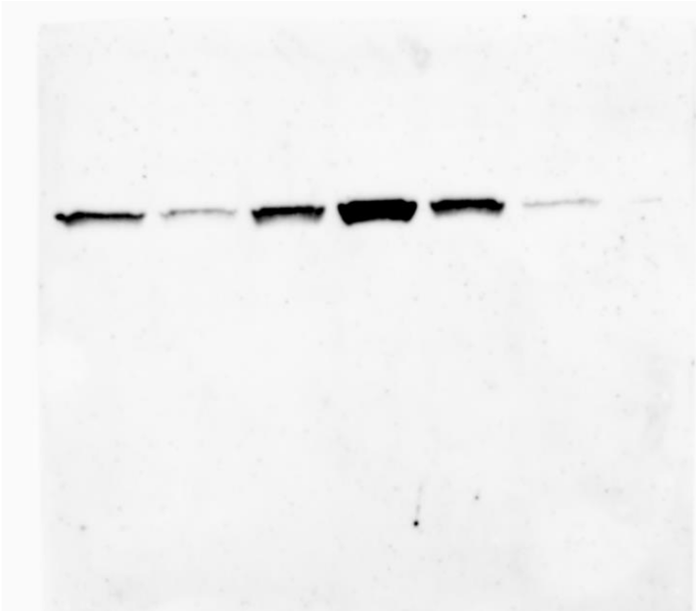

6

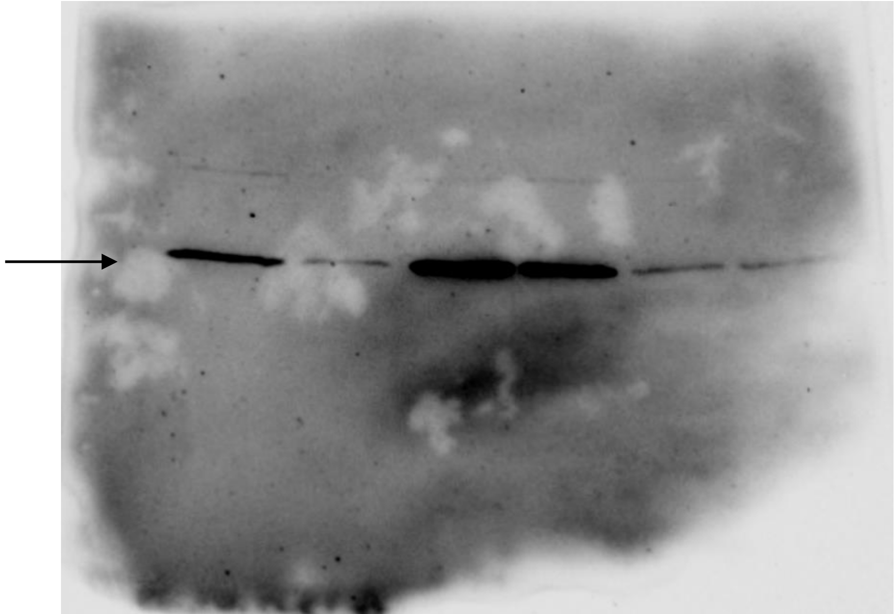

PPARγ - Ponceau S

This study

Samples order from the molecular weight marker: **MTX | DOX18 | CTRL** | DOX9-1 | CTRL-2 | DOX9-2

Other study

1

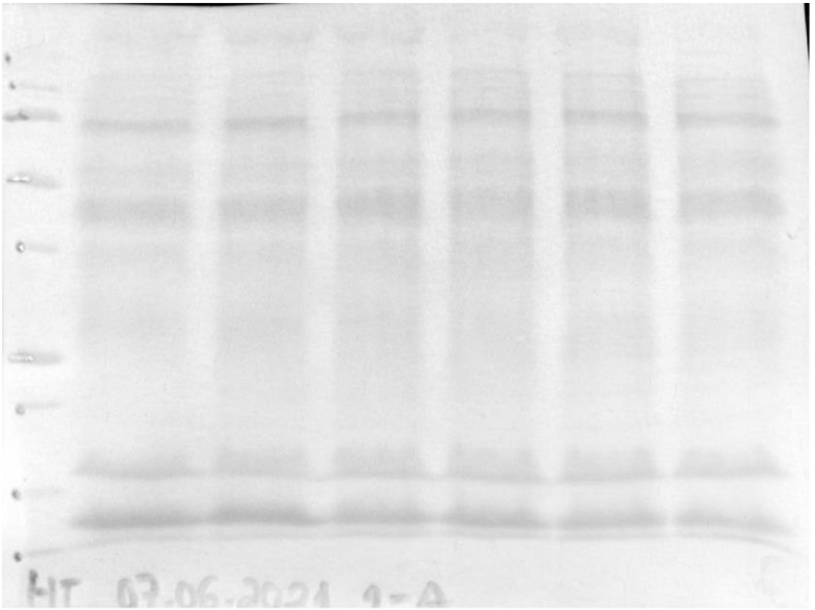

2

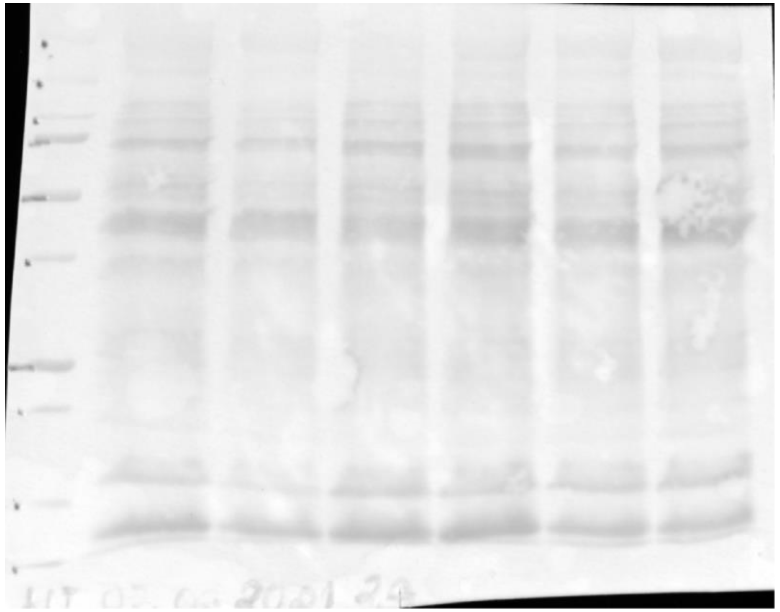

3

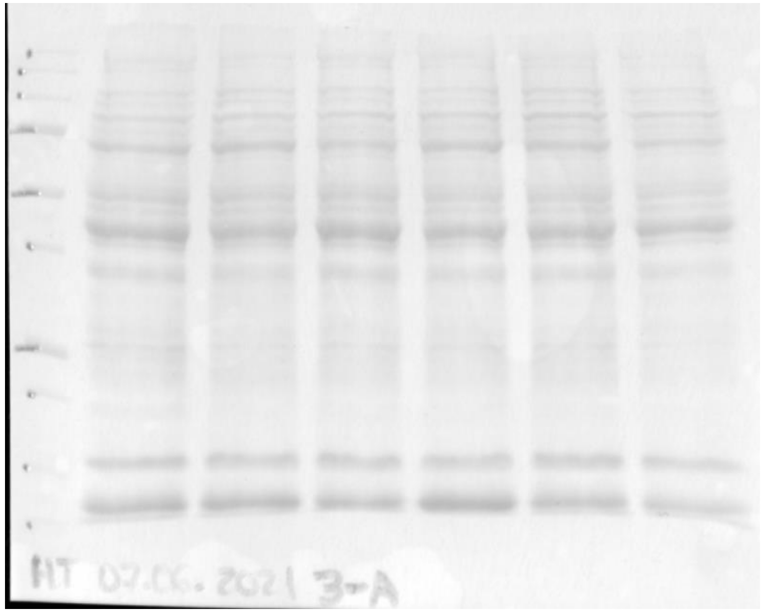

4

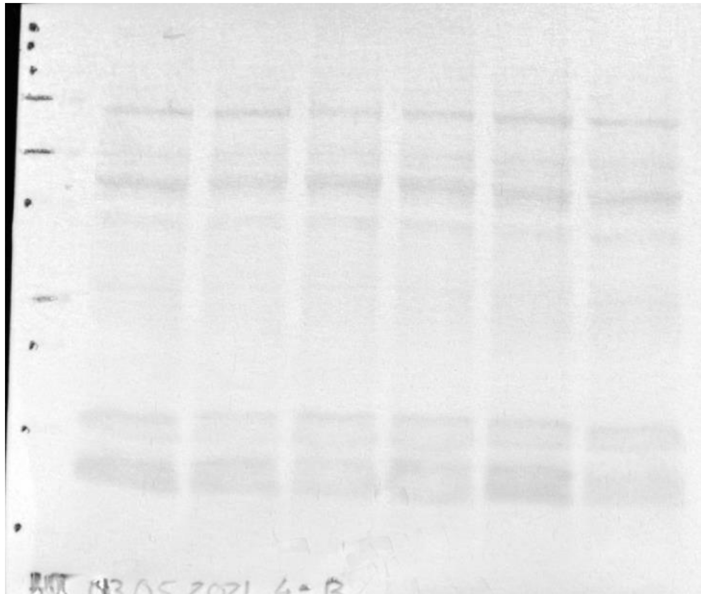

5

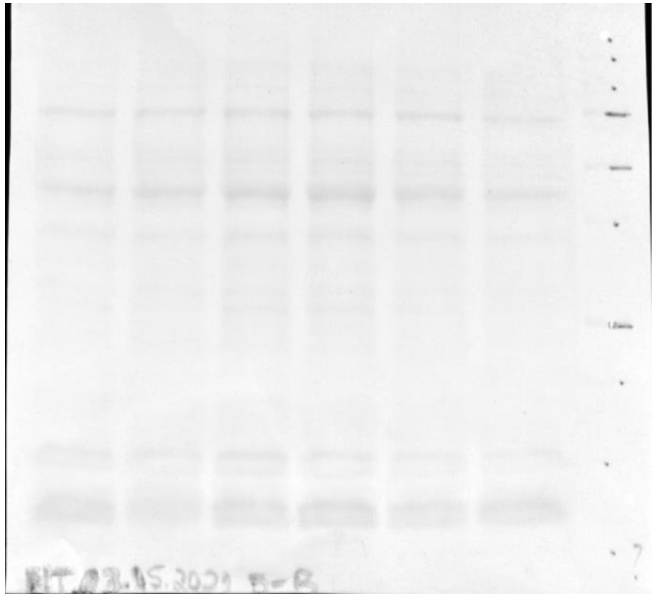

6

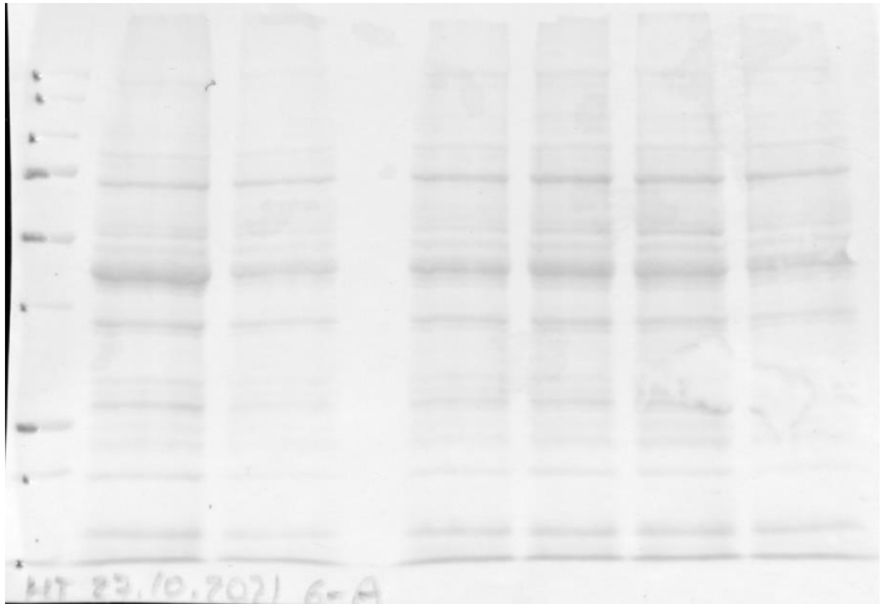

PPARγ - Blot

This study

Samples order from the molecular weight marker: **MTX | DOX18 | CTRL** | DOX9-1 | CTRL-2 | DOX9-2

Other study

1

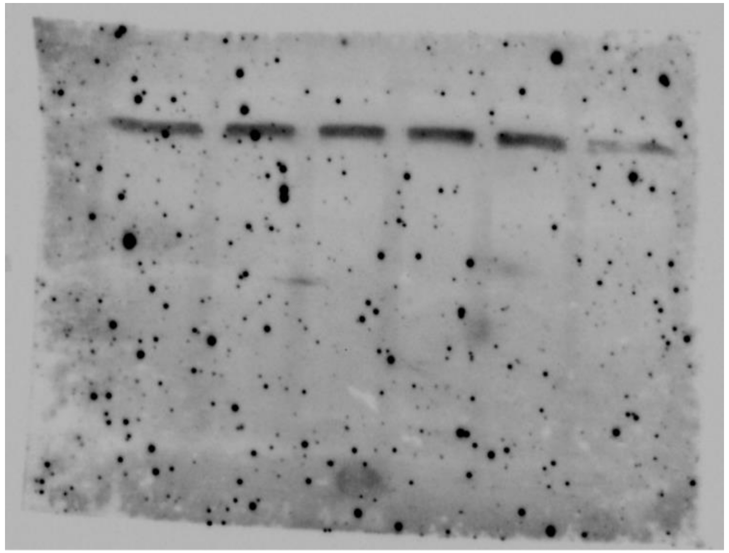

2

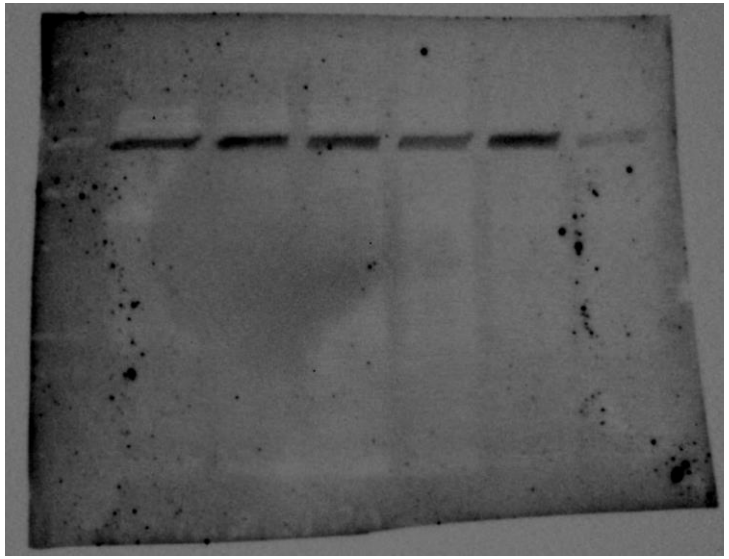

3

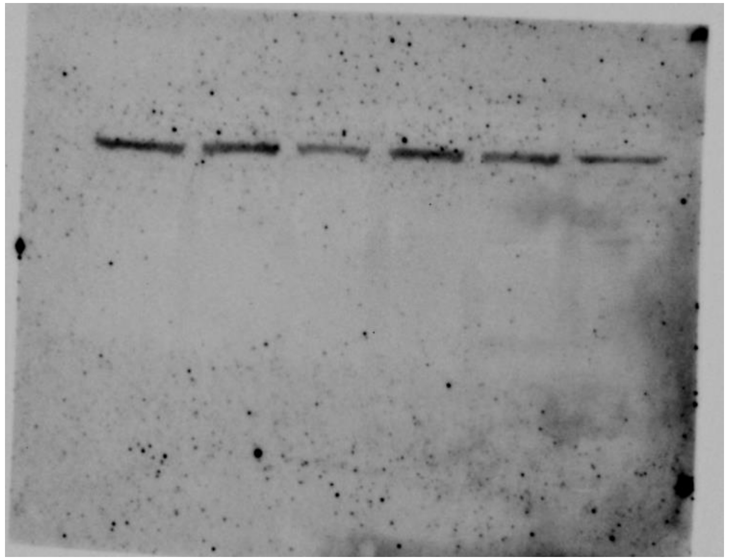

4

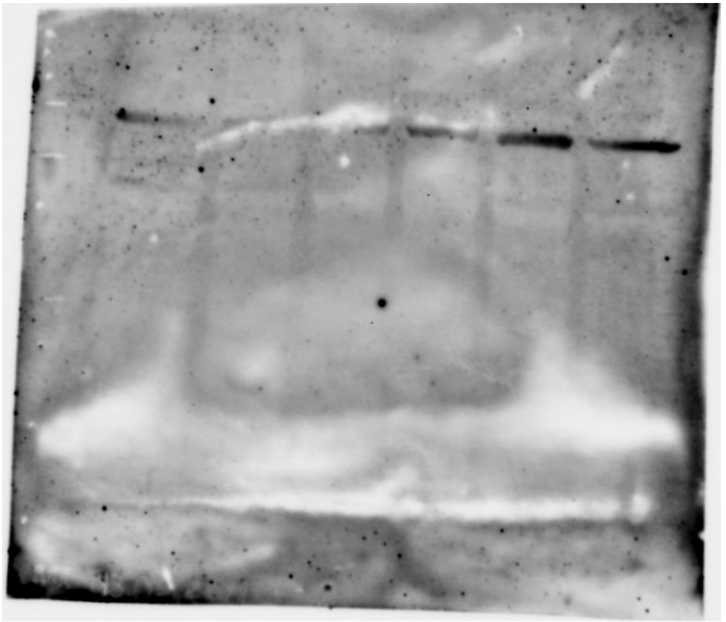

5

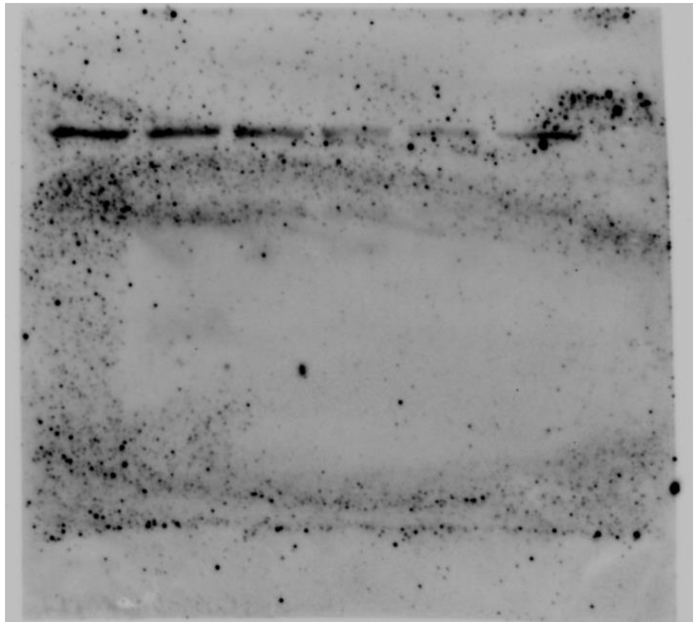

6

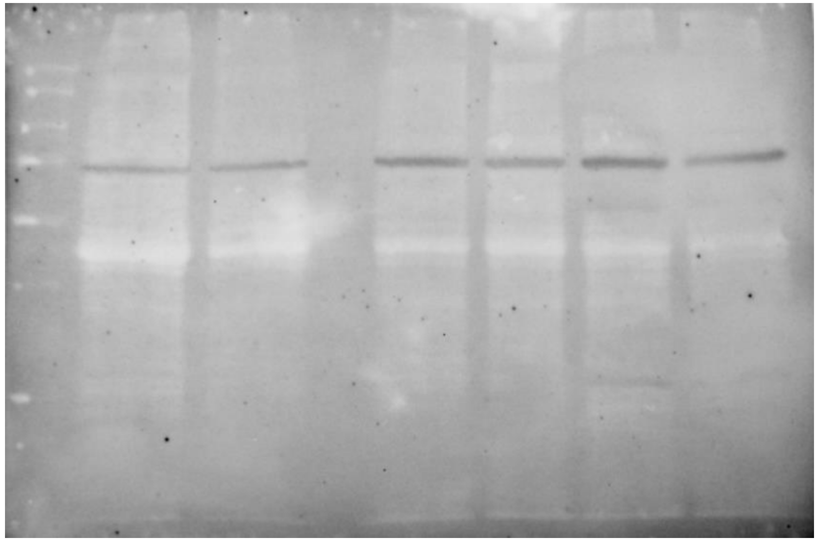

GSK-3β - Ponceau S

This study

Samples order from the molecular weight marker: **MTX | DOX18 | CTRL** | DOX9-1 | CTRL-2 | DOX9-2

Other study

1

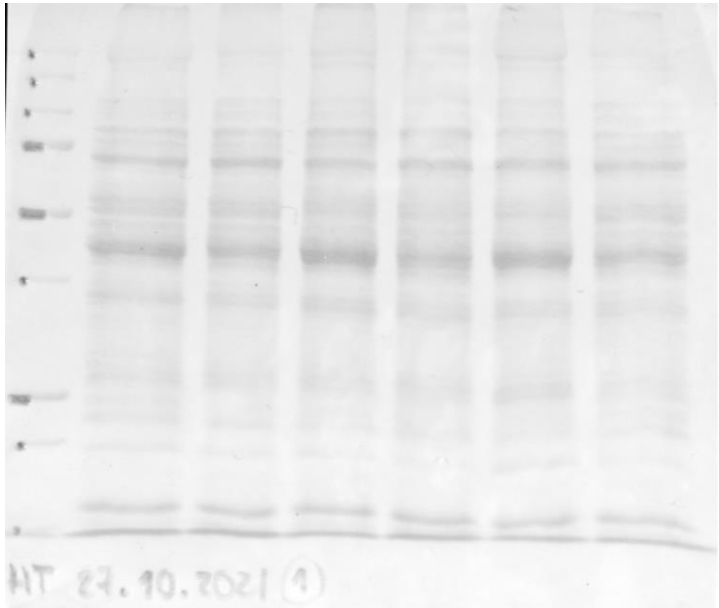

2

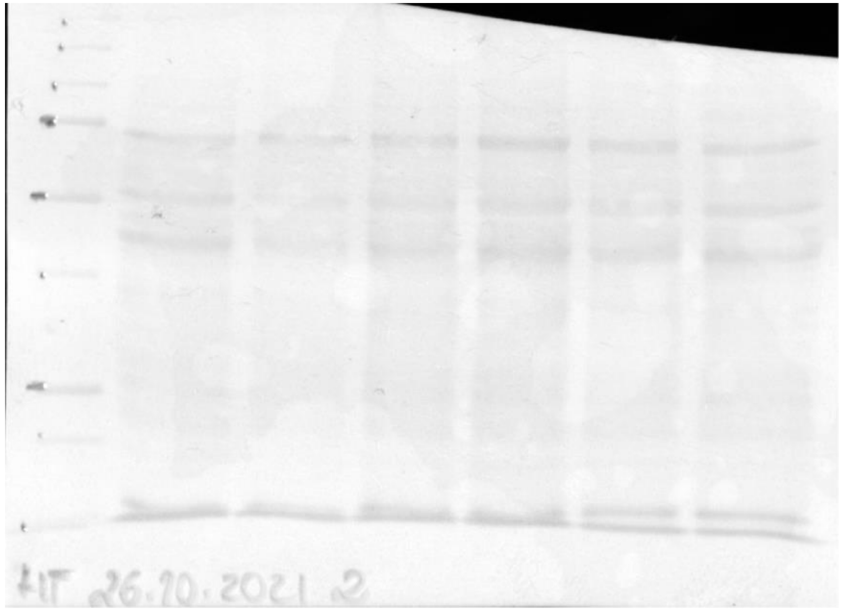

3

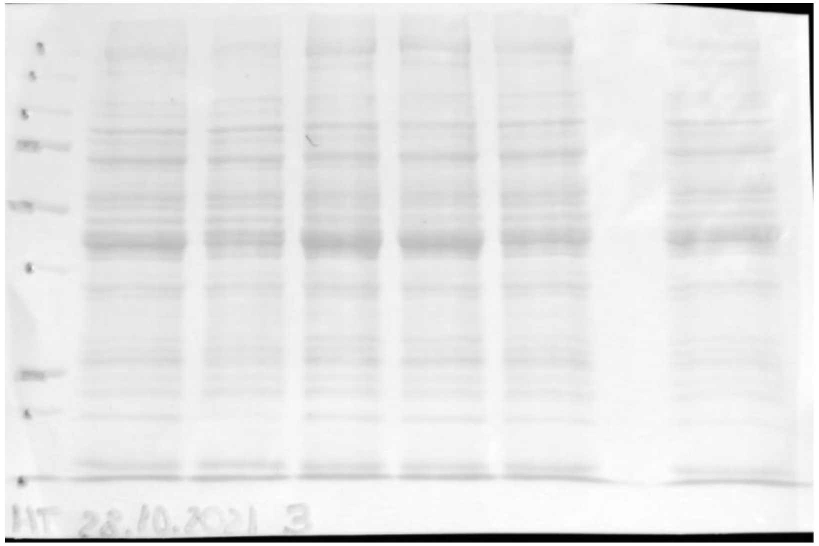

4

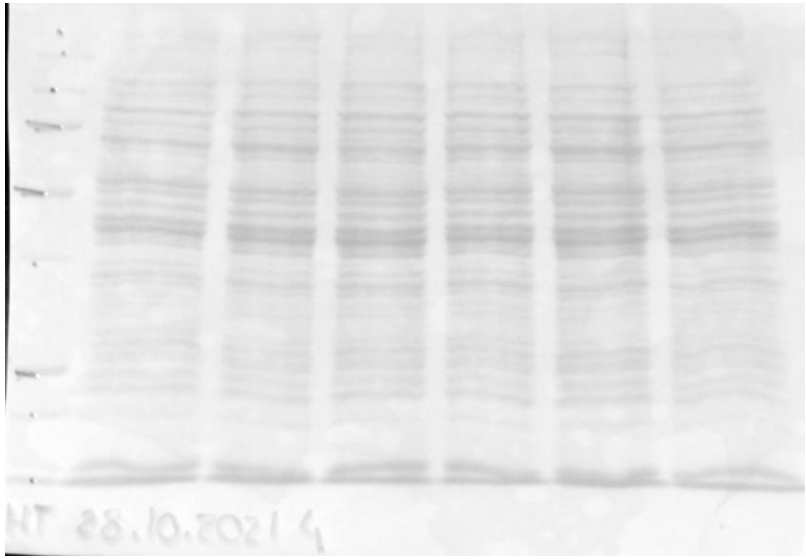

5

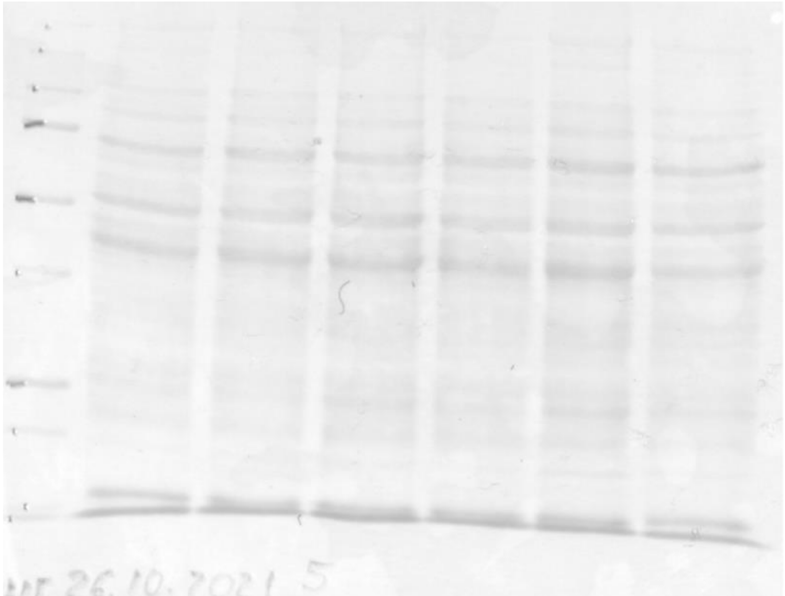

6

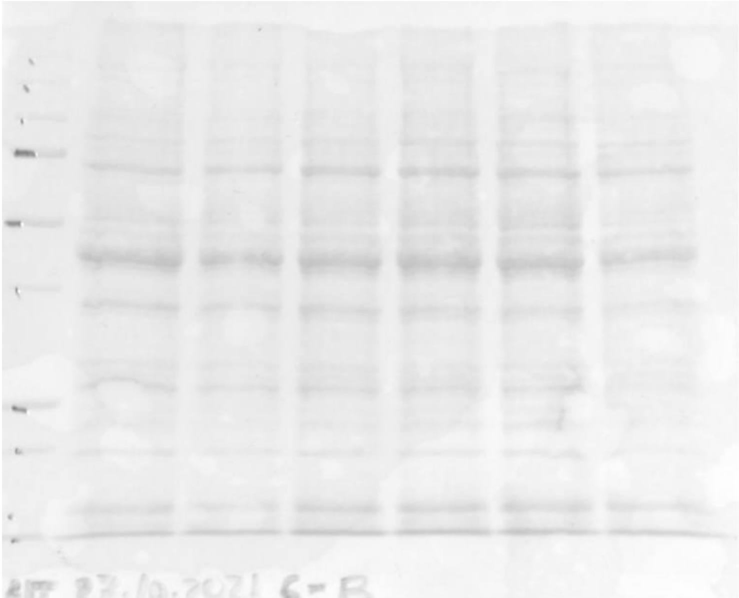

GSK-3β - Blot

This study

Samples order from the molecular weight marker: **MTX | DOX18 | CTRL** | DOX9-1 | CTRL-2 | DOX9-2

Other study

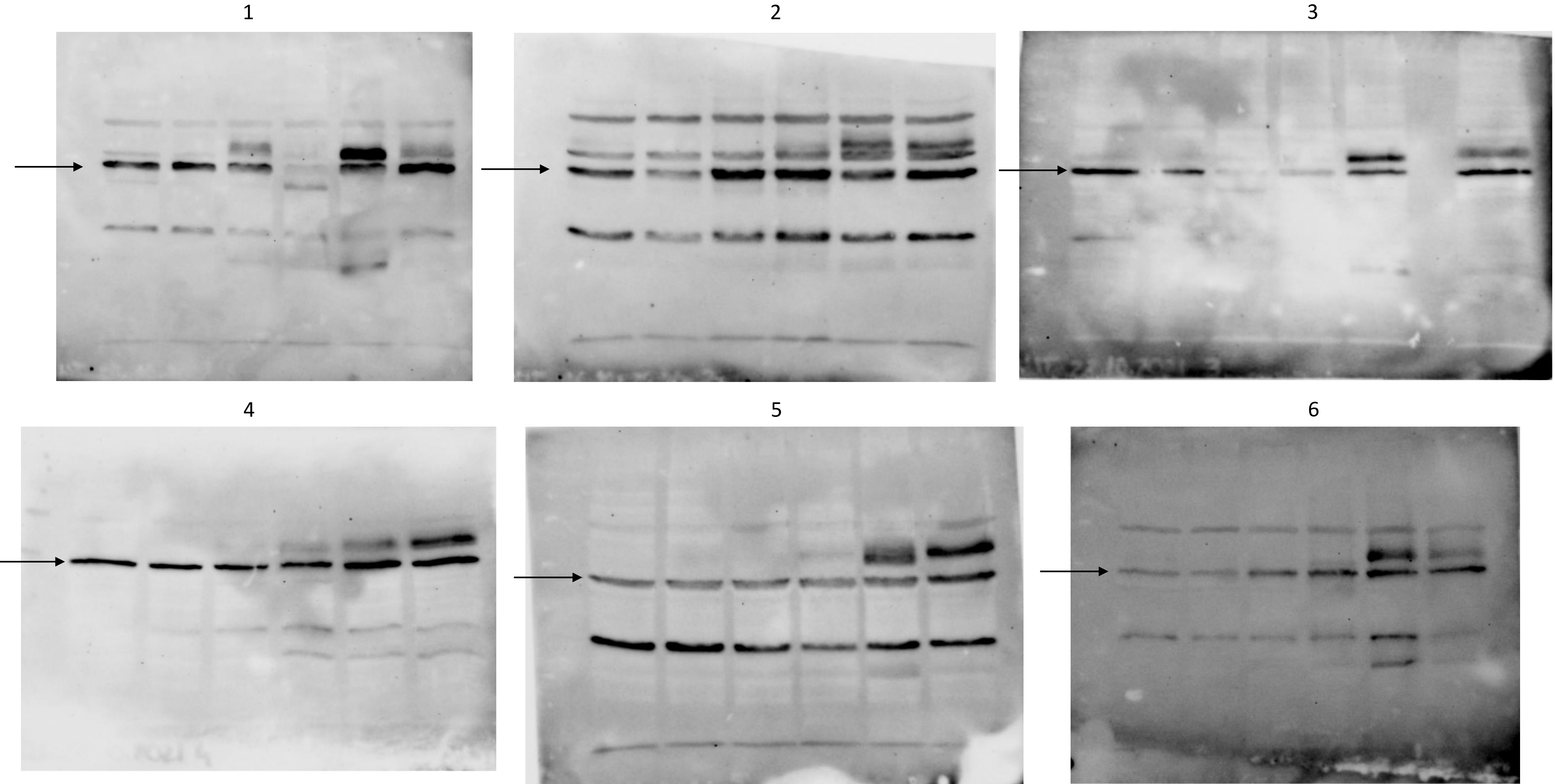

SIRT3 - Ponceau S

This study

Samples order from the molecular weight marker: **MTX | DOX18 | CTRL** | DOX9-1 | CTRL-2 | DOX9-2

Other study

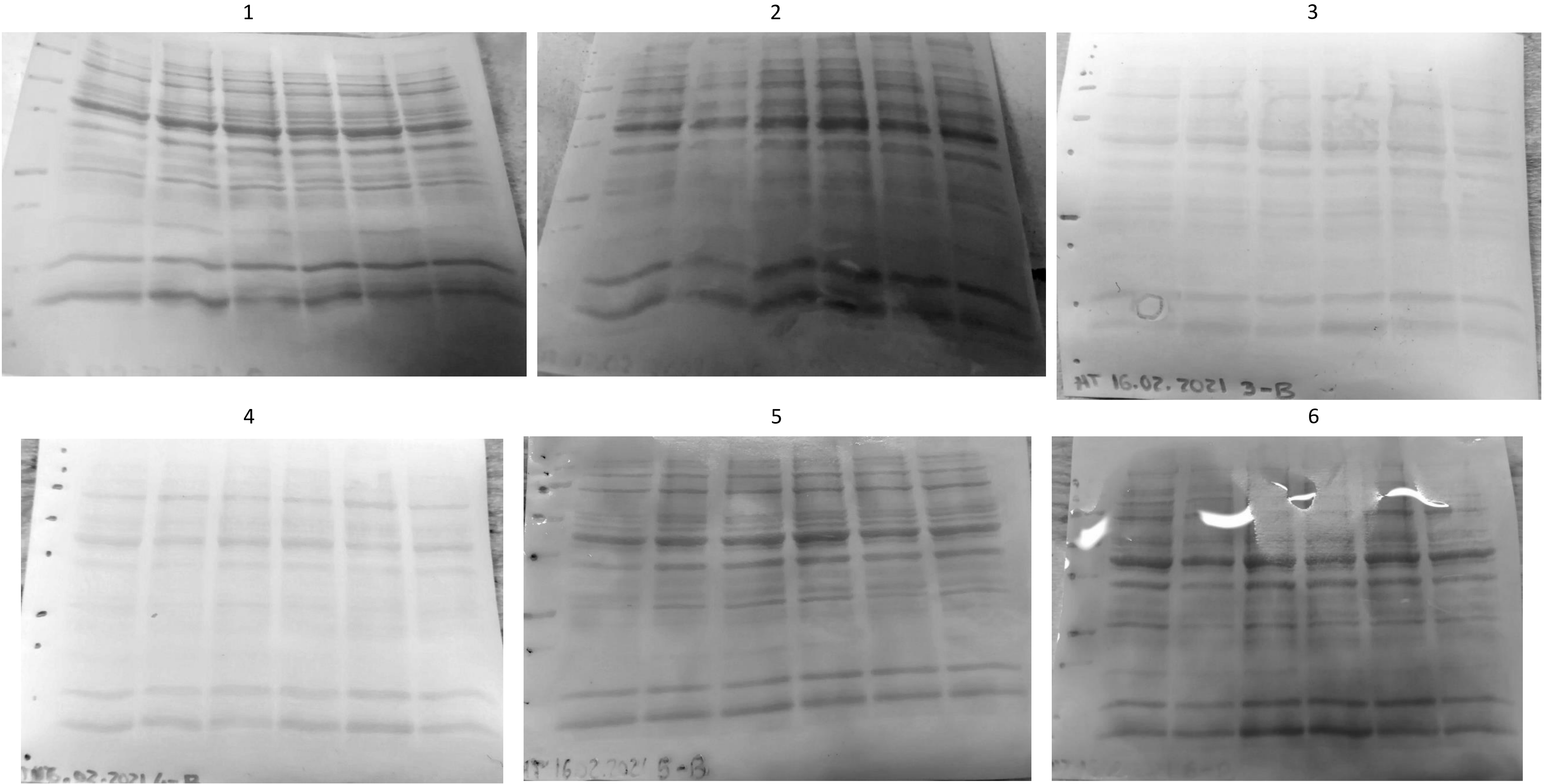

SIRT3 - Blot

This study

Samples order from the molecular weight marker: **MTX | DOX18 | CTRL** | DOX9-1 | CTRL-2 | DOX9-2

Other study

1

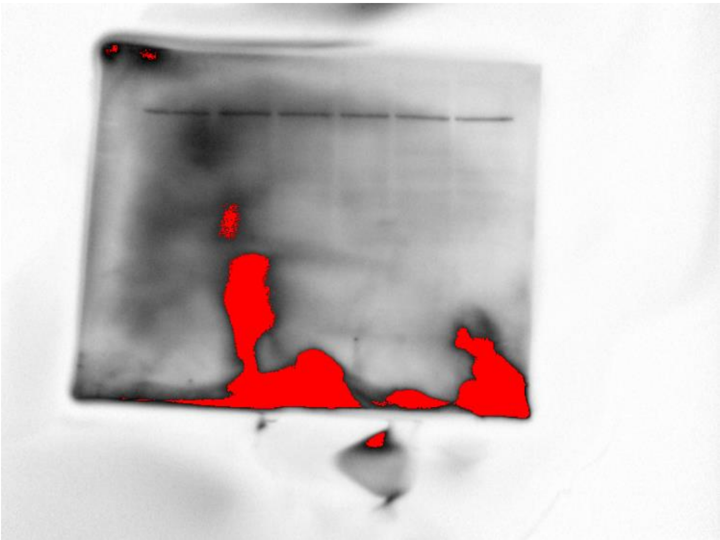

2

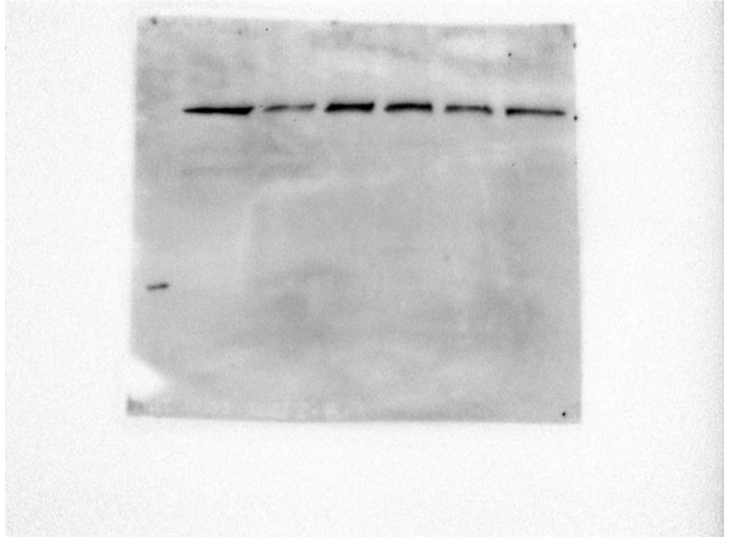

3

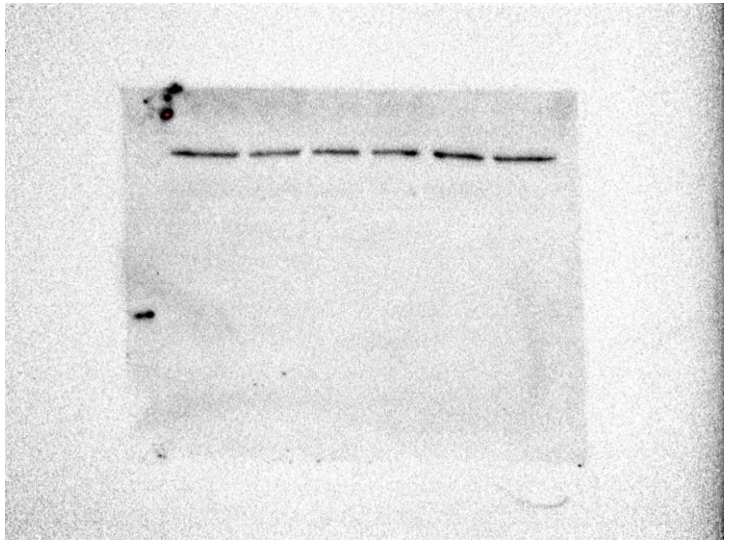

4

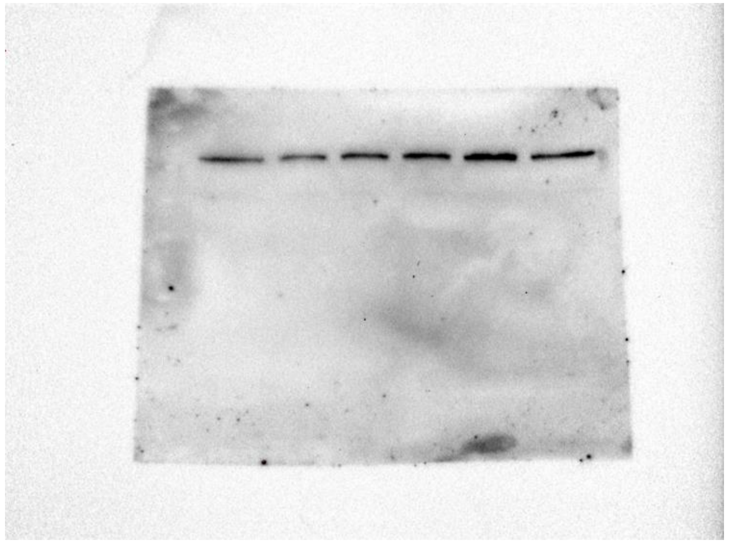

5

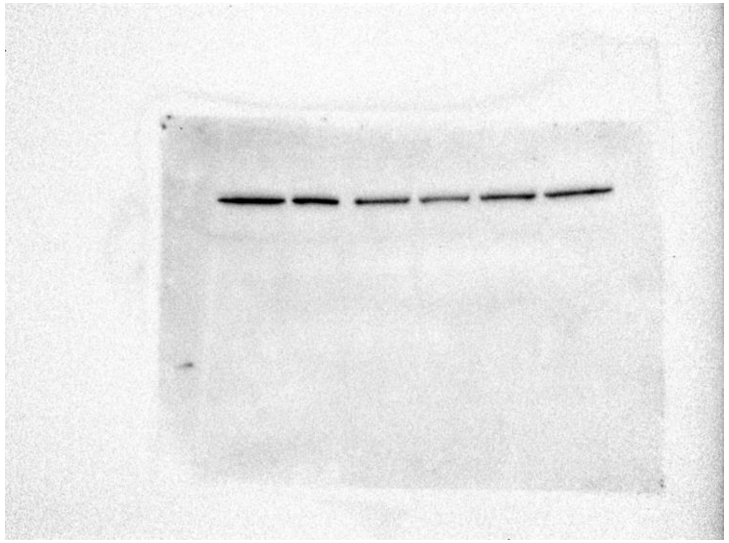

6

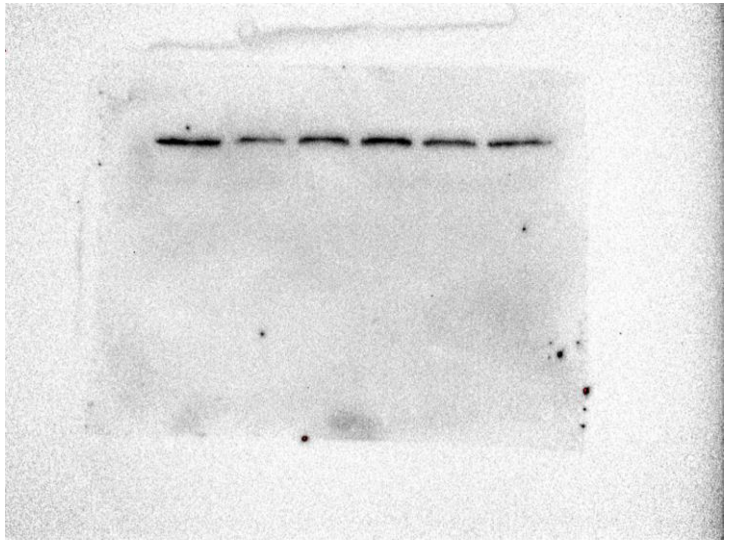

Tfam - Ponceau S

This study

Samples order from the molecular weight marker: **MTX | DOX18 | CTRL** | DOX9-1 | CTRL-2 | DOX9-2

Other study

1

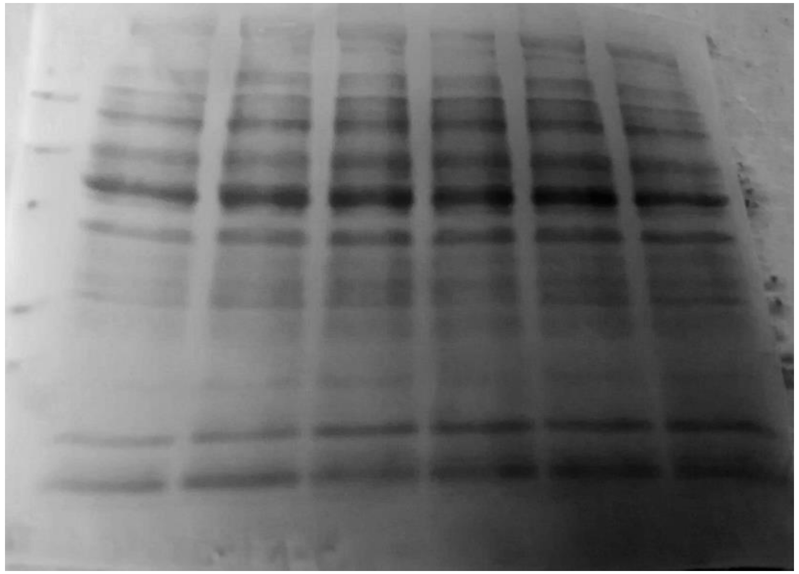

2

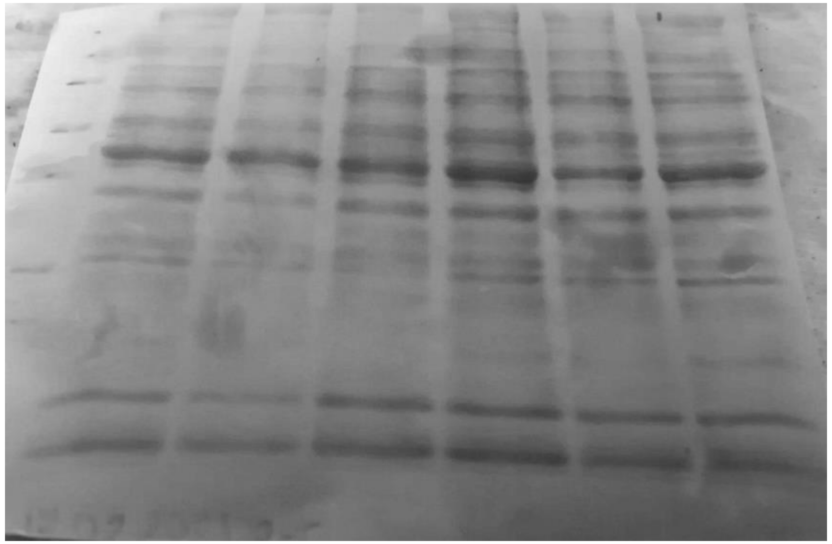

3

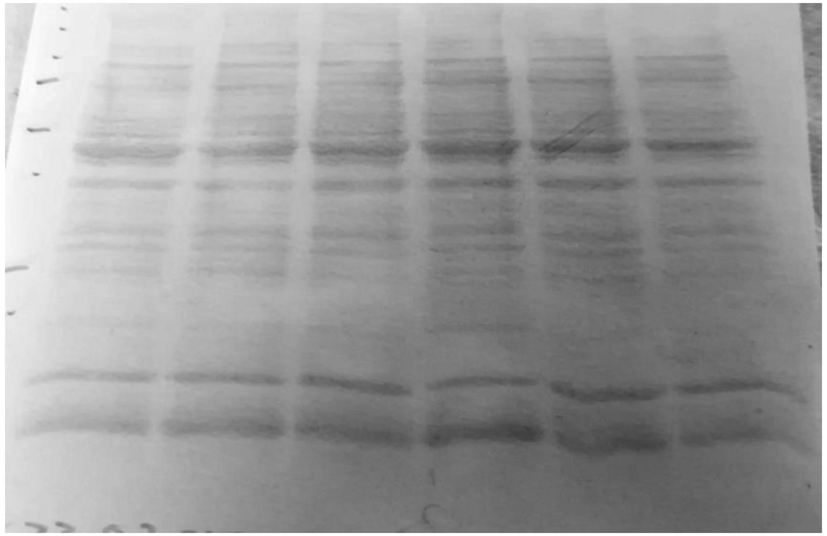

4

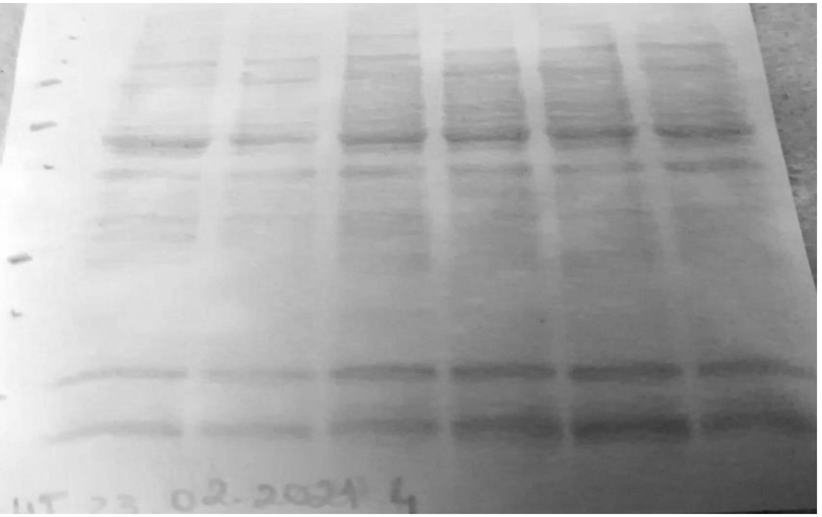

5

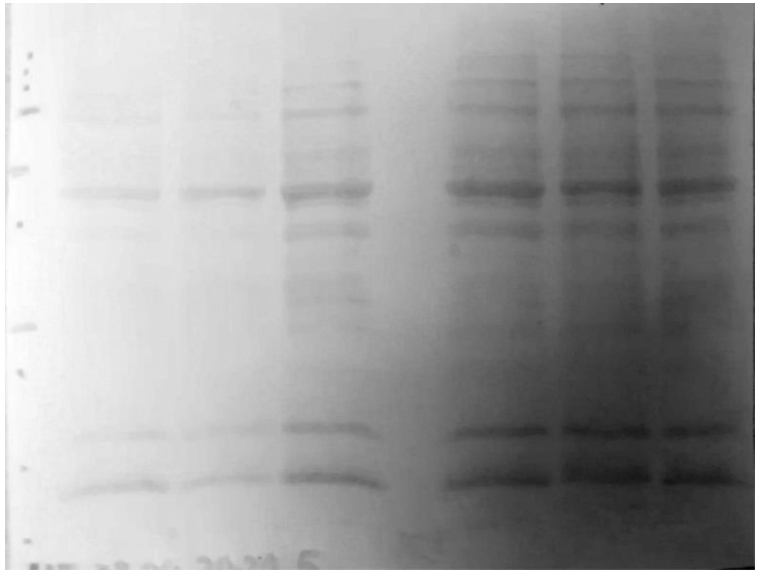

6

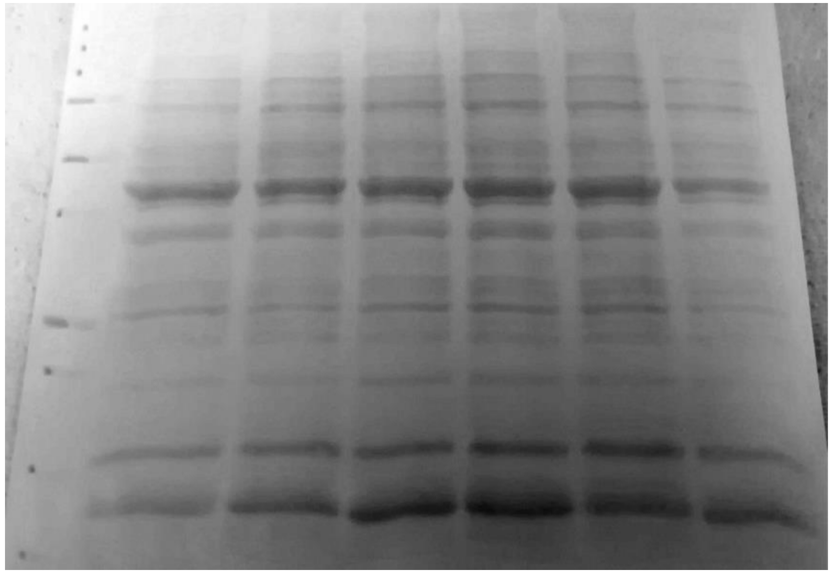

Tfam - Blot

This study

Samples order from the molecular weight marker: **MTX | DOX18 | CTRL** | DOX9-1 | CTRL-2 | DOX9-2

Other study

1

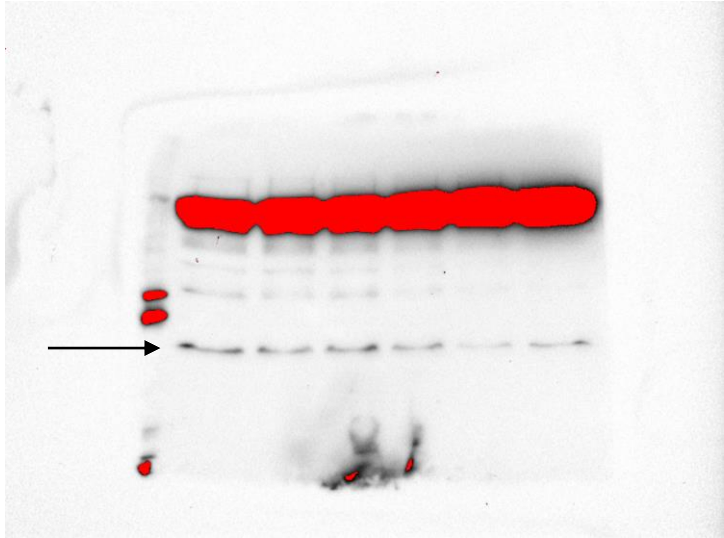

2

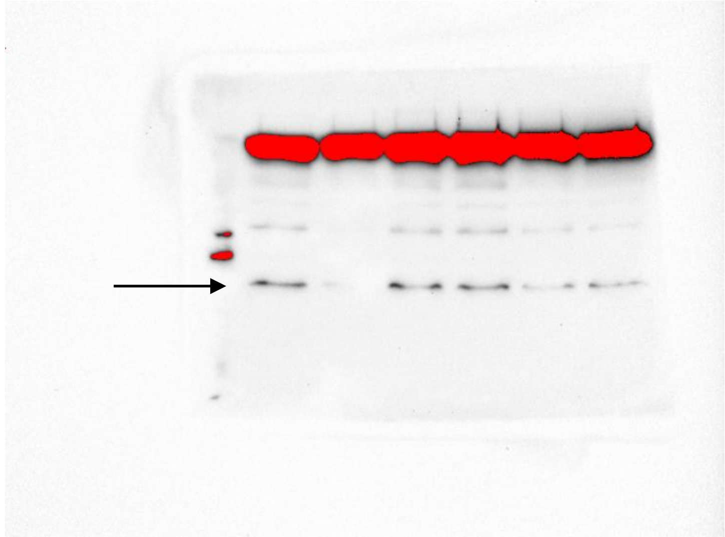

3

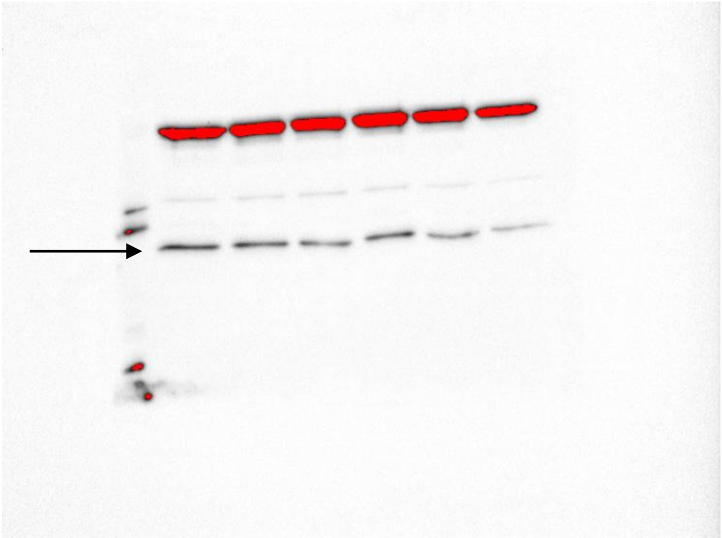

4

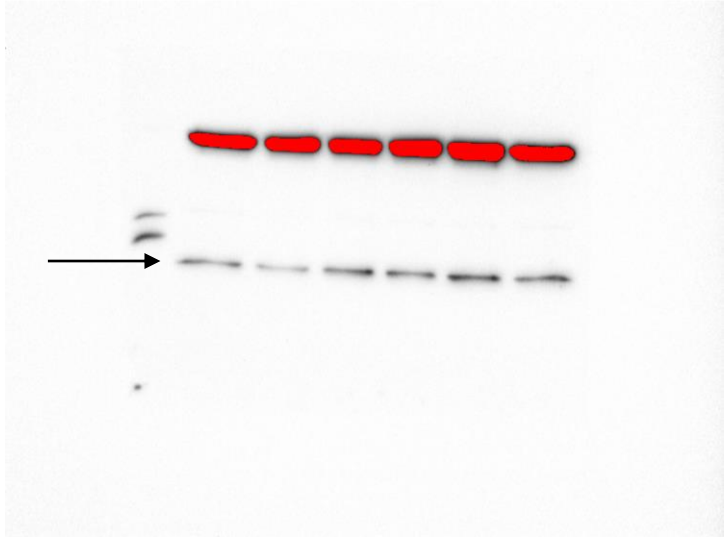

5

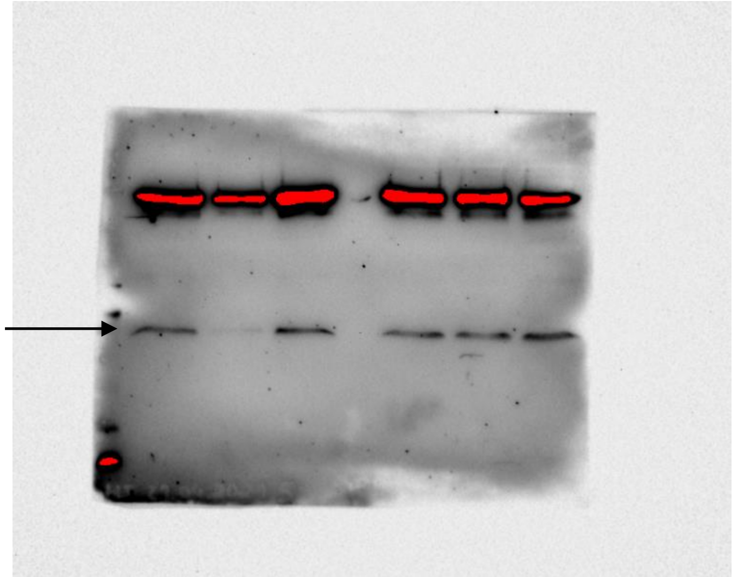

6

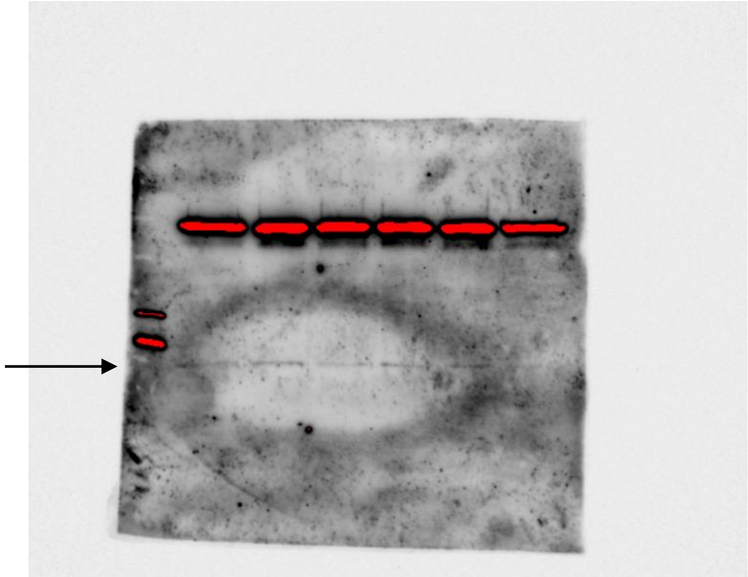

PGC-1α - Ponceau S

This study

Samples order from the molecular weight marker: **MTX | DOX18 | CTRL** | DOX9-1 | CTRL-2 | DOX9-2

Other study

1

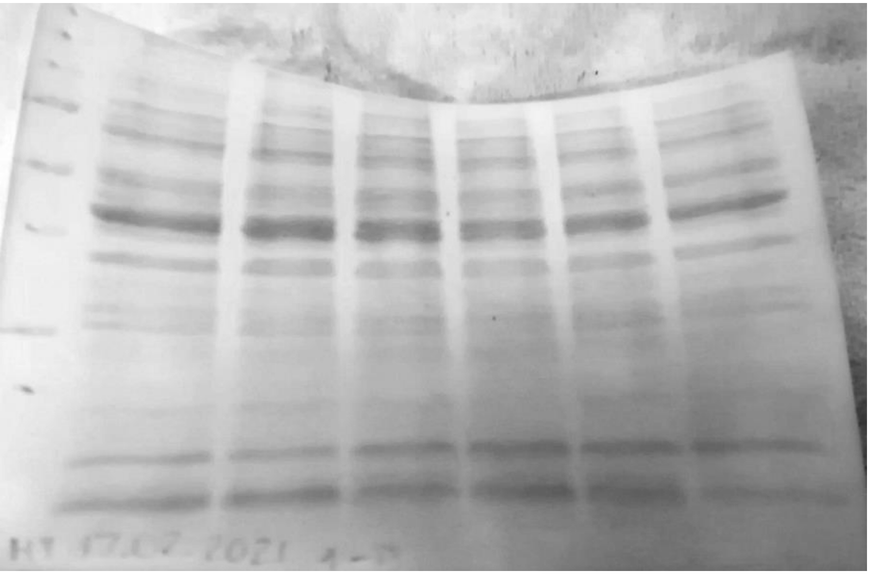

2

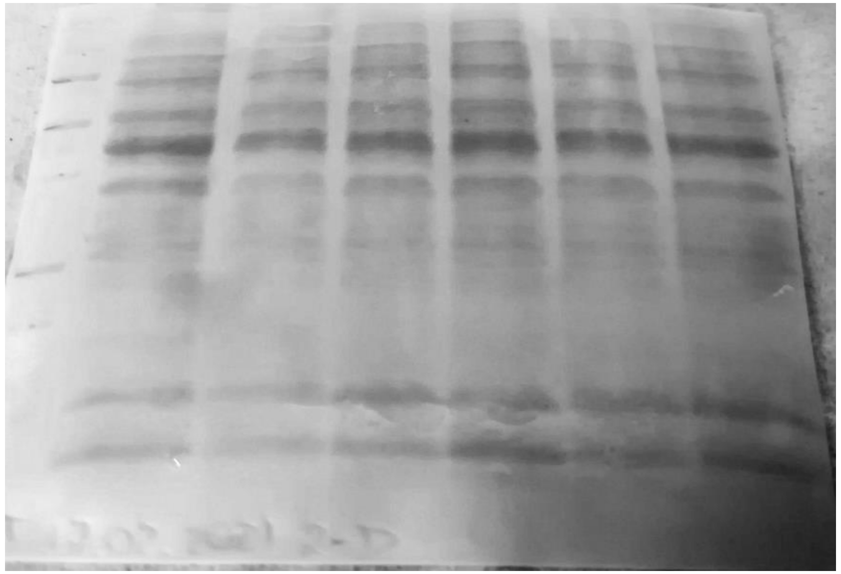

3

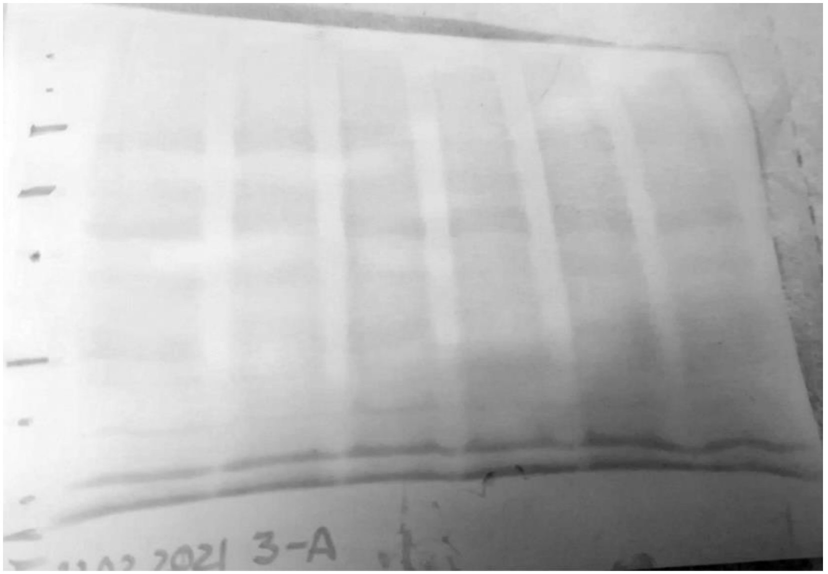

4

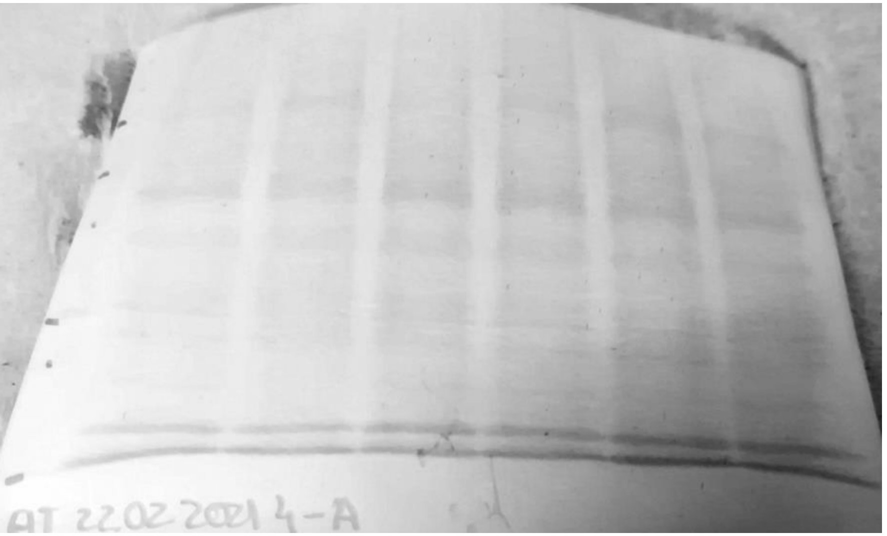

5

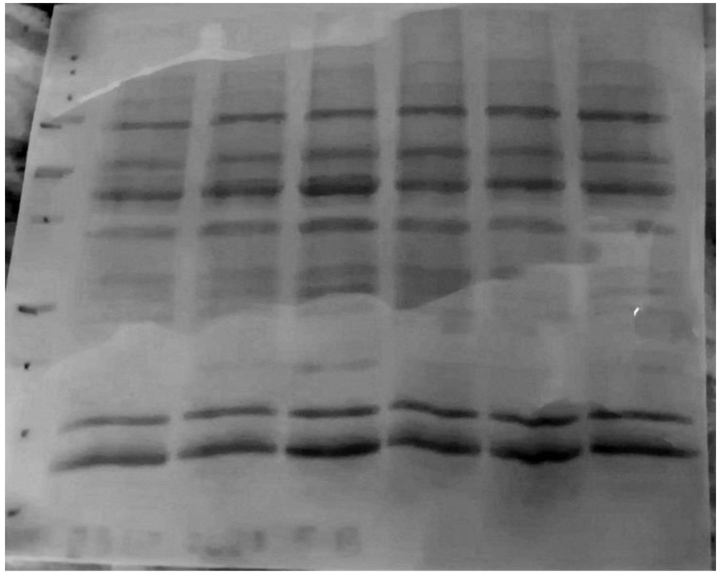

6

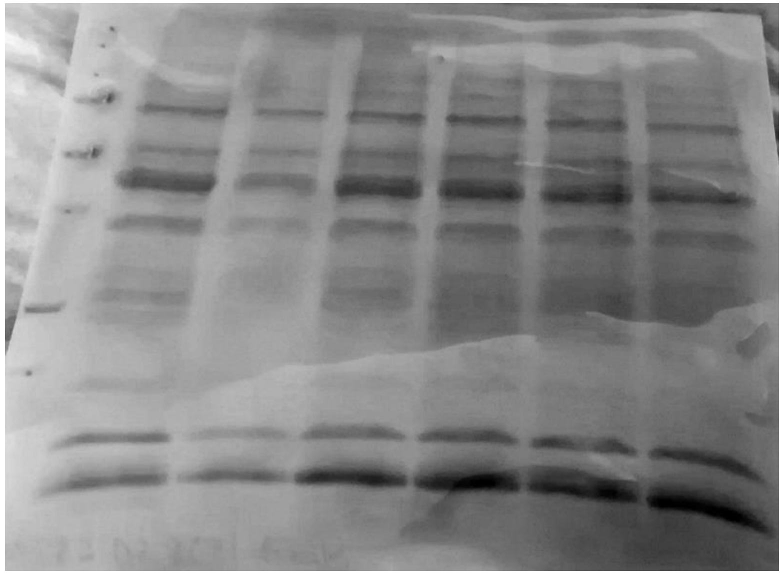

PGC-1α - Blot

This study

Samples order from the molecular weight marker: **MTX | DOX18 | CTRL** | DOX9-1 | CTRL-2 | DOX9-2

Other study

1

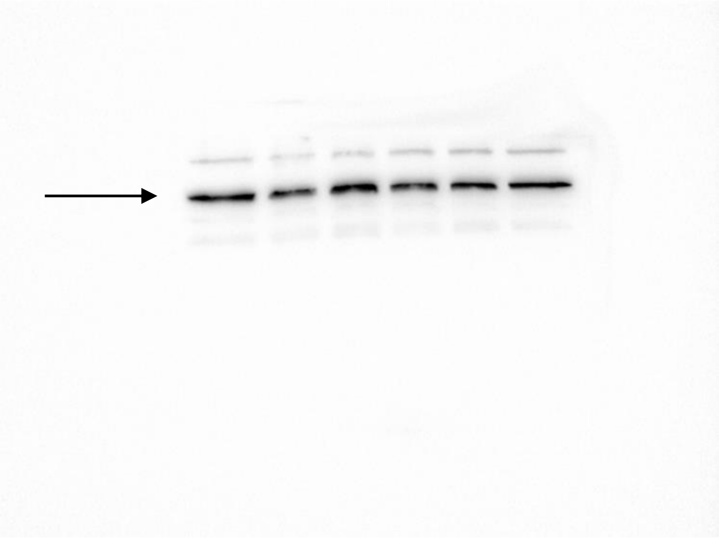

2

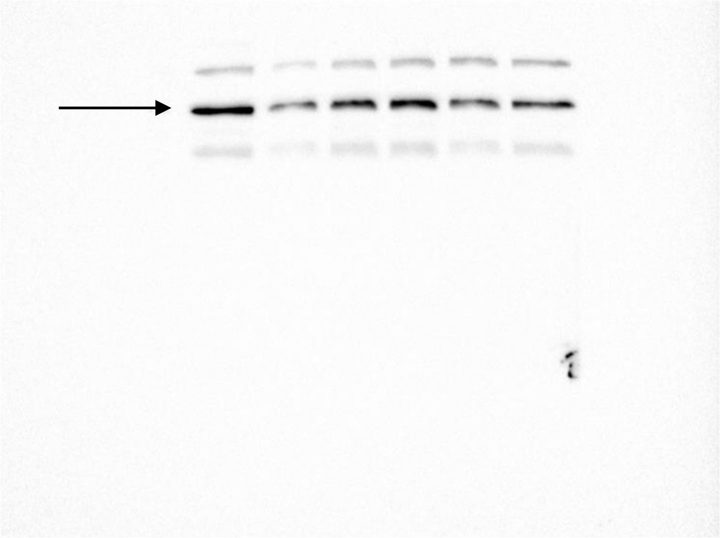

3

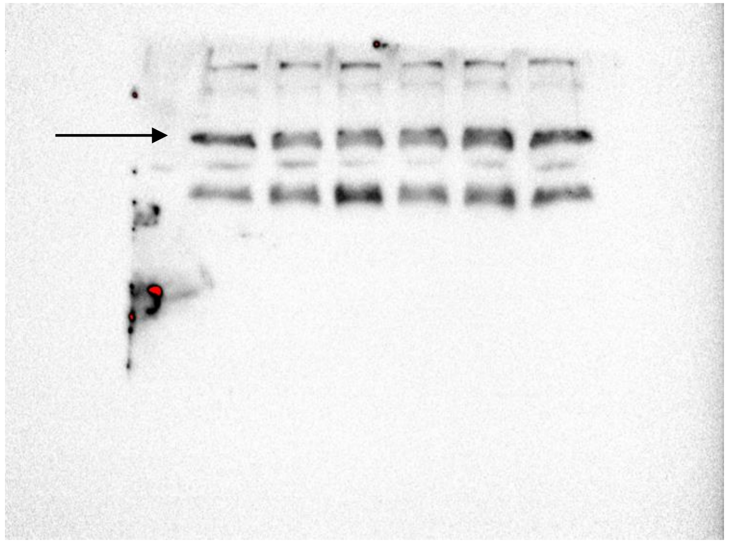

4

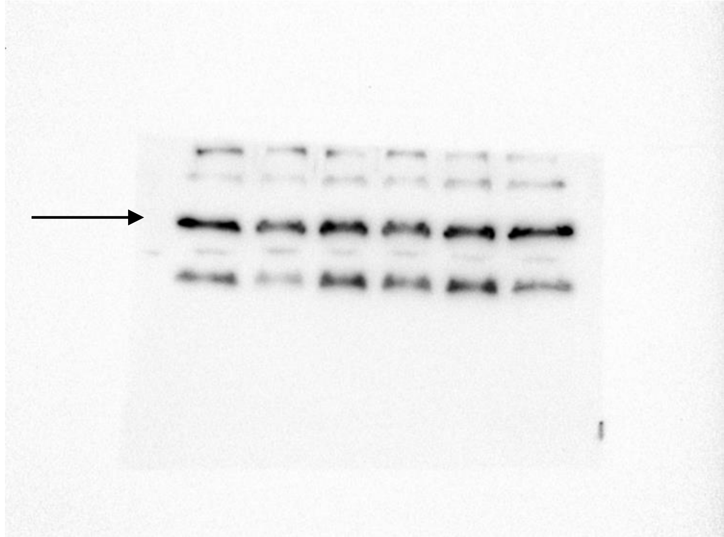

5

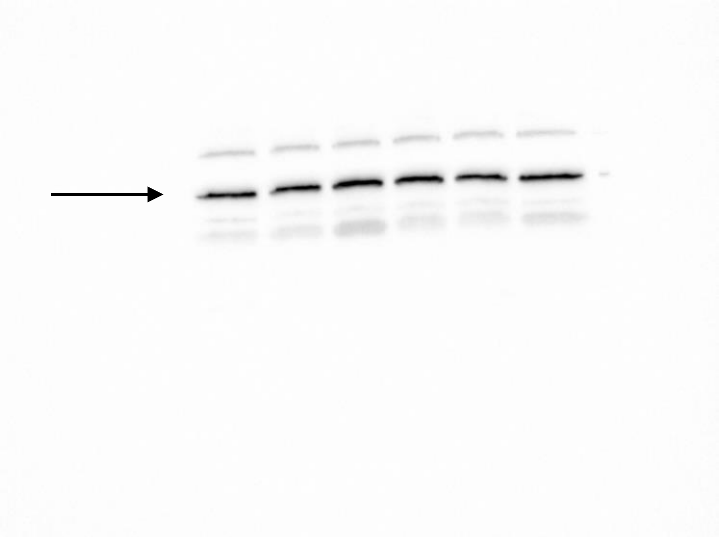

6

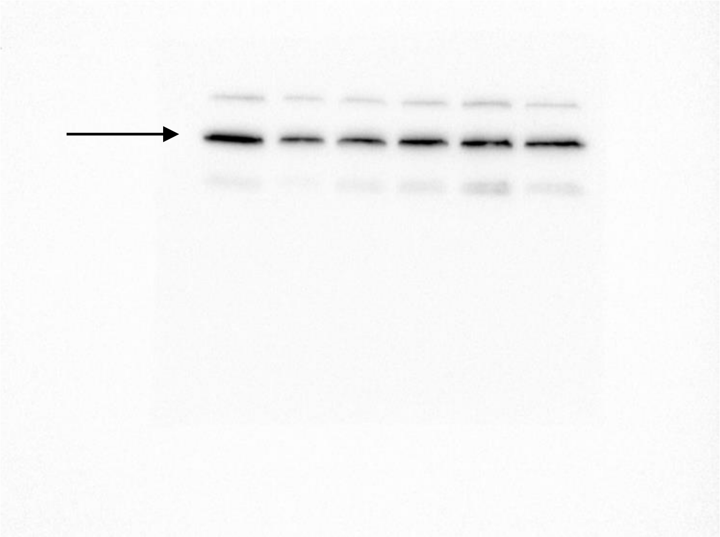

Mfn1 - Ponceau S

This study

Samples order from the molecular weight marker: **MTX | DOX18 | CTRL** | DOX9-1 | CTRL-2 | DOX9-2

Other study

1

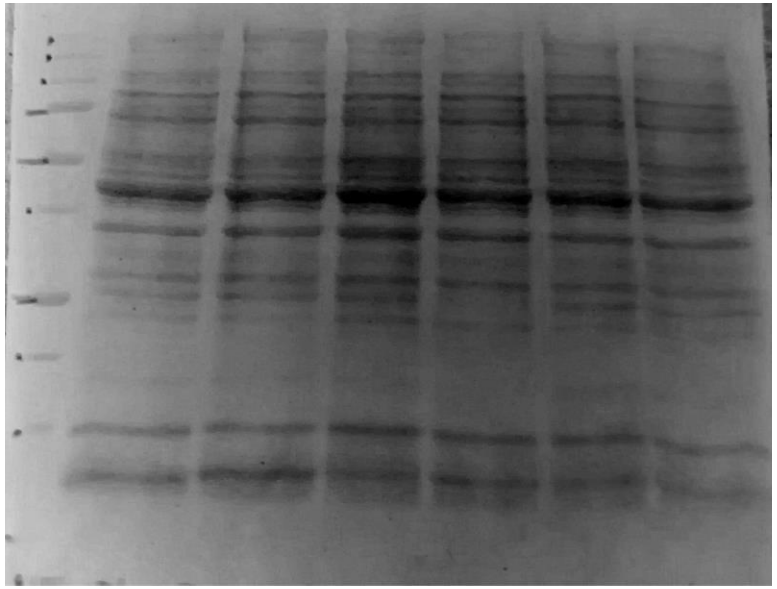

2

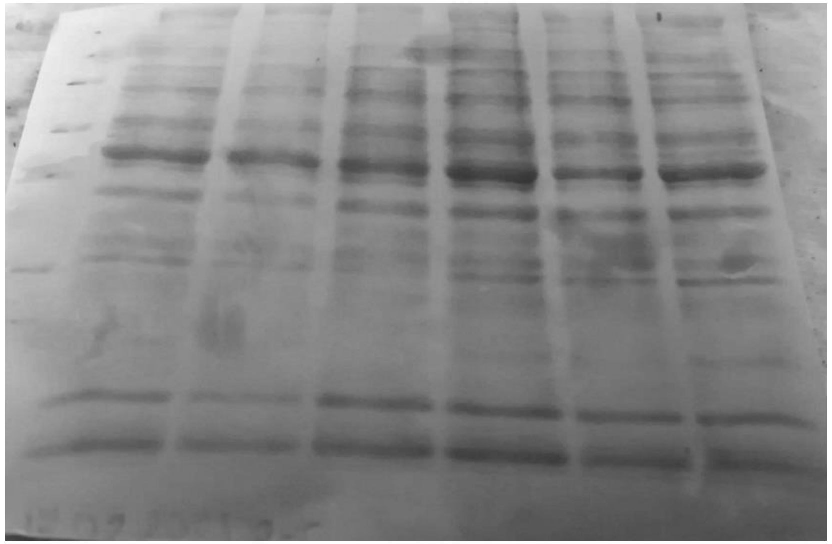

3

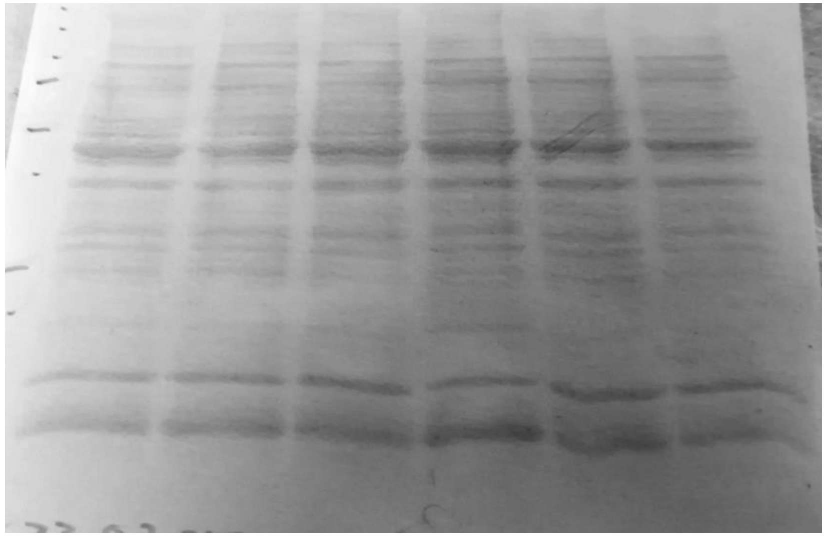

4

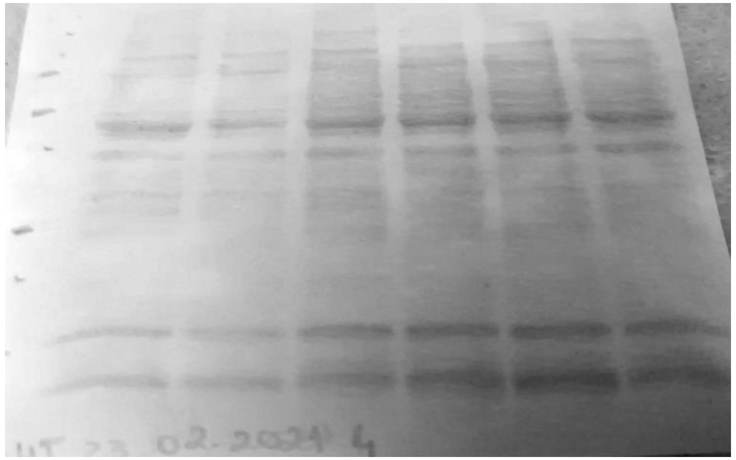

5

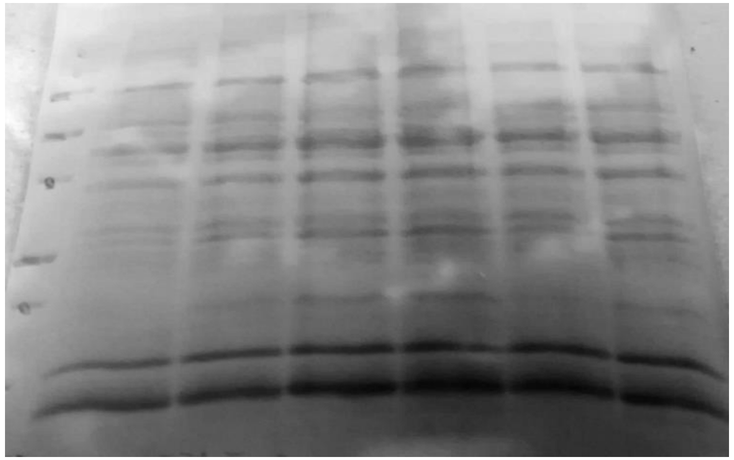

6

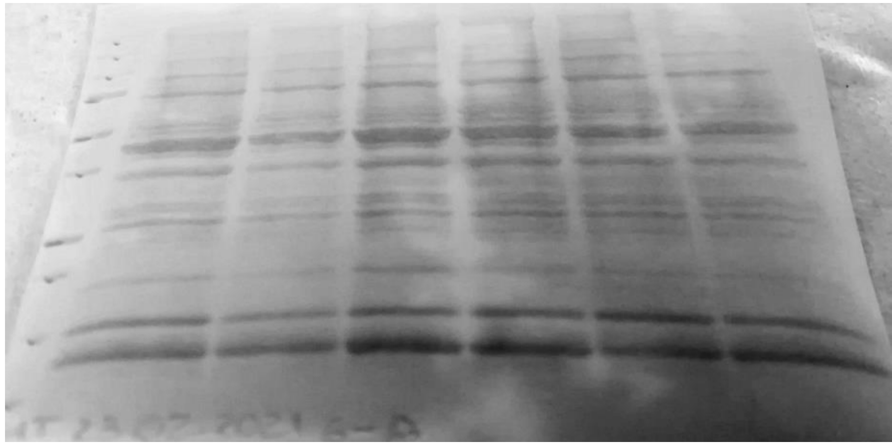

Mfn1 - Blot

This study

Samples order from the molecular weight marker: **MTX | DOX18 | CTRL** | DOX9-1 | CTRL-2 | DOX9-2

Other study

1

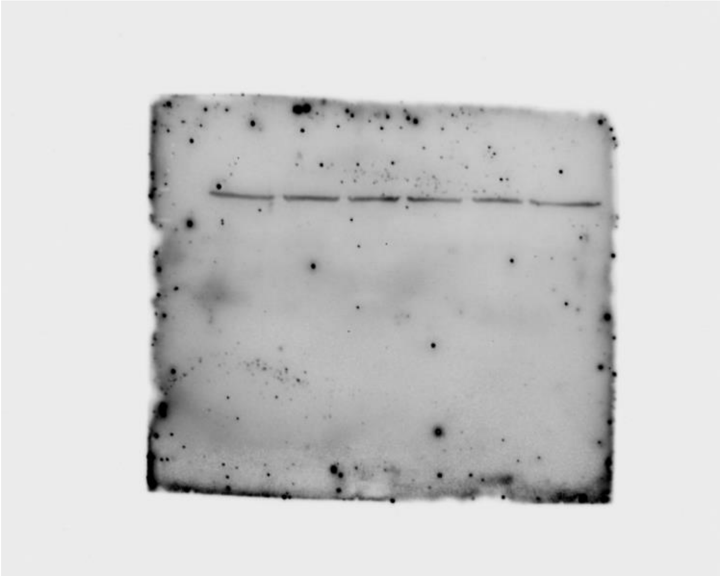

2

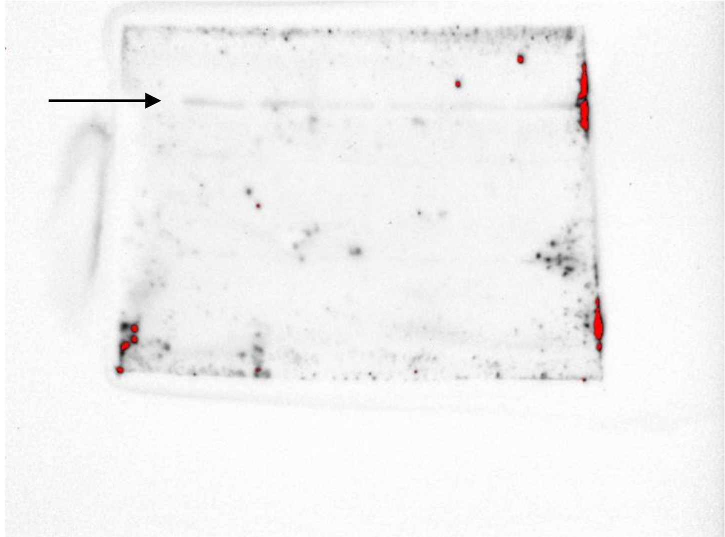

3

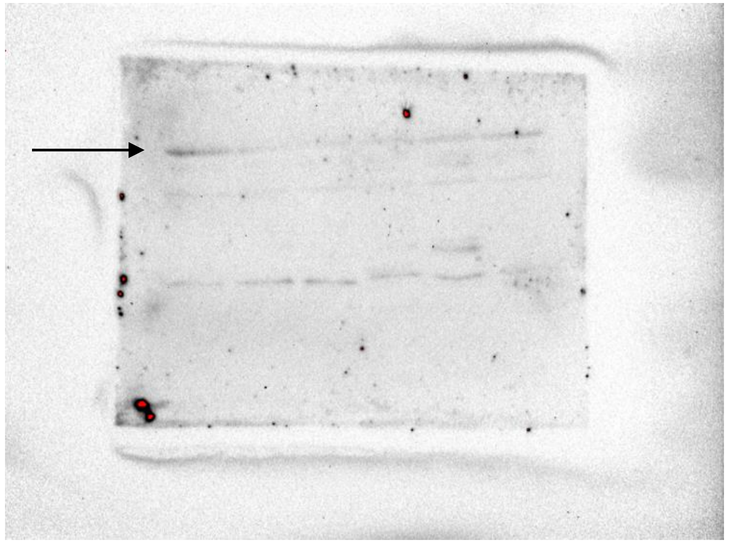

4

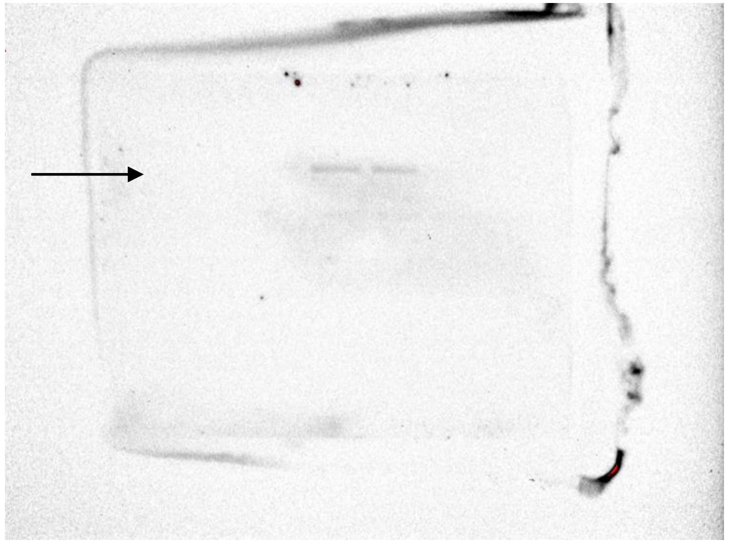

5

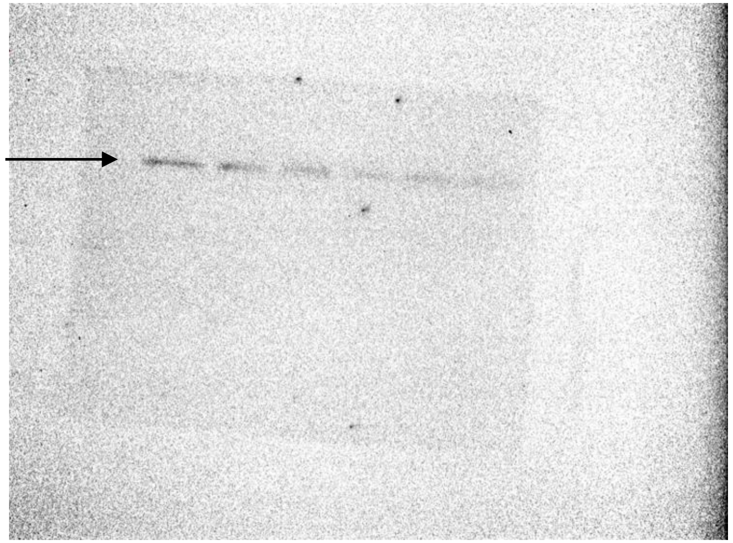

6

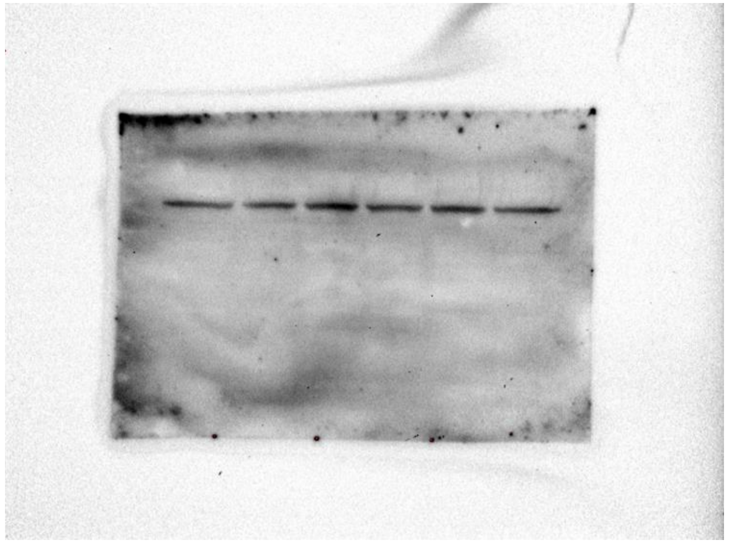

Beclin1 - Ponceau S

This study

Samples order from the molecular weight marker: **MTX | DOX18 | CTRL** | DOX9-1 | CTRL-2 | DOX9-2

Other study

1

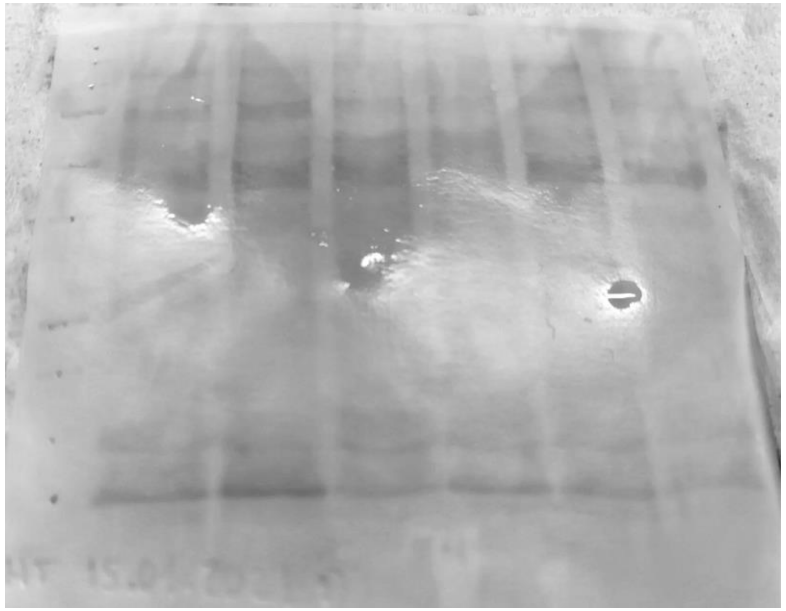

2

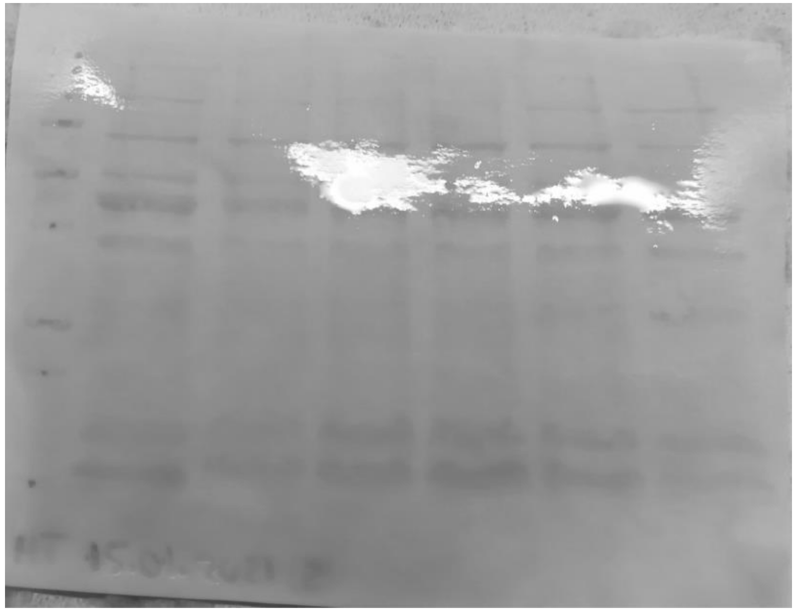

3

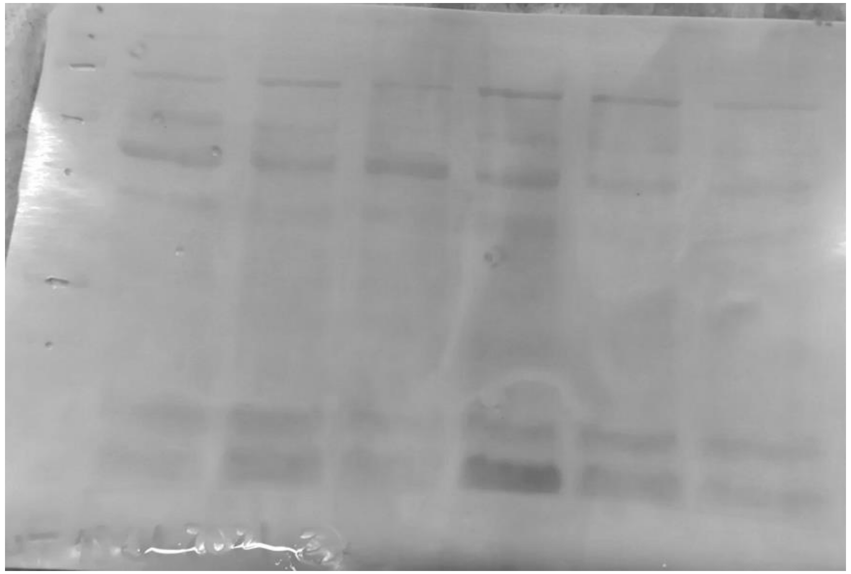

4

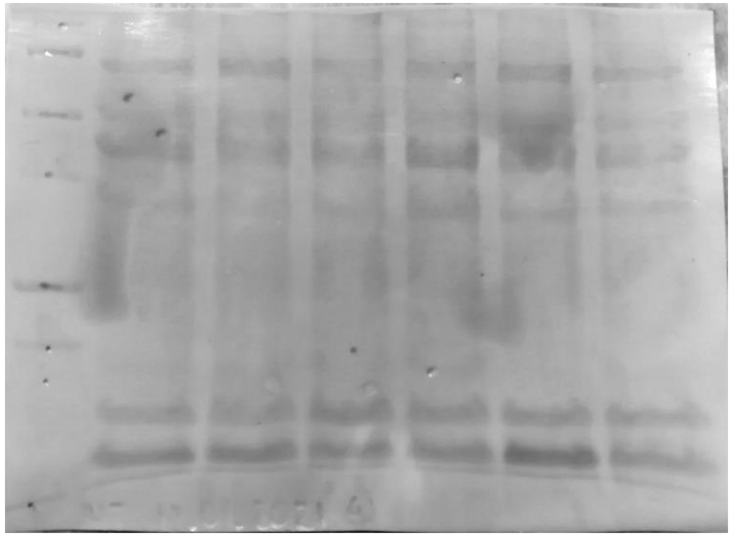

5

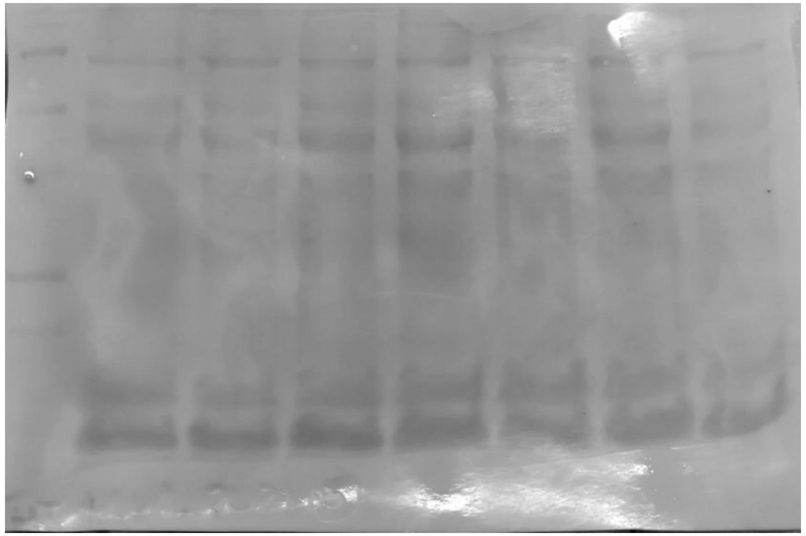

6

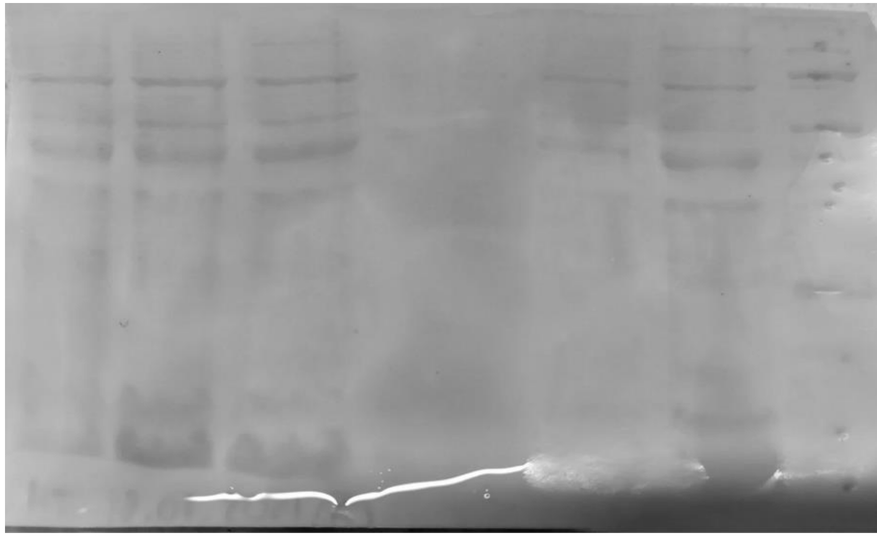

Beclin1 - Blot

This study

Samples order from the molecular weight marker: **MTX | DOX18 | CTRL** | DOX9-1 | CTRL-2 | DOX9-2

Other study

1

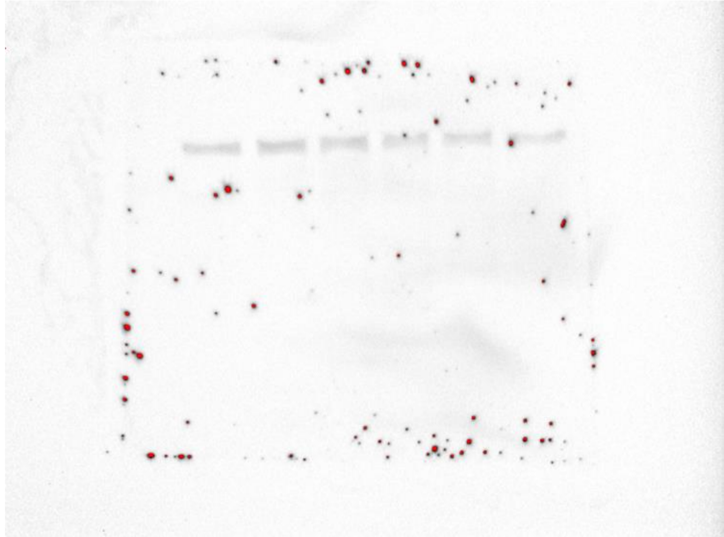

2

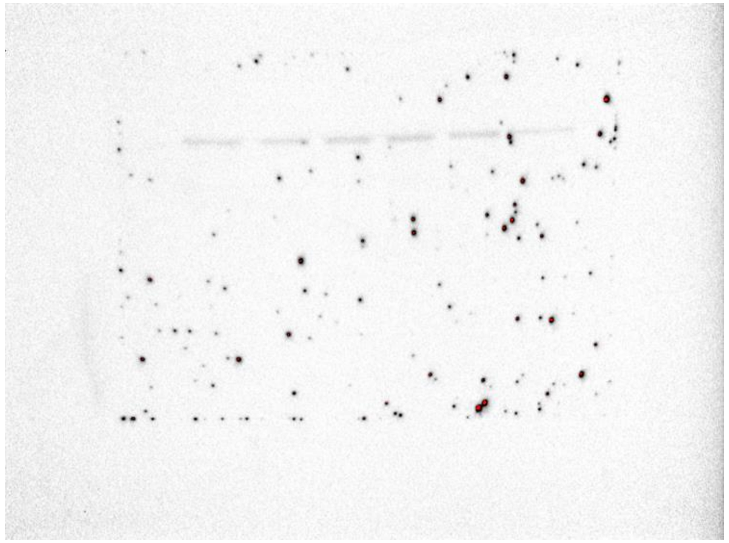

3

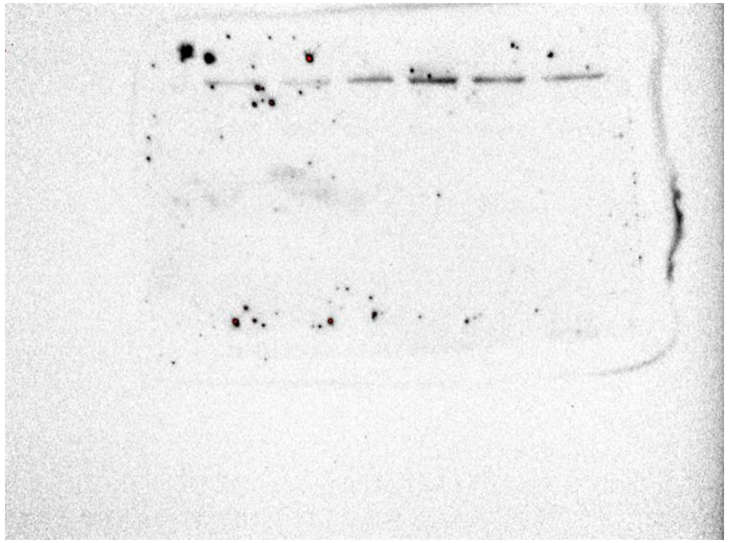

4

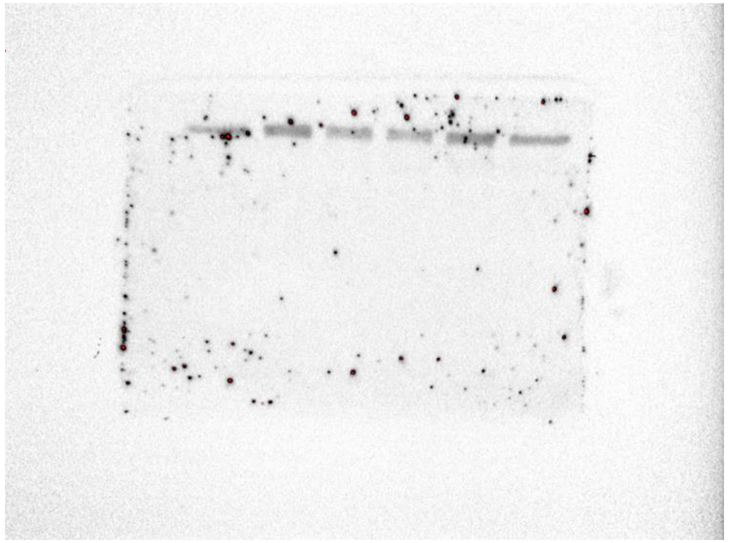

5

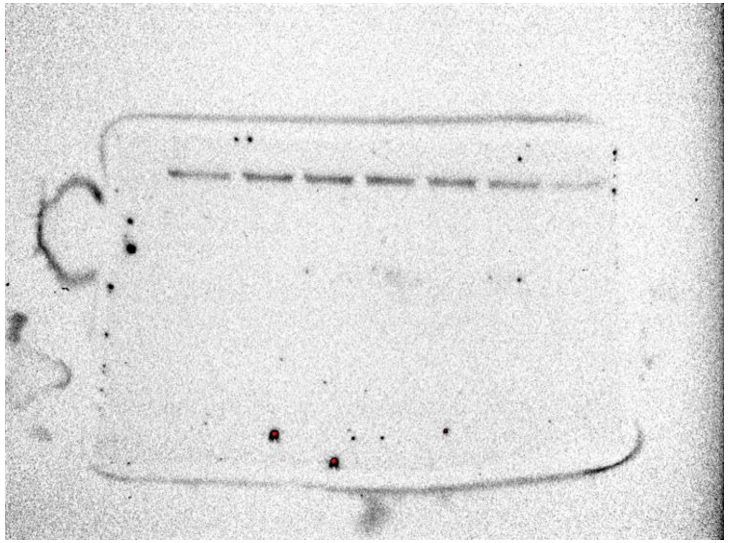

6

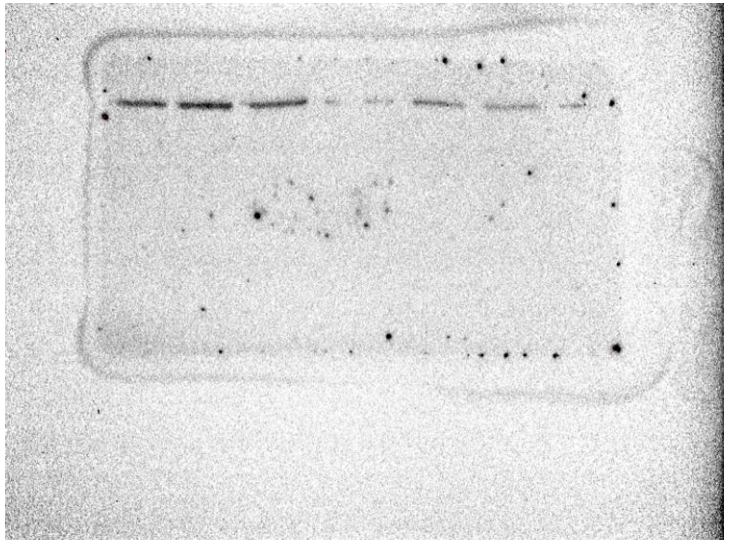

ATG5 - Ponceau S

This study

Samples order from the molecular weight marker: **MTX | DOX18 | CTRL** | DOX9-1 | CTRL-2 | DOX9-2

Other study

1

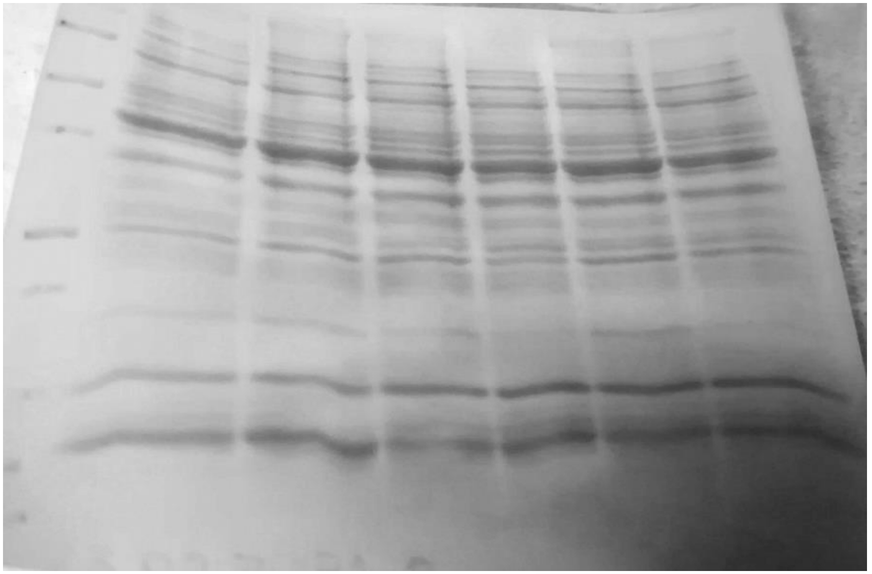

2

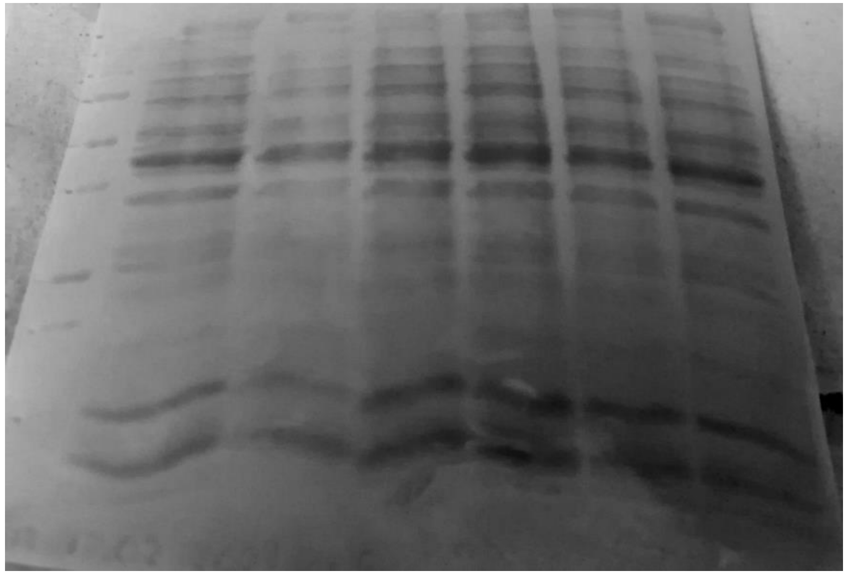

3

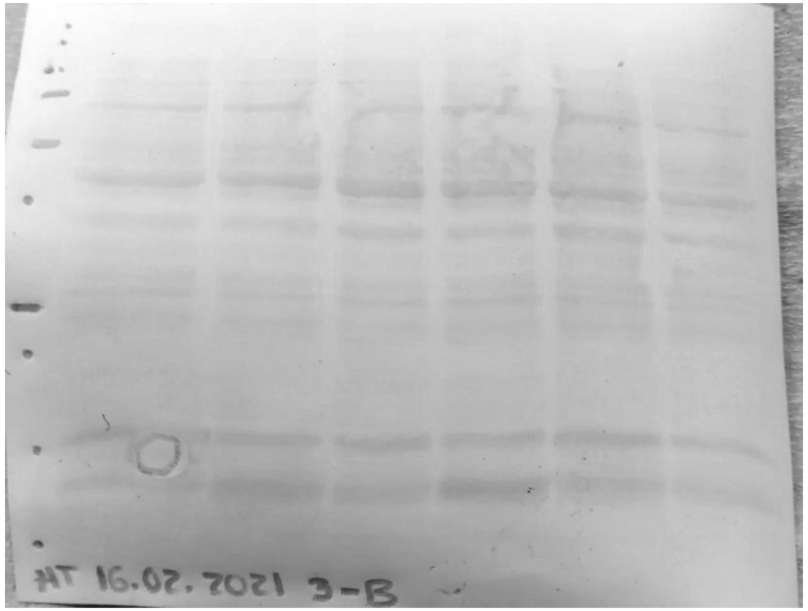

4

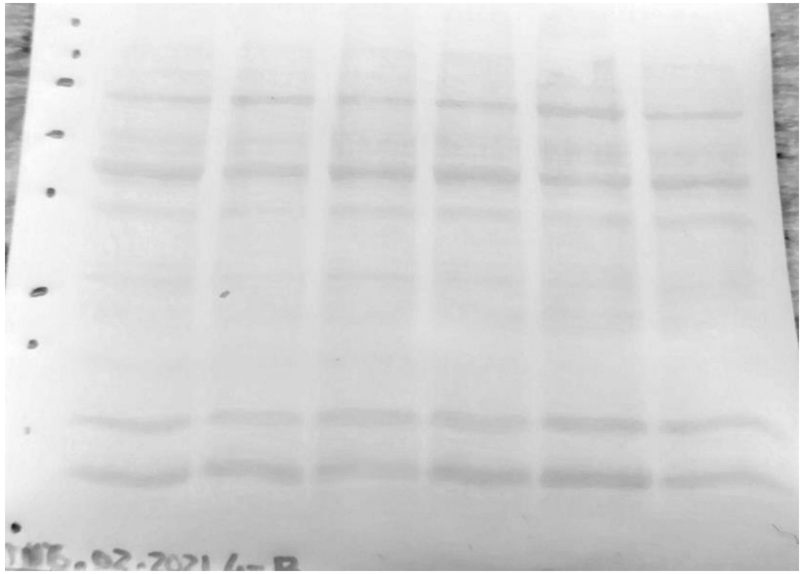

5

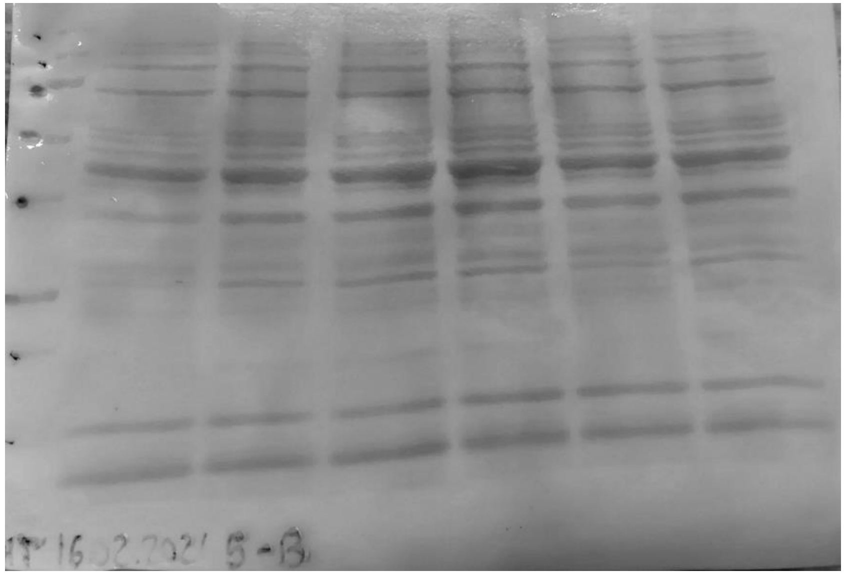

6

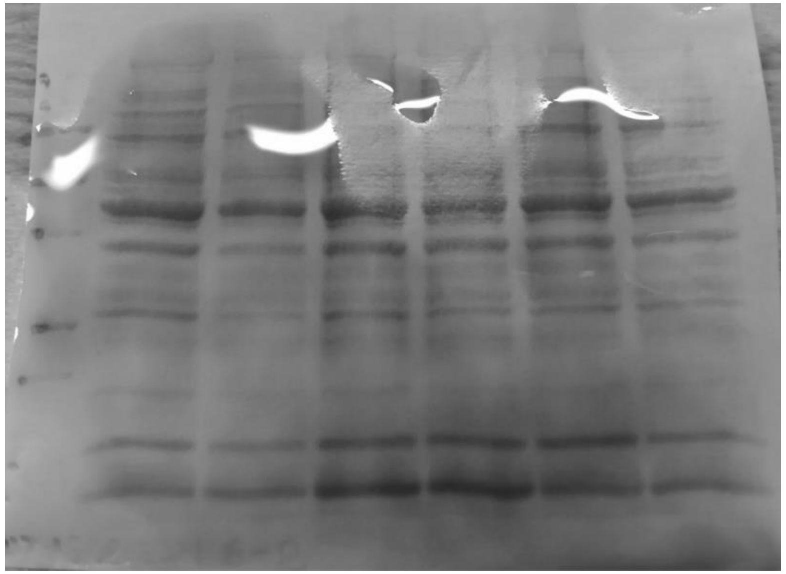

ATG5 - Blot

This study

Samples order from the molecular weight marker: **MTX | DOX18 | CTRL** | DOX9-1 | CTRL-2 | DOX9-2

Other study

1

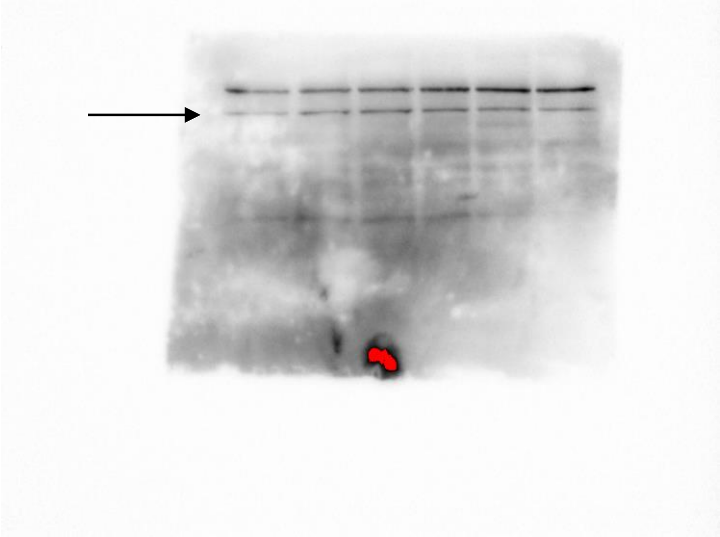

2

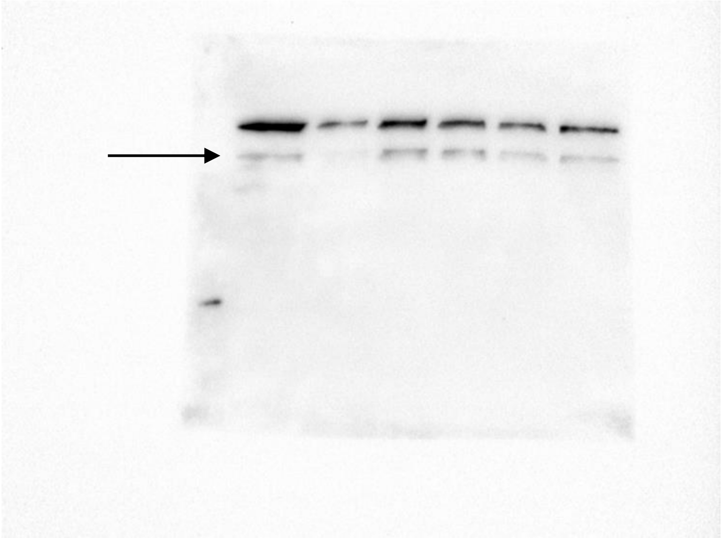

3

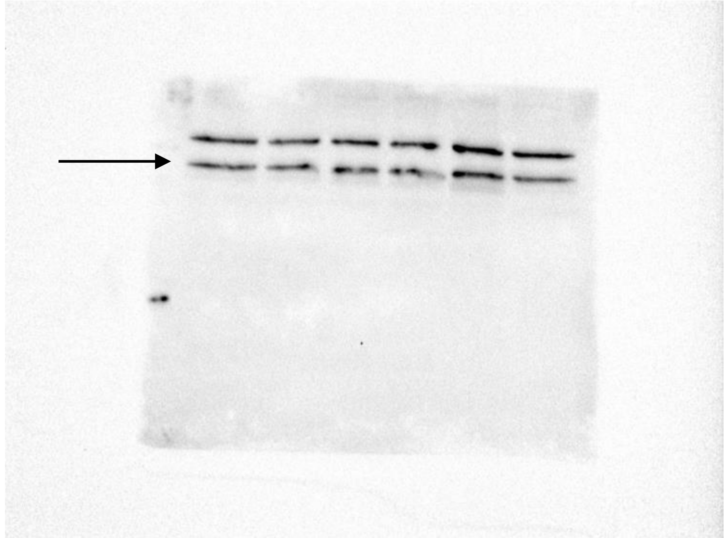

4

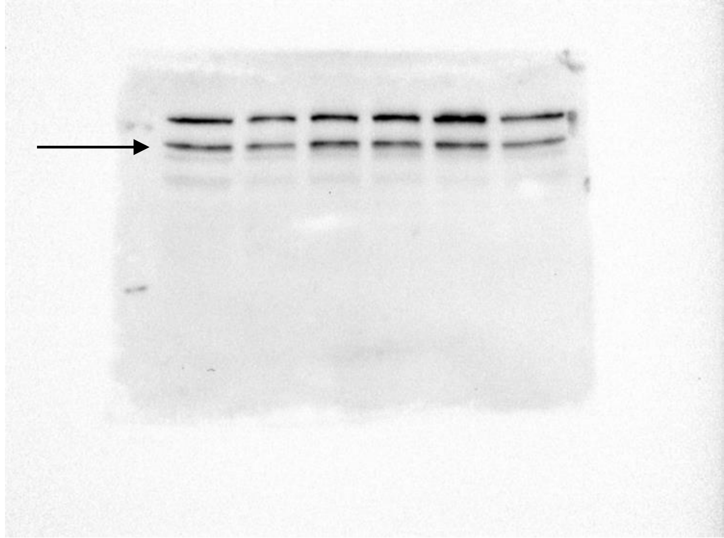

5

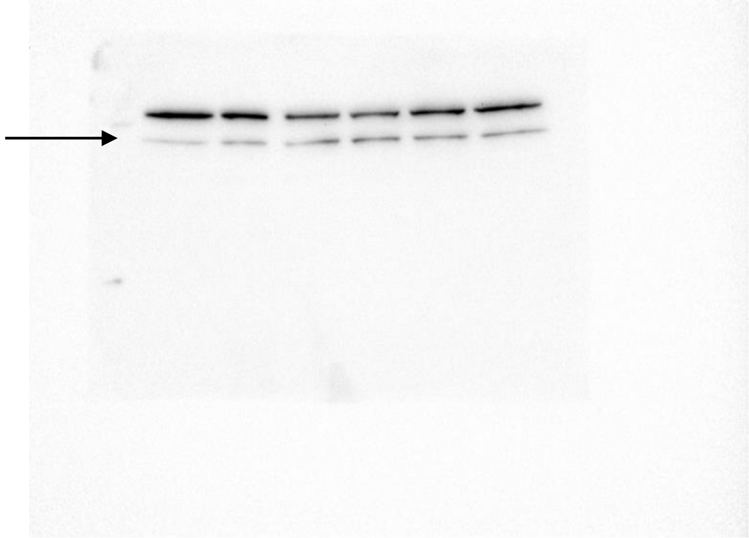

6

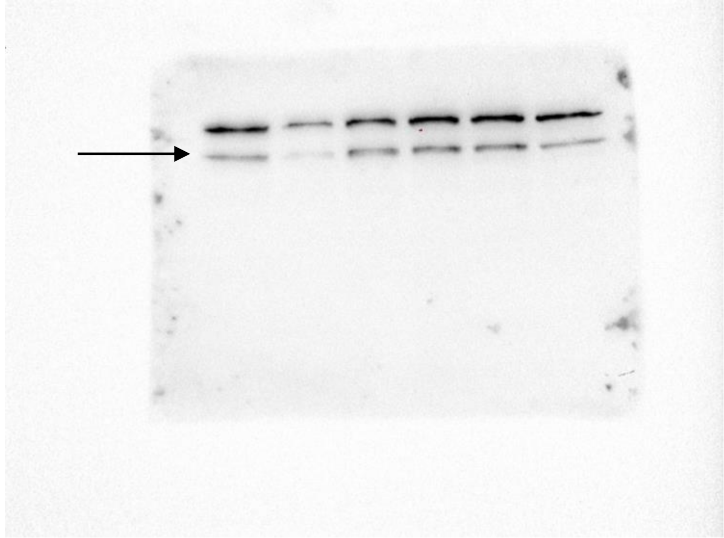

LC3B - Ponceau S

This study

Samples order from the molecular weight marker: **MTX | DOX18 | CTRL** | DOX9-1 | CTRL-2 | DOX9-2

Other study

1

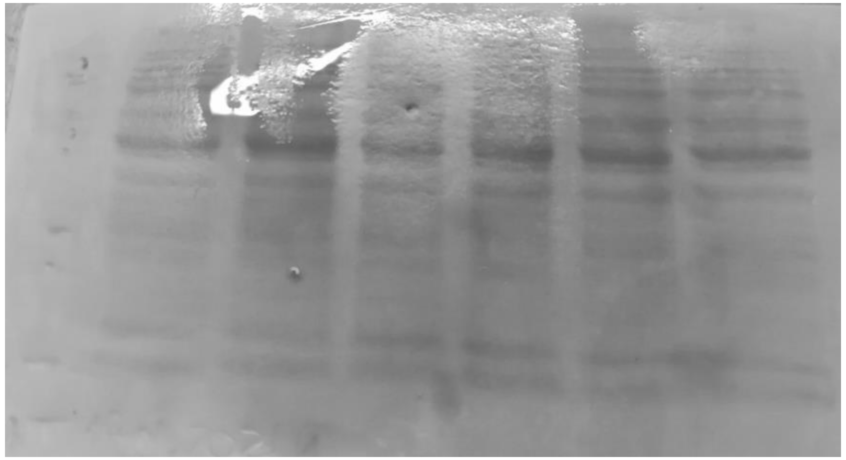

2

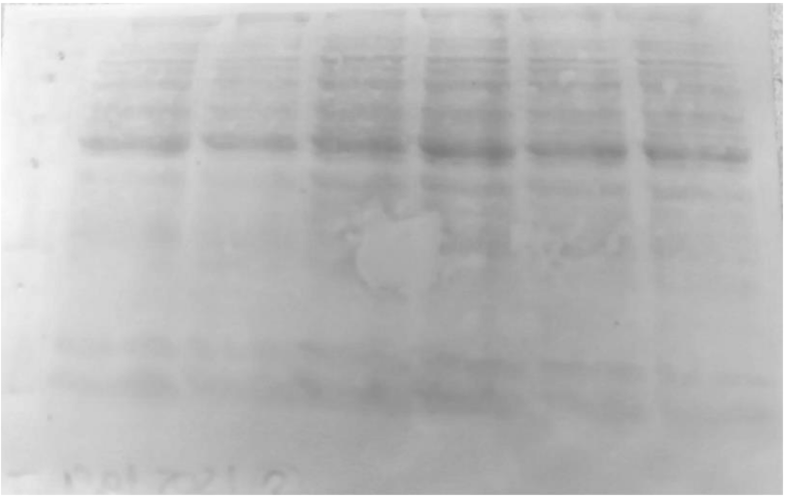

3

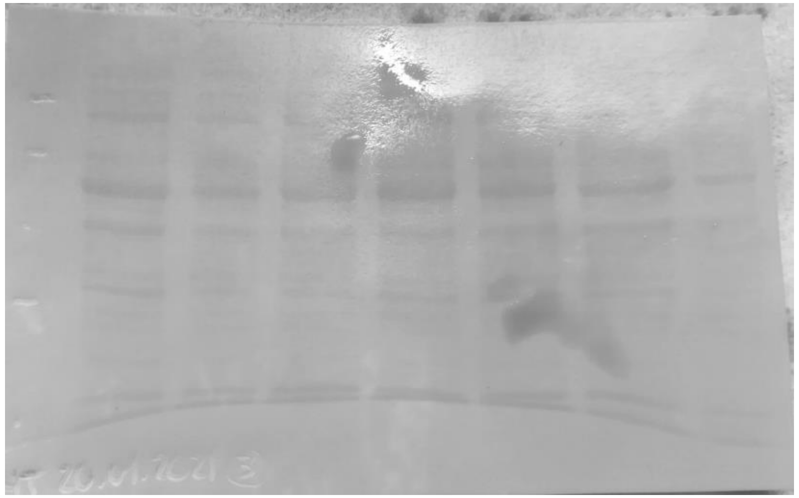

4

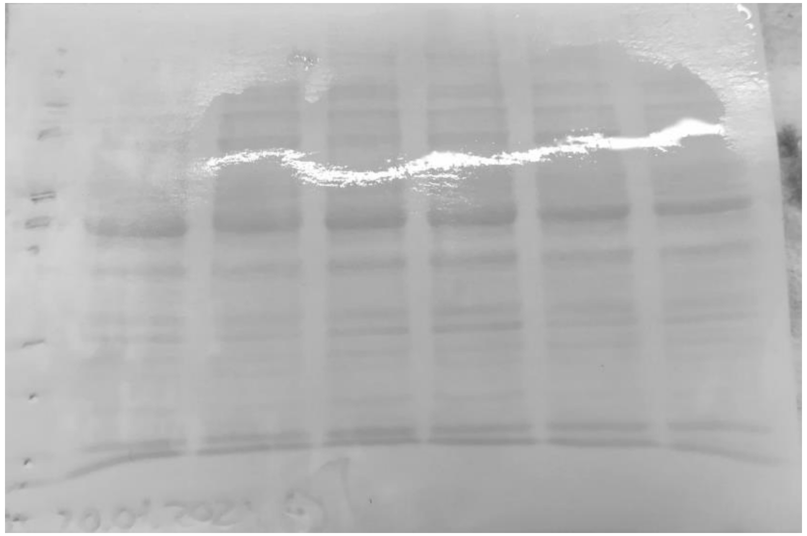

5

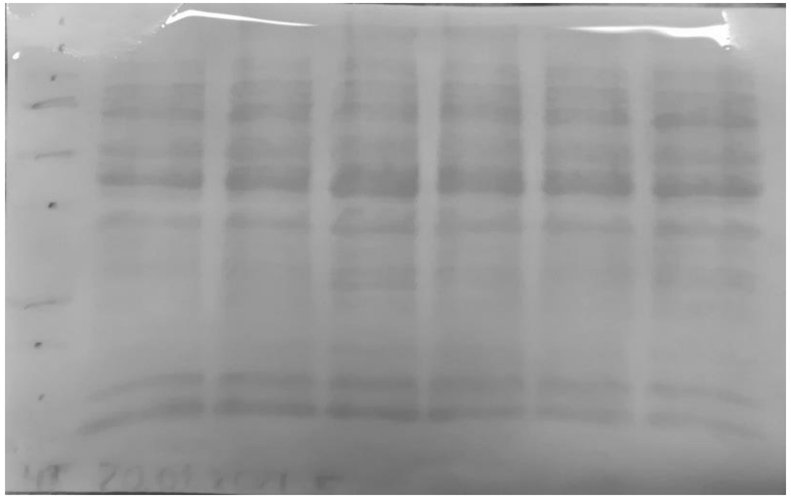

6

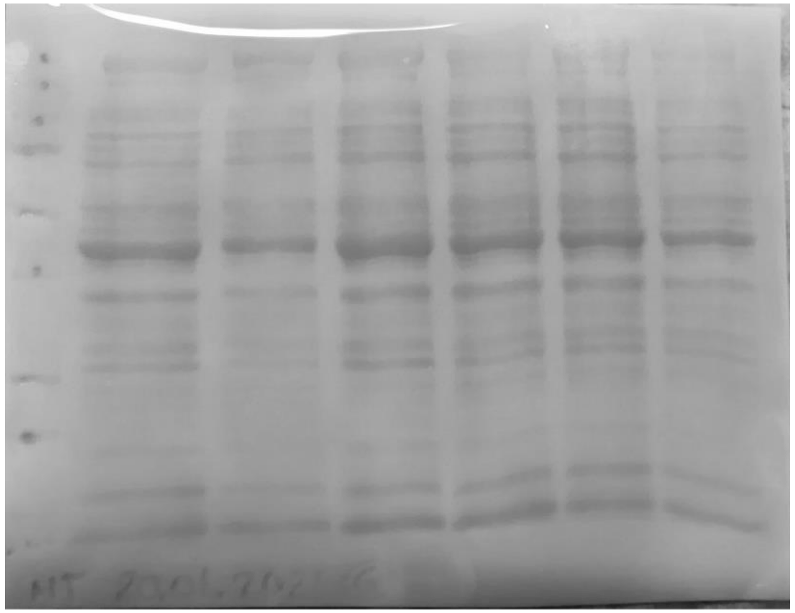

LC3B - Blot

This study

Samples order from the molecular weight marker: **MTX | DOX18 | CTRL** | DOX9-1 | CTRL-2 | DOX9-2

Other study

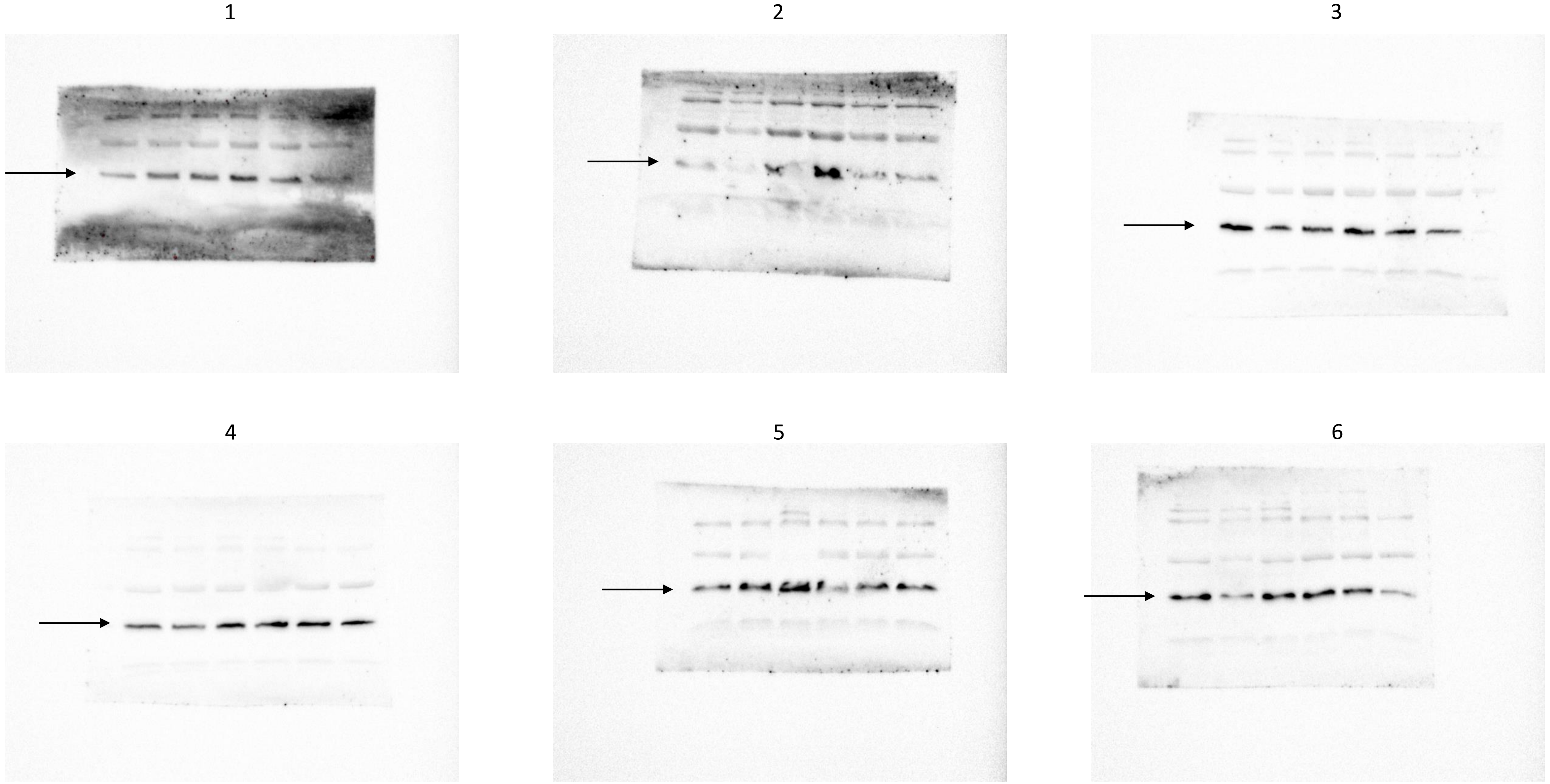

Parkin - Ponceau S

This study

Samples order from the molecular weight marker: **MTX | DOX18 | CTRL** | DOX9-1 | CTRL-2 | DOX9-2

Other study

1

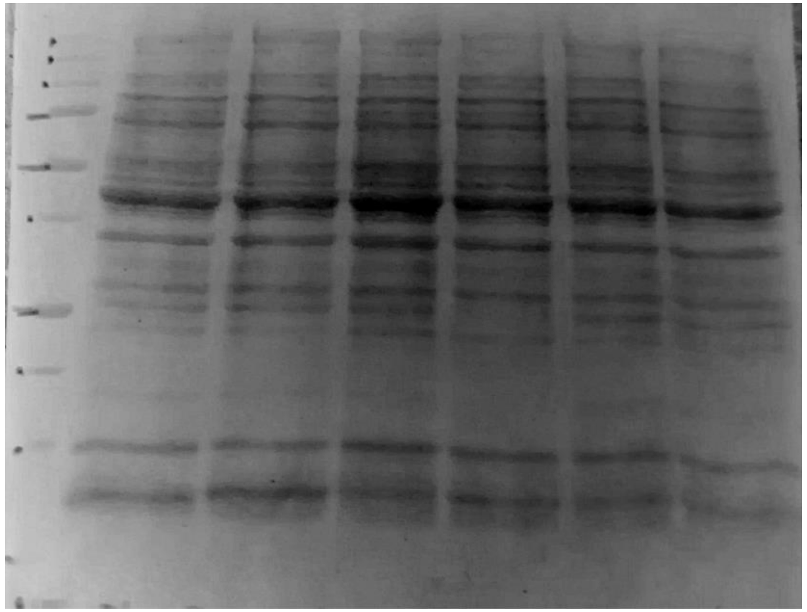

2

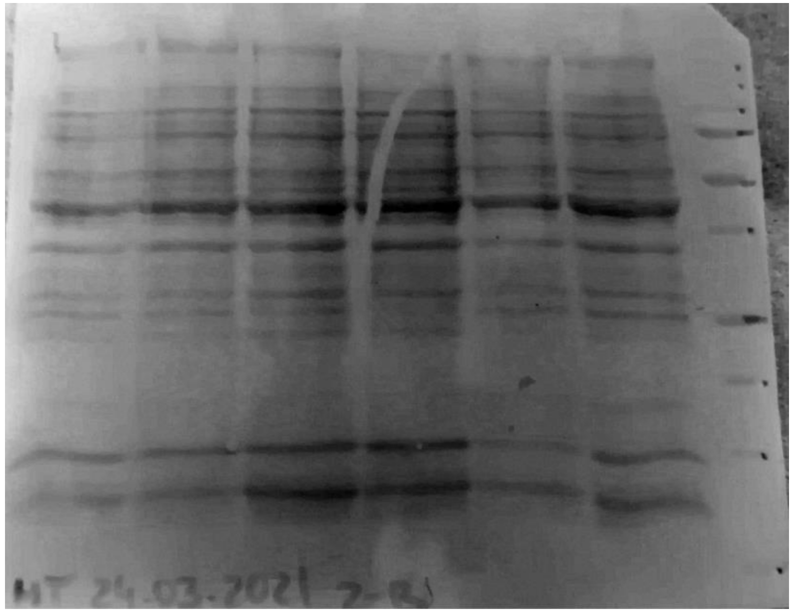

3

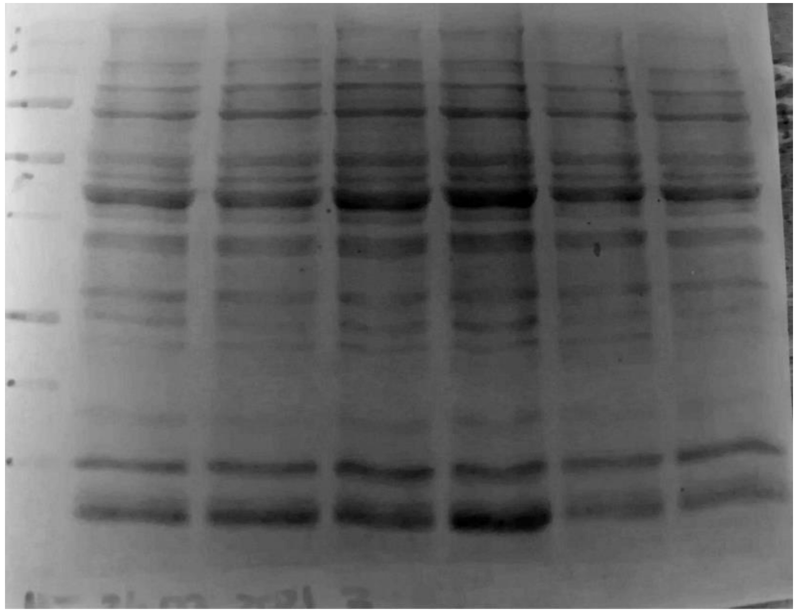

4

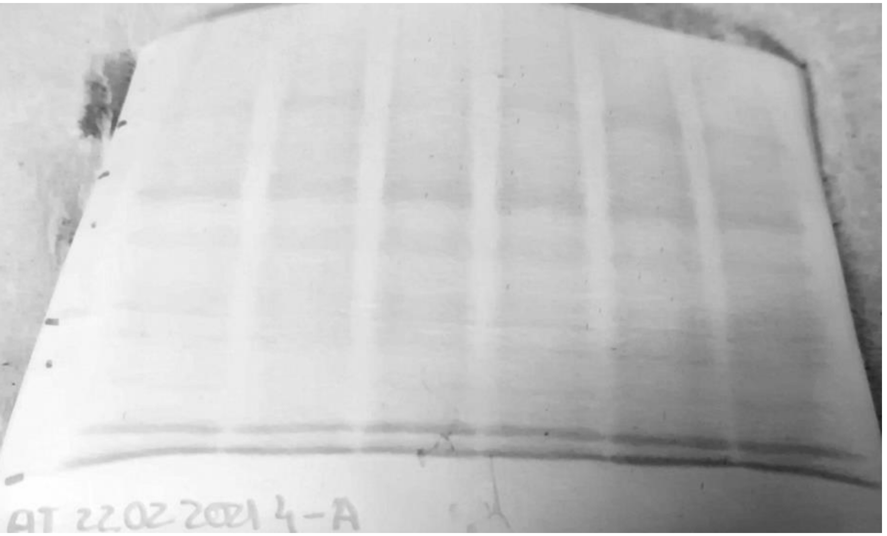

5

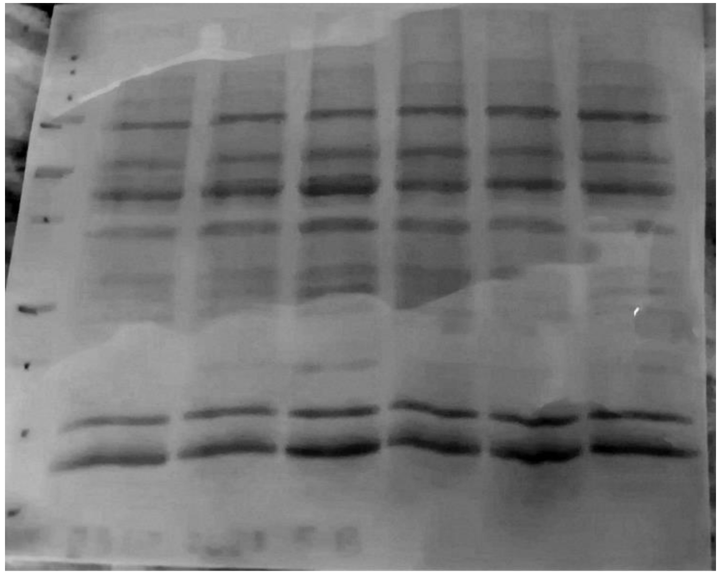

6

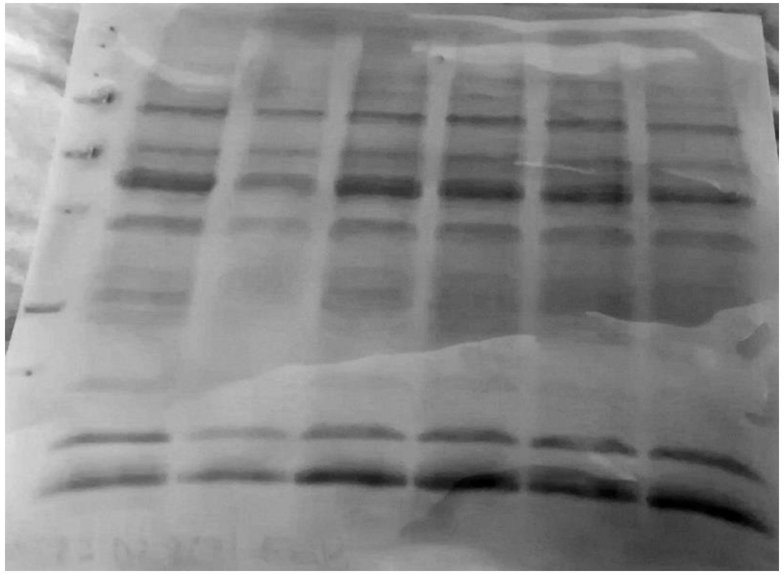

Parkin - Blot

This study

Samples order from the molecular weight marker: **MTX | DOX18 | CTRL** | DOX9-1 | CTRL-2 | DOX9-2

Other study

1

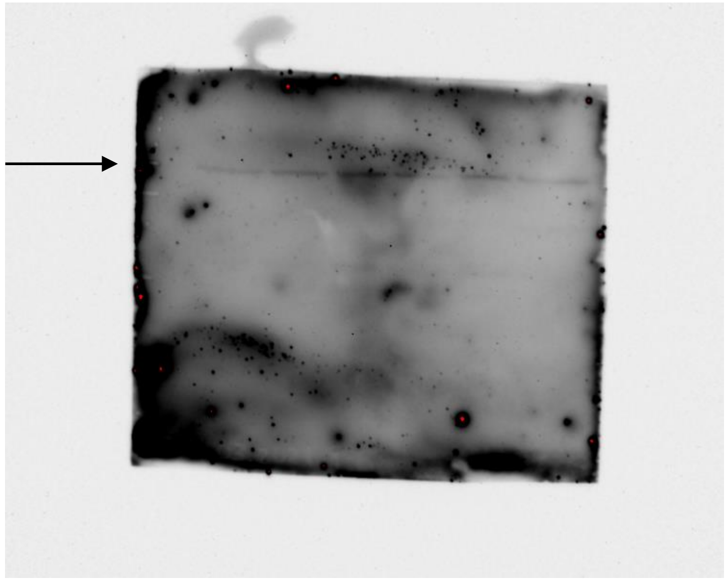

2

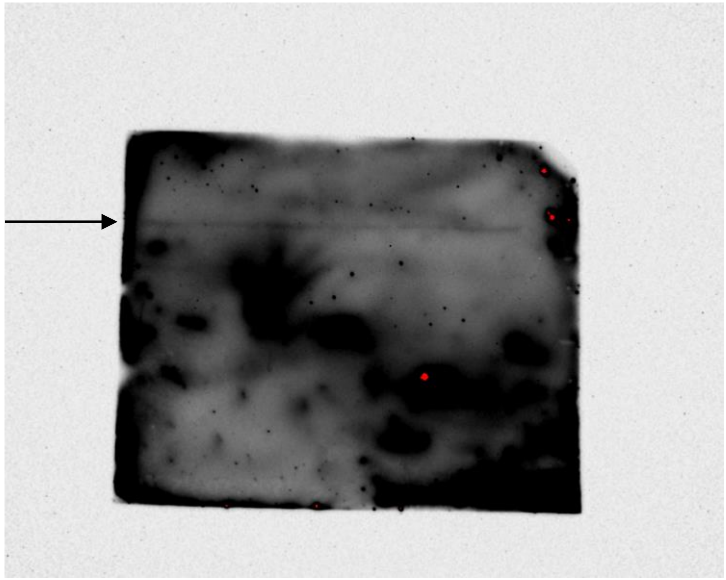

3

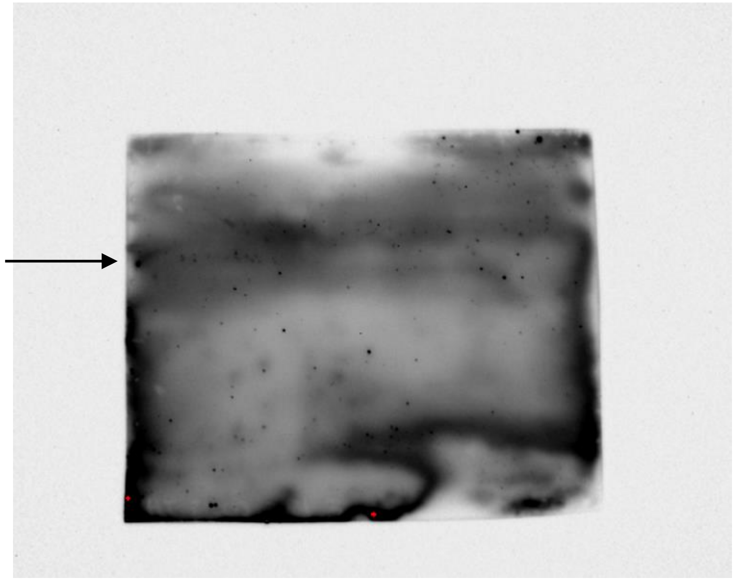

4

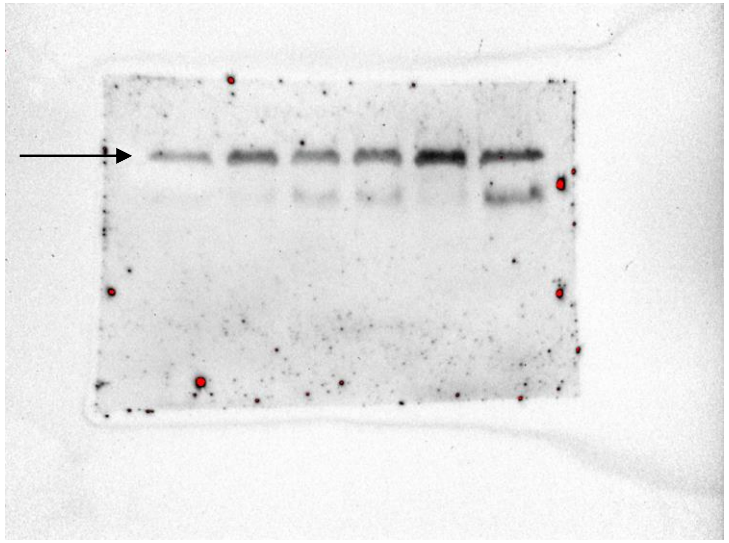

5

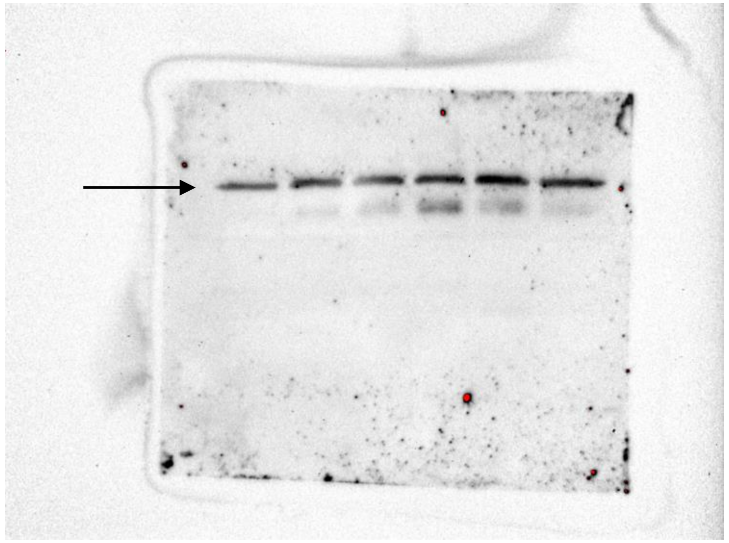

6

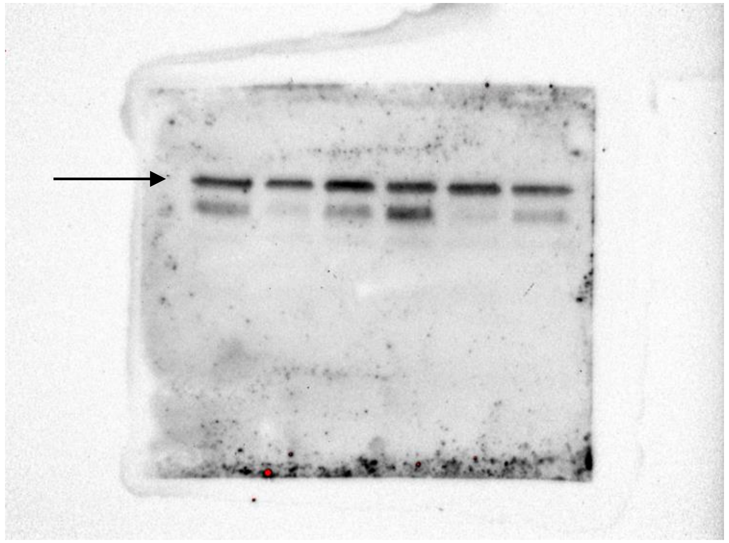

BNIP3 - Ponceau S

This study

Samples order from the molecular weight marker: **MTX | DOX18 | CTRL** | DOX9-1 | CTRL-2 | DOX9-2

Other study

1

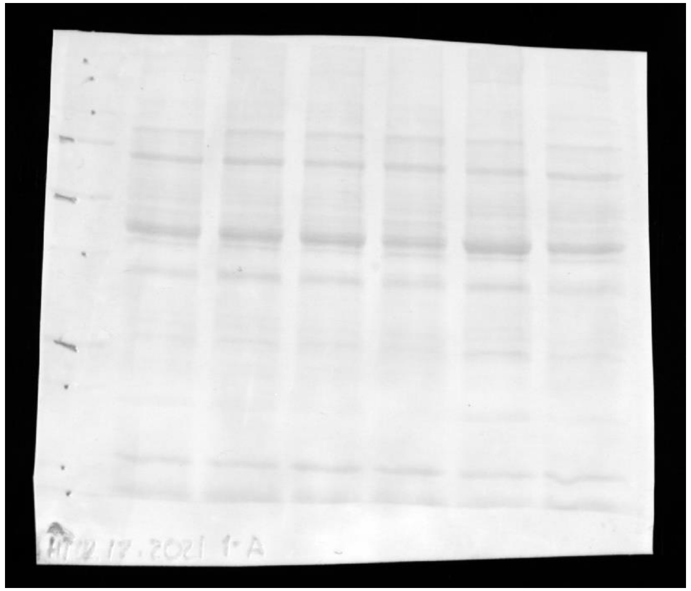

2

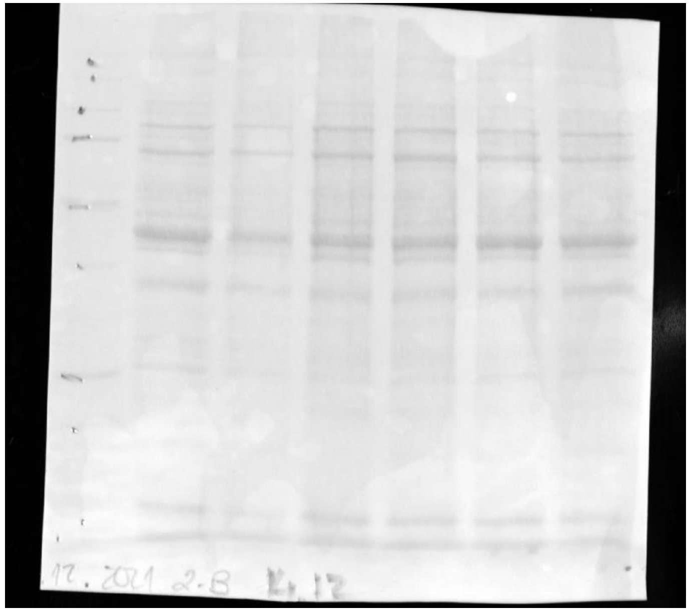

3

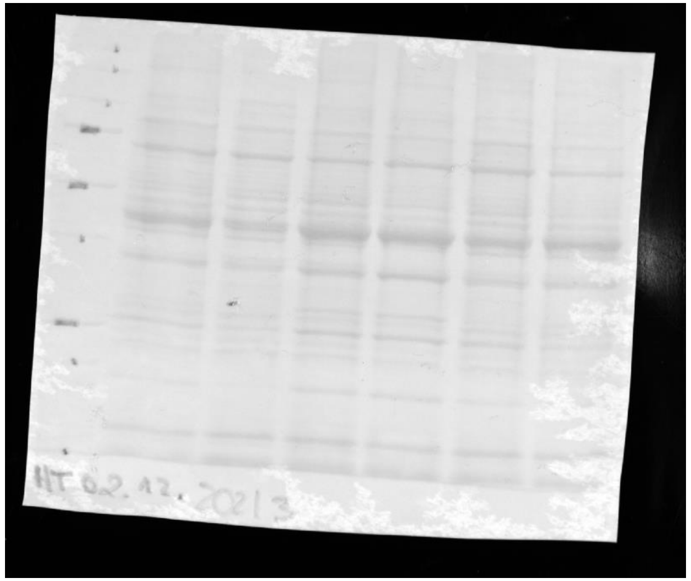

4

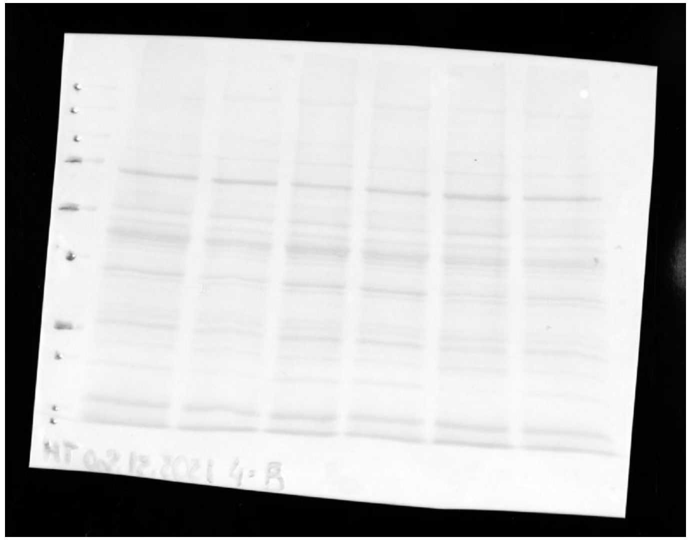

5

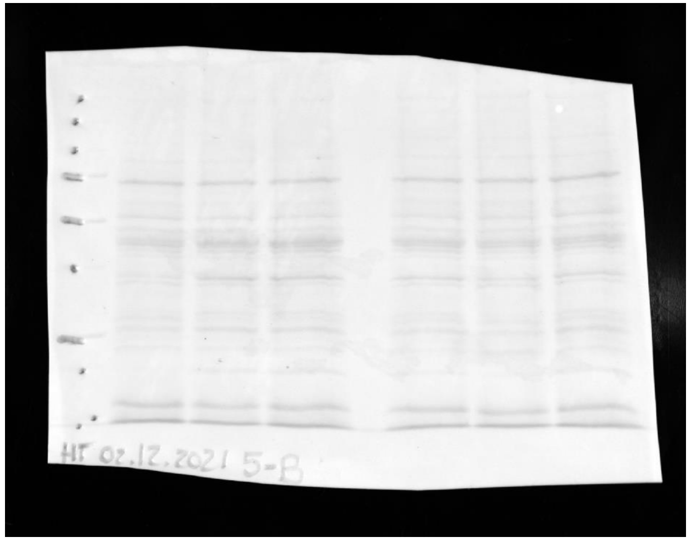

6

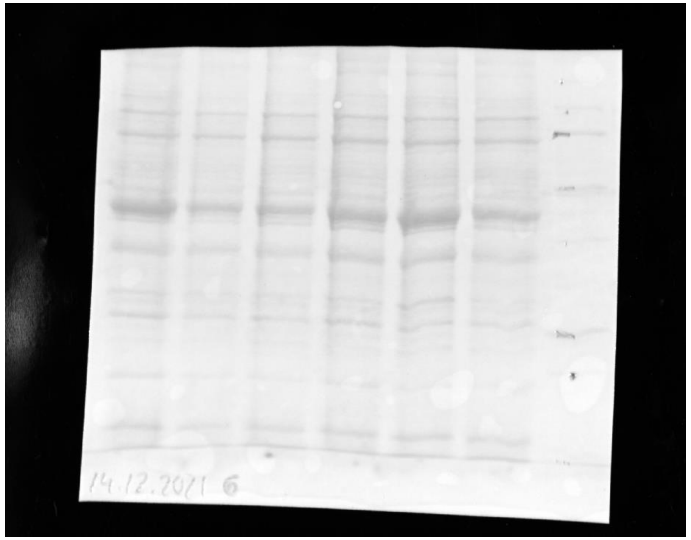

BNIP3 - Blot

This study

Samples order from the molecular weight marker: **MTX | DOX18 | CTRL** | DOX9-1 | CTRL-2 | DOX9-2

Other study

1

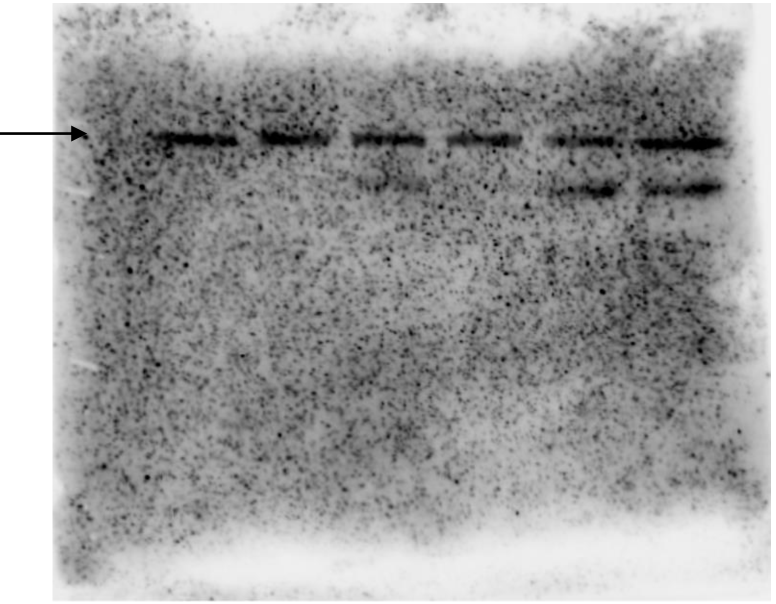

2

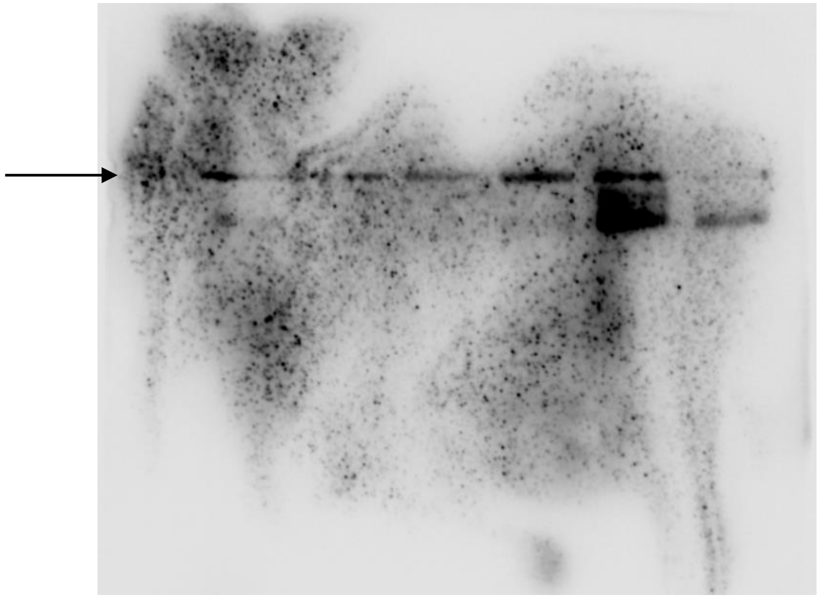

3

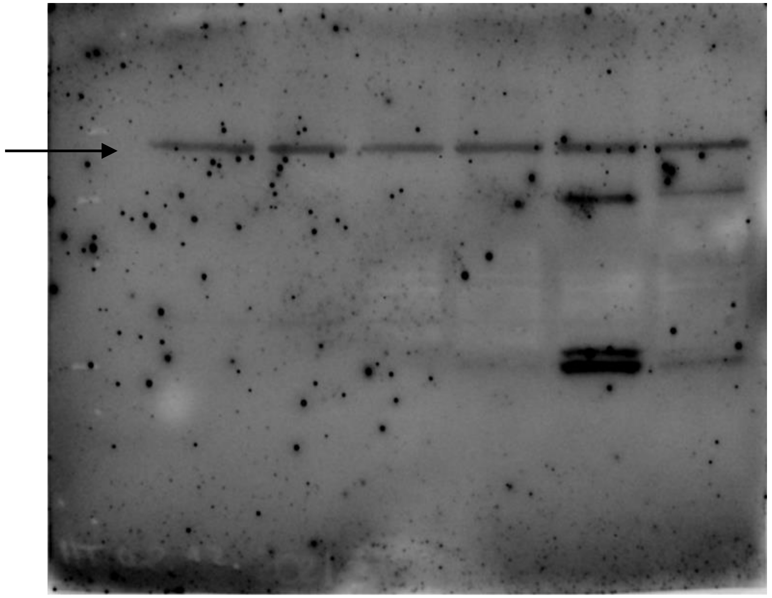

4

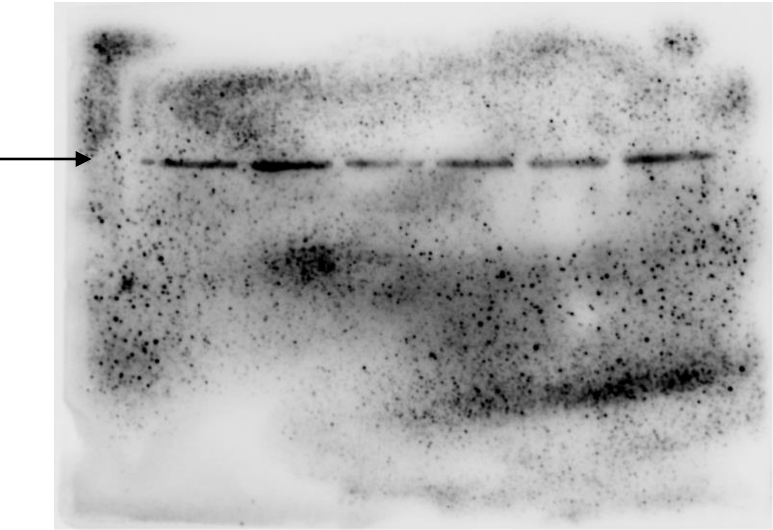

5

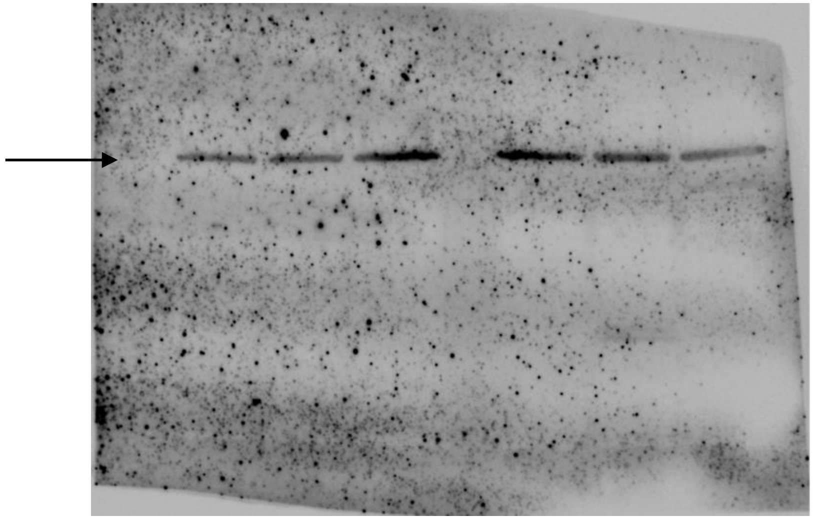

6

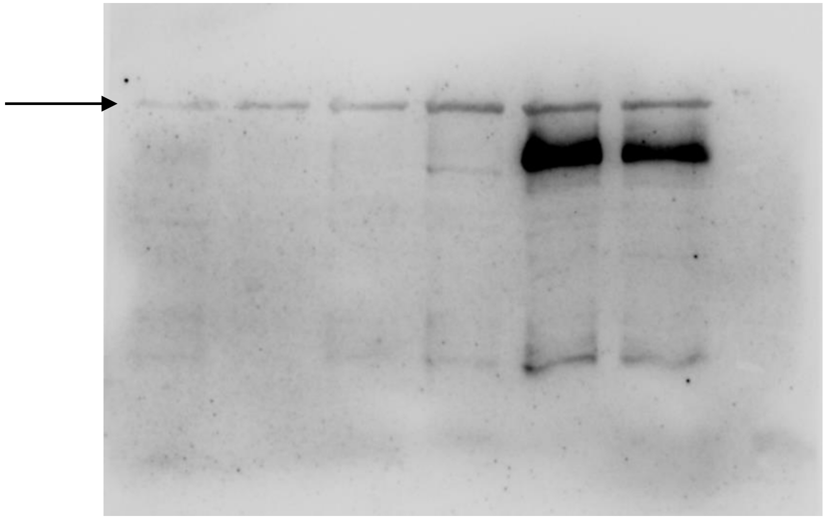

BAX - Ponceau S

This study

Samples order from the molecular weight marker: **MTX | DOX18 | CTRL** | DOX9-1 | CTRL-2 | DOX9-2

Other study

1

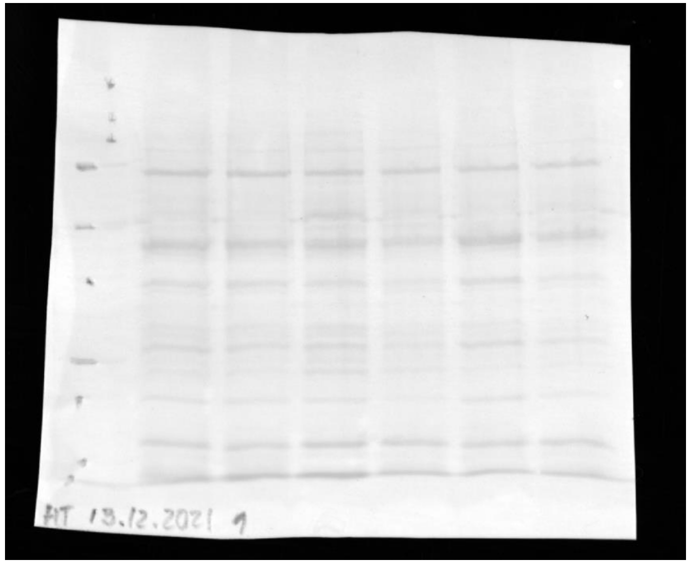

2

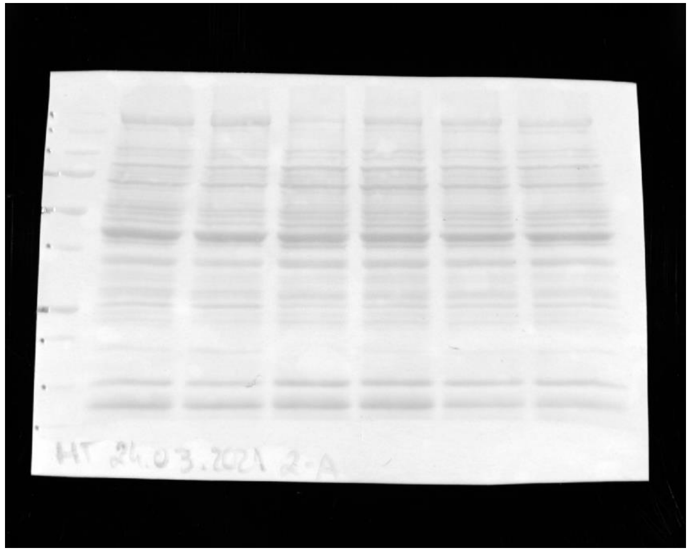

3

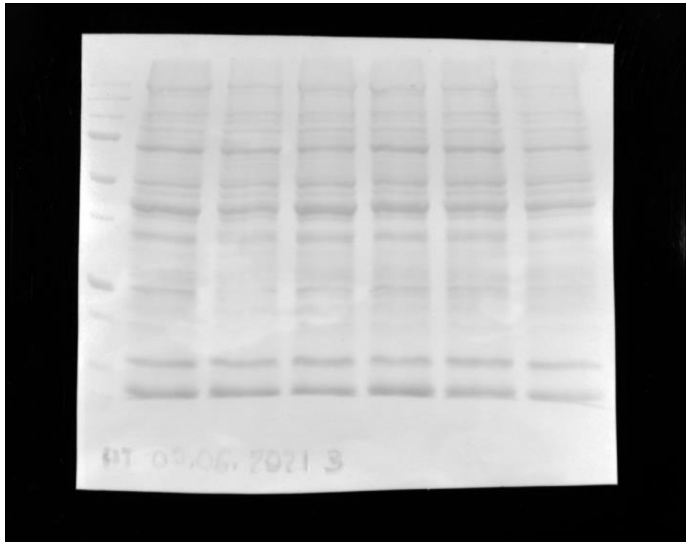

4

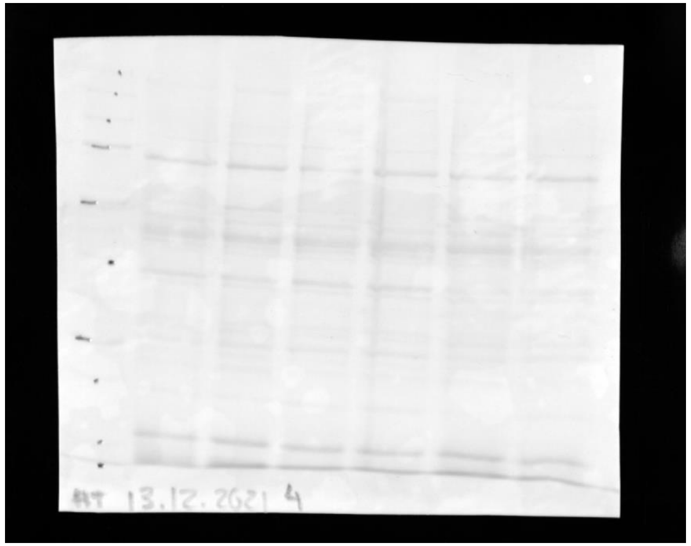

5

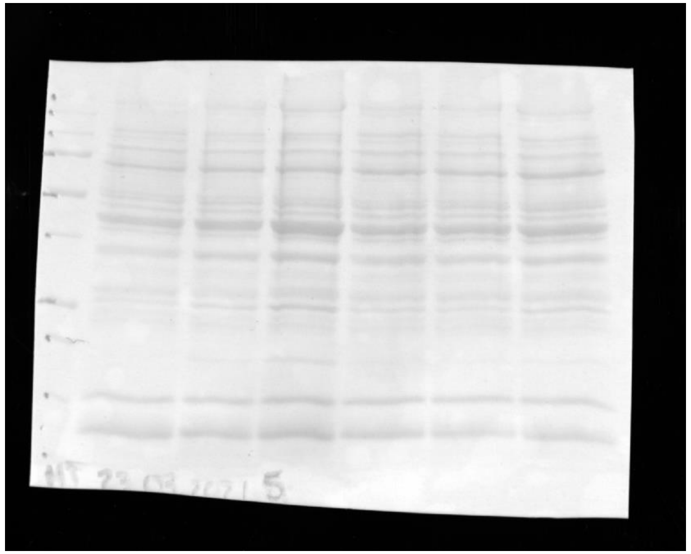

6

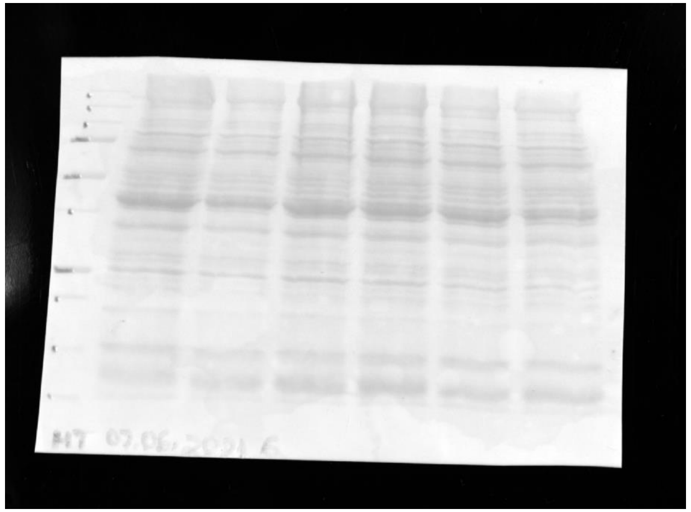

BAX - Blot

This study

Samples order from the molecular weight marker: **MTX | DOX18 | CTRL** | DOX9-1 | CTRL-2 | DOX9-2

Other study

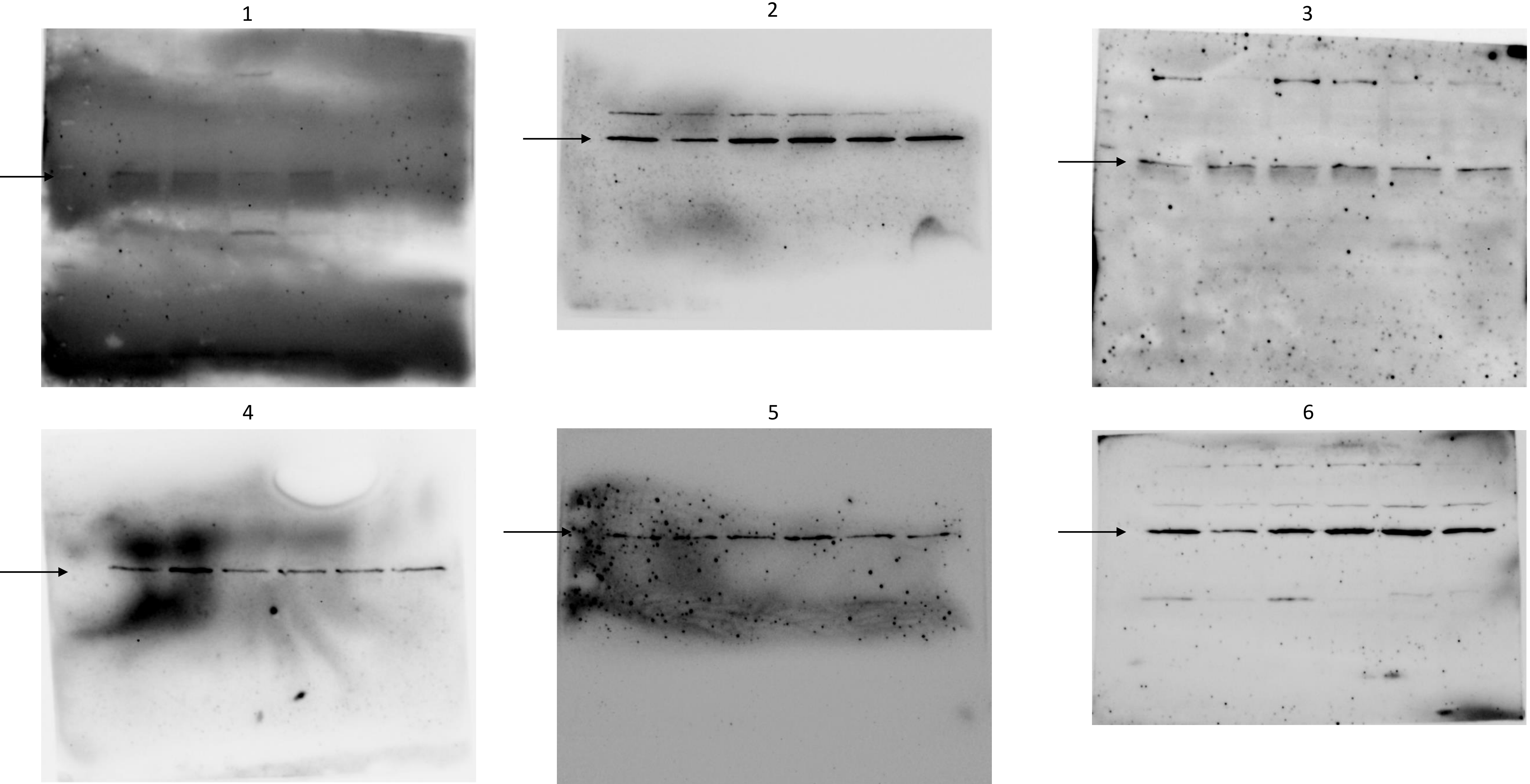

BCL2 - Ponceau S

This study

Samples order from the molecular weight marker: **MTX | DOX18 | CTRL** | DOX9-1 | CTRL-2 | DOX9-2

Other study

1

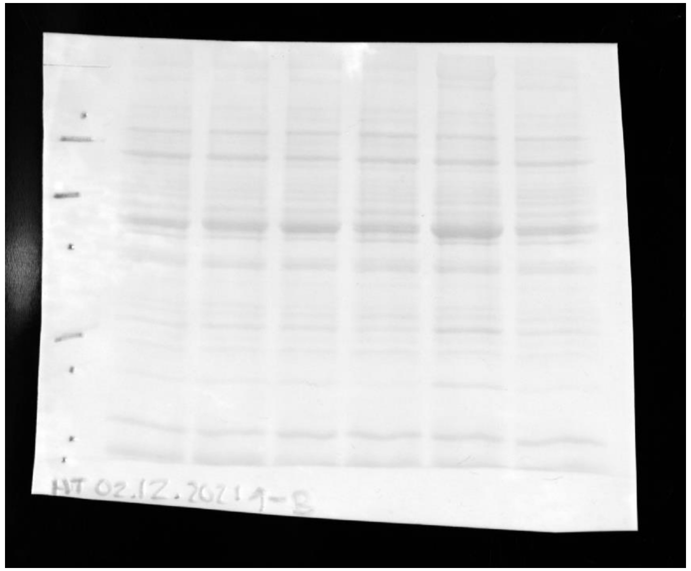

2

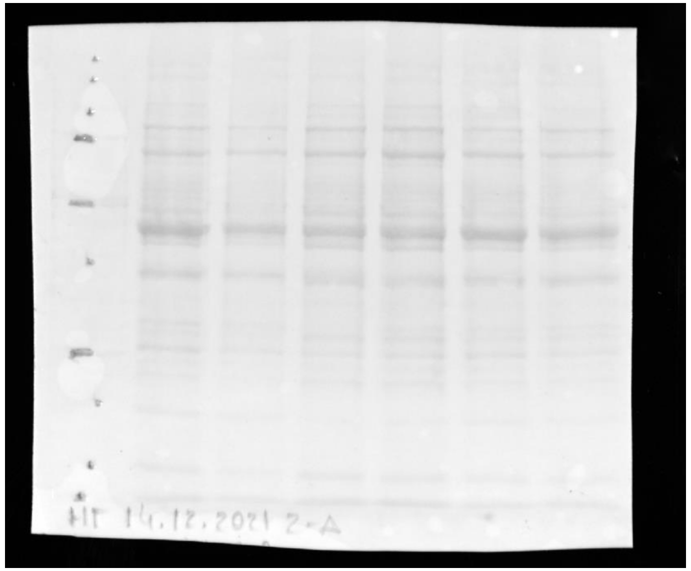

3

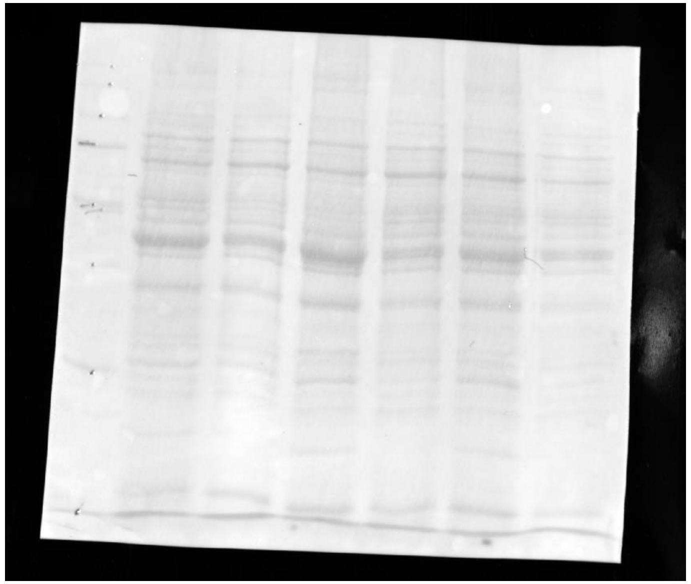

4

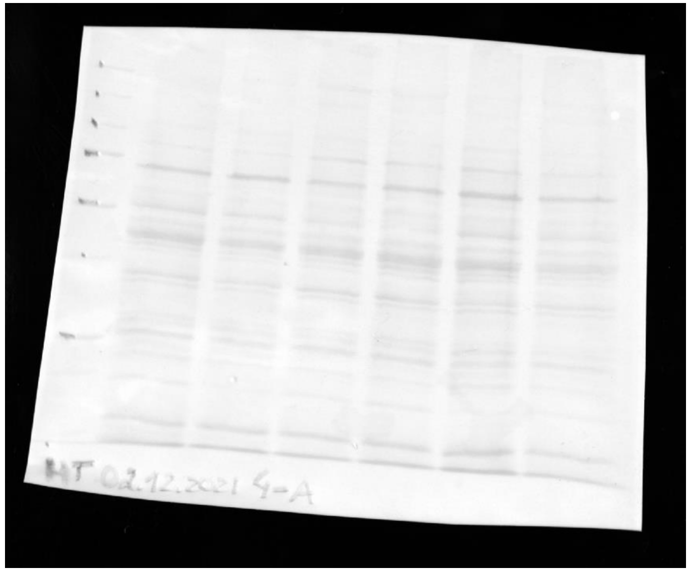

5

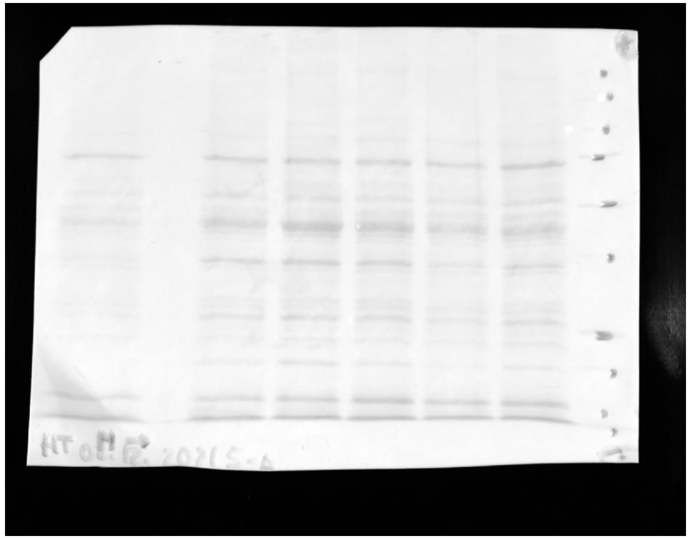

6

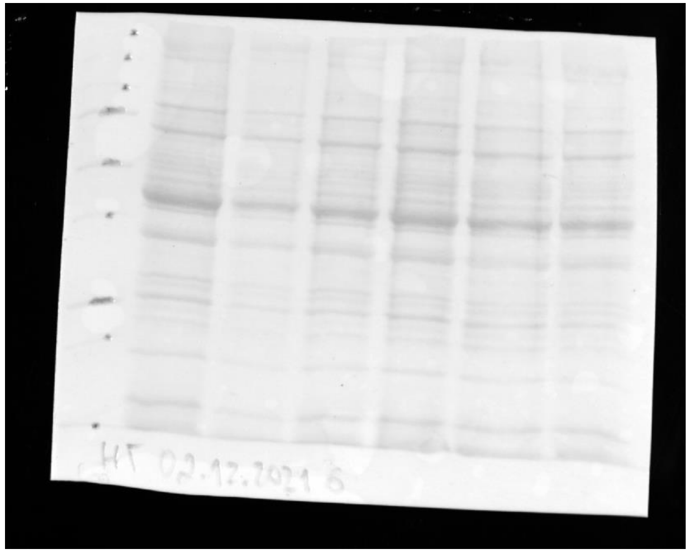

BCL2 - Blot

This study

Samples order from the molecular weight marker: **MTX | DOX18 | CTRL** | DOX9-1 | CTRL-2 | DOX9-2

Other study

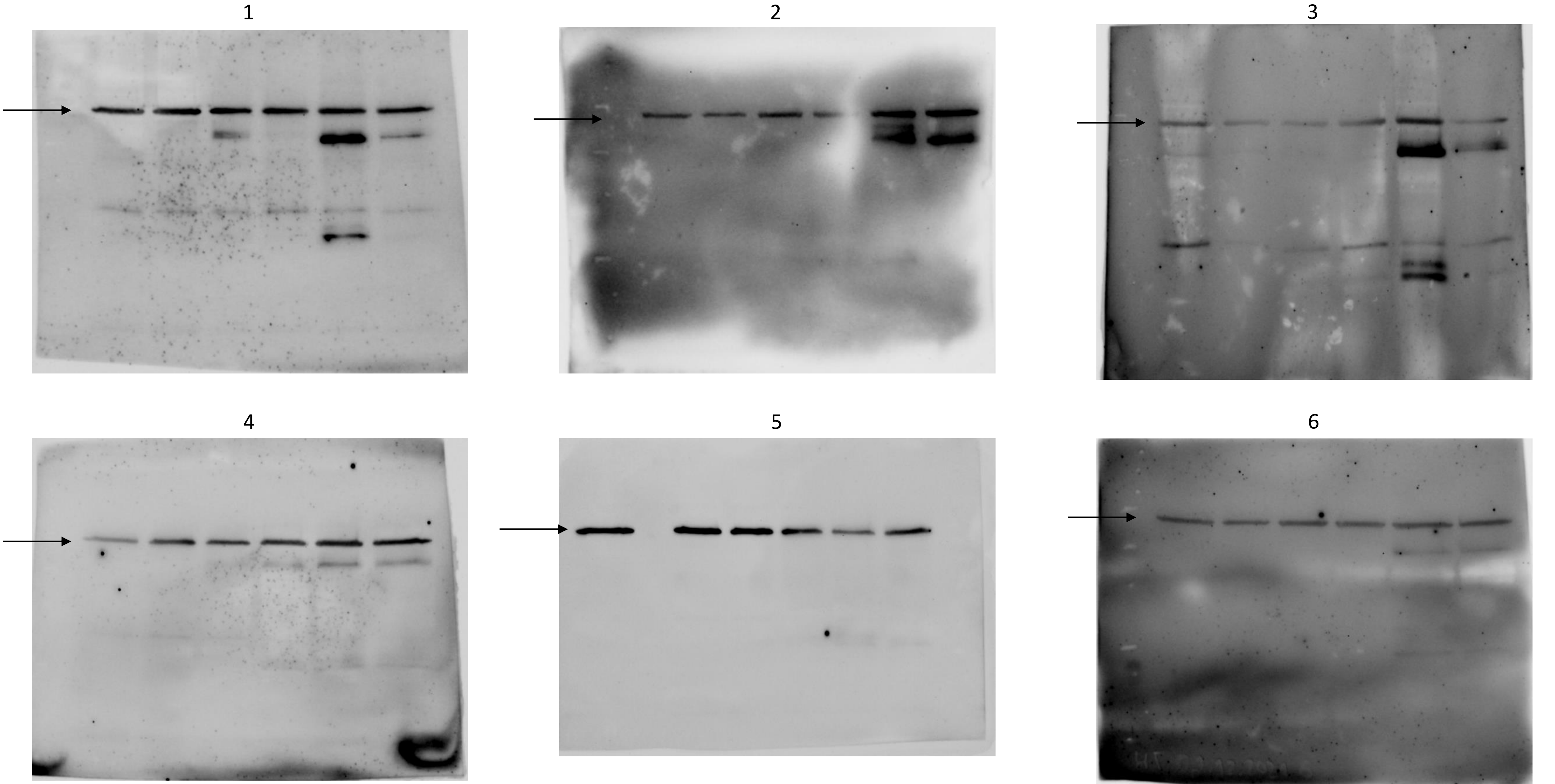

HSP27 - Ponceau S

This study

Samples order from the molecular weight marker: **MTX | DOX18 | CTRL** | DOX9-1 | CTRL-2 | DOX9-2

Other study

1

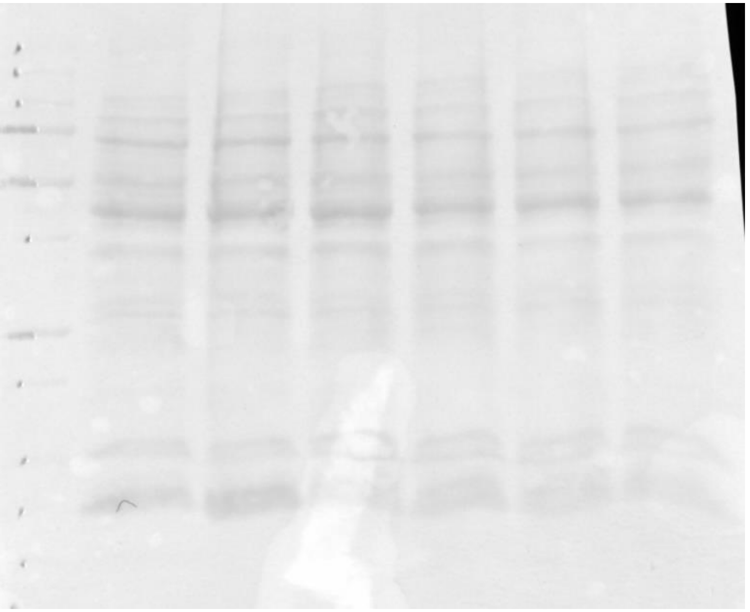

2

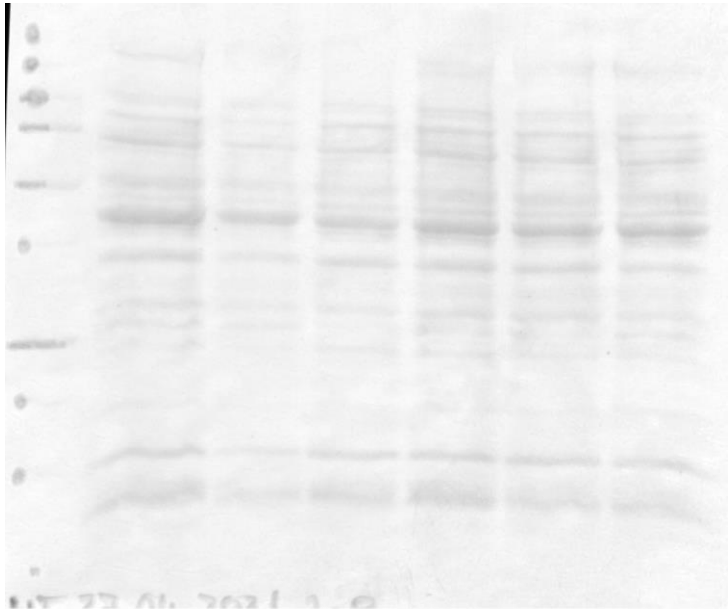

3

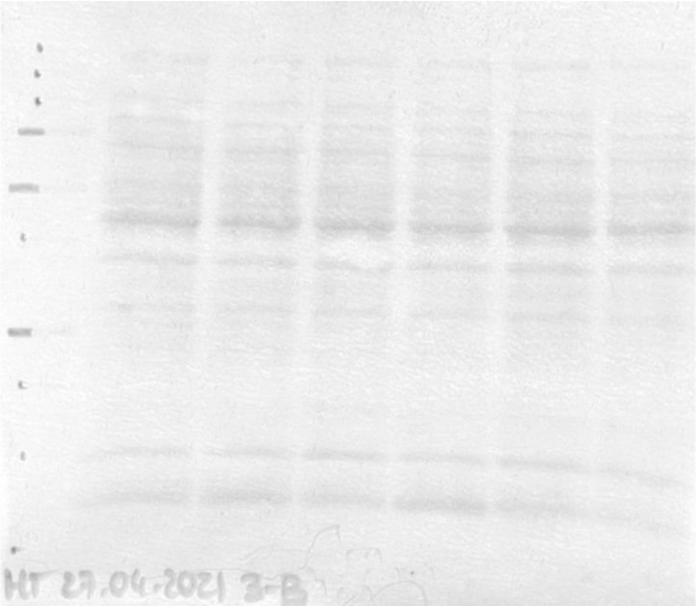

4

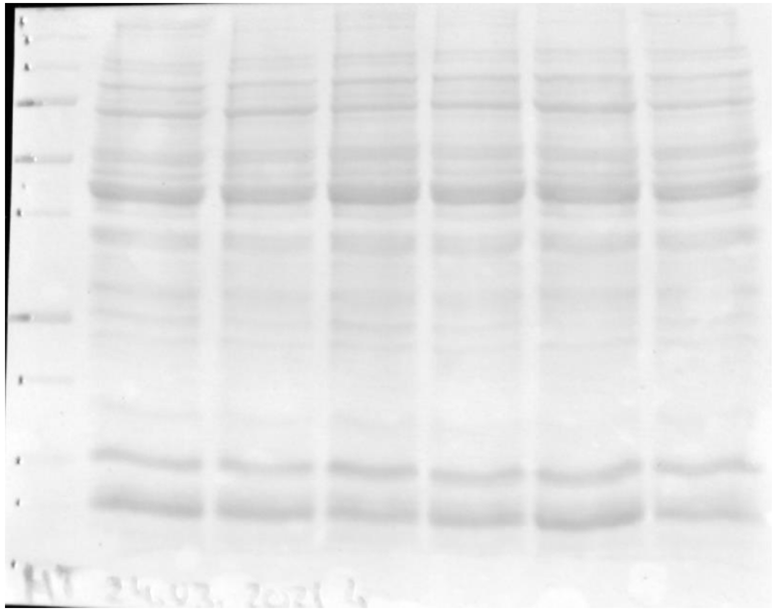

5

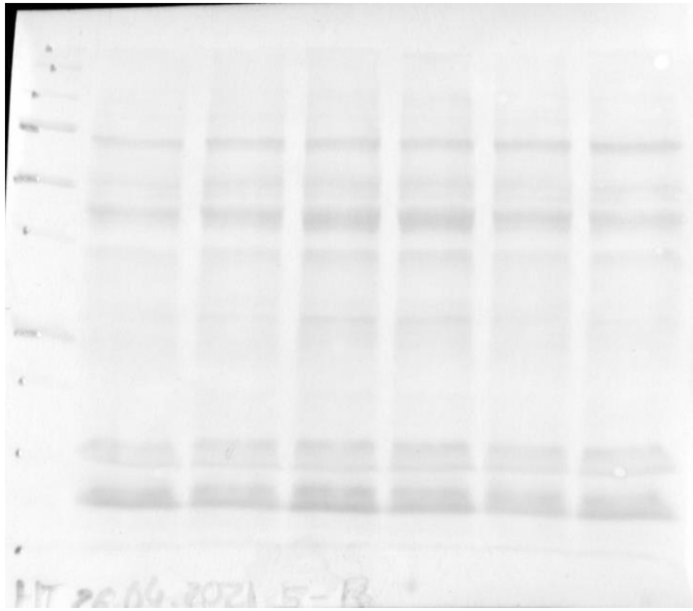

6

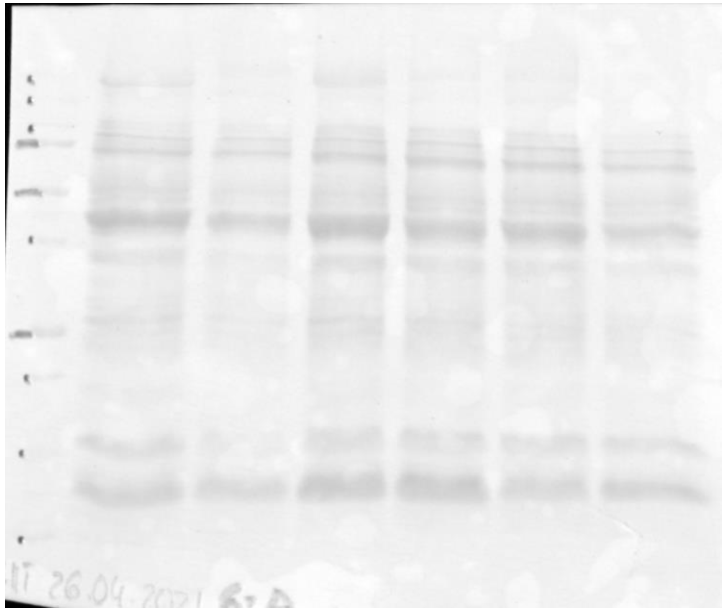

HSP27 - Blot

This study

Samples order from the molecular weight marker: **MTX | DOX18 | CTRL** | DOX9-1 | CTRL-2 | DOX9-2

Other study

1

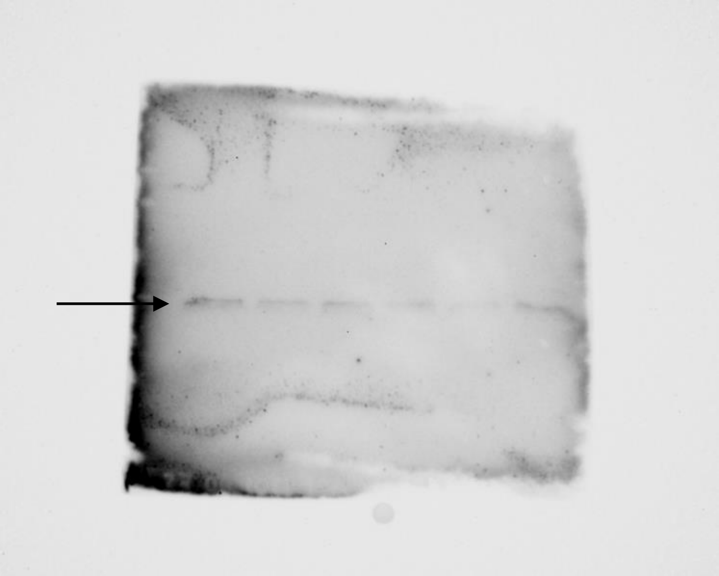

2

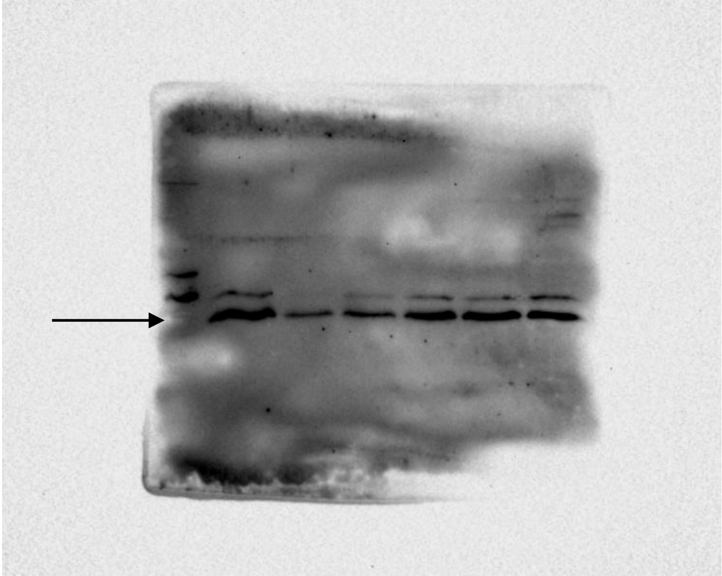

3

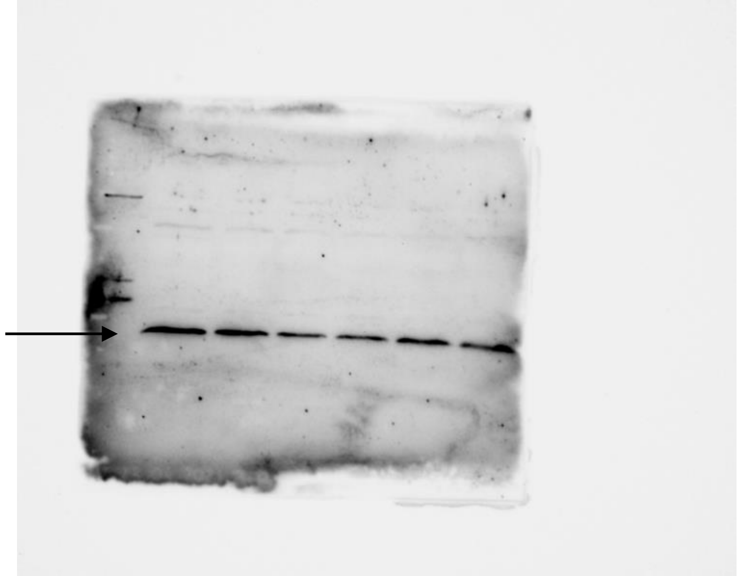

4

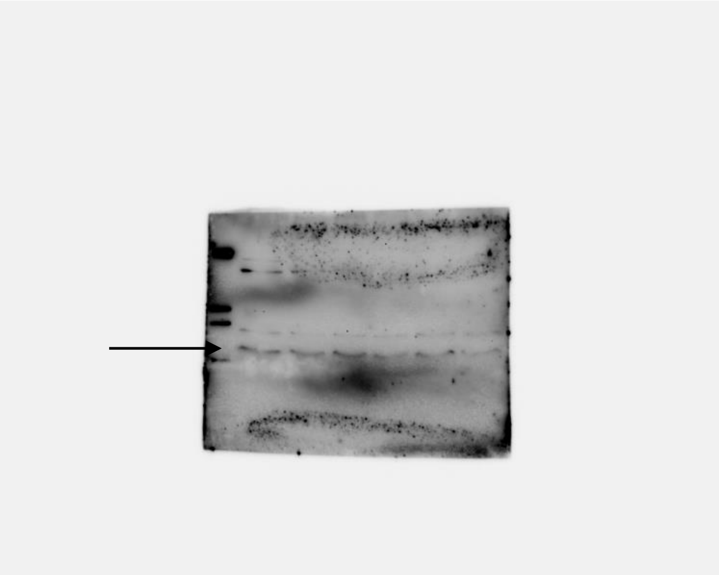

5

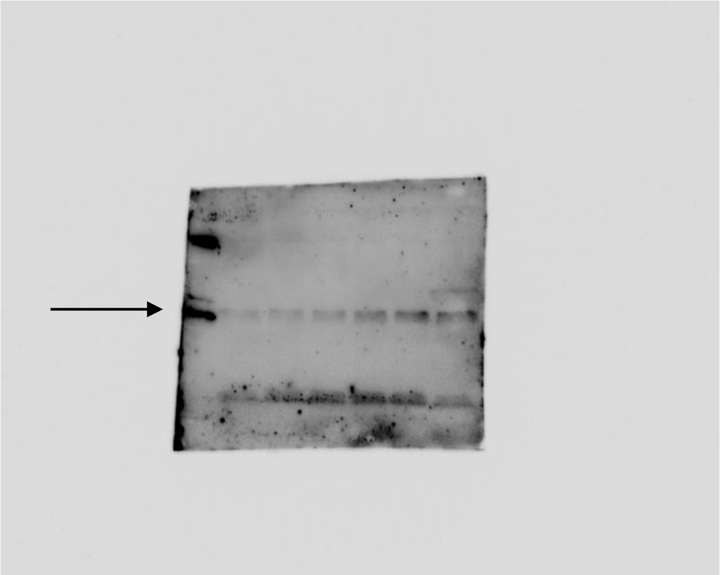

6

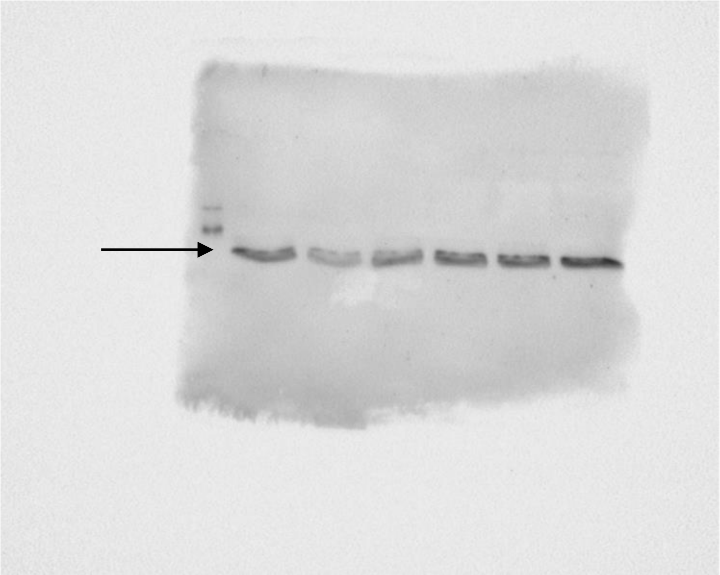

HSP70 - Ponceau S

This study

Samples order from the molecular weight marker: **MTX | DOX18 | CTRL** | DOX9-1 | CTRL-2 | DOX9-2

Other study

1

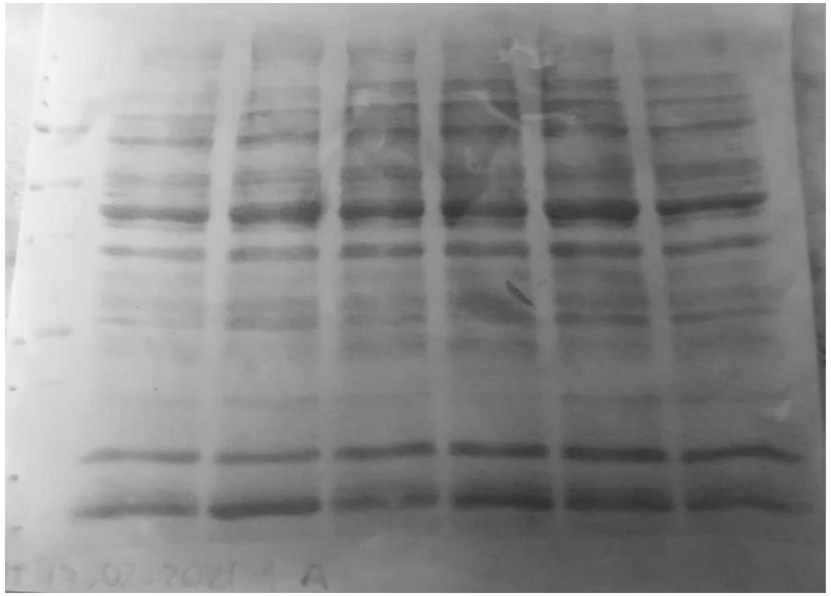

2

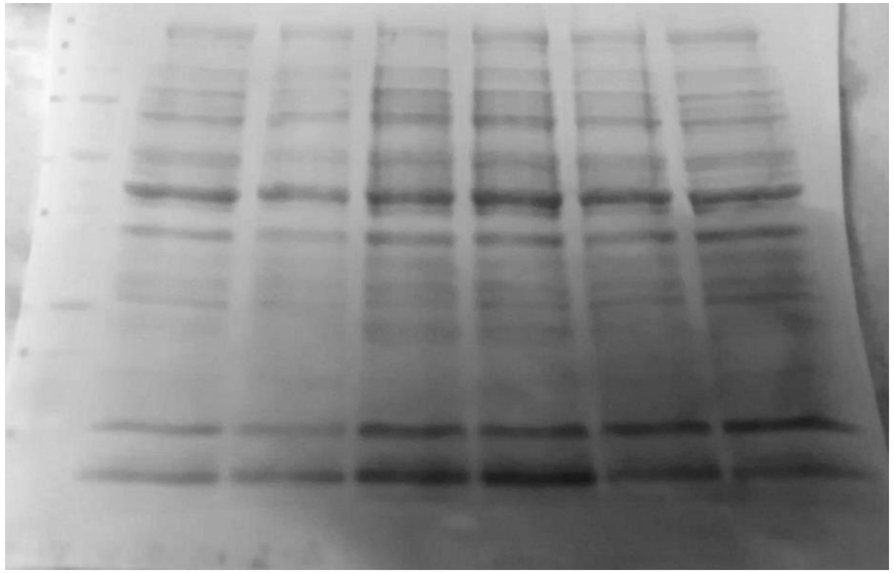

3

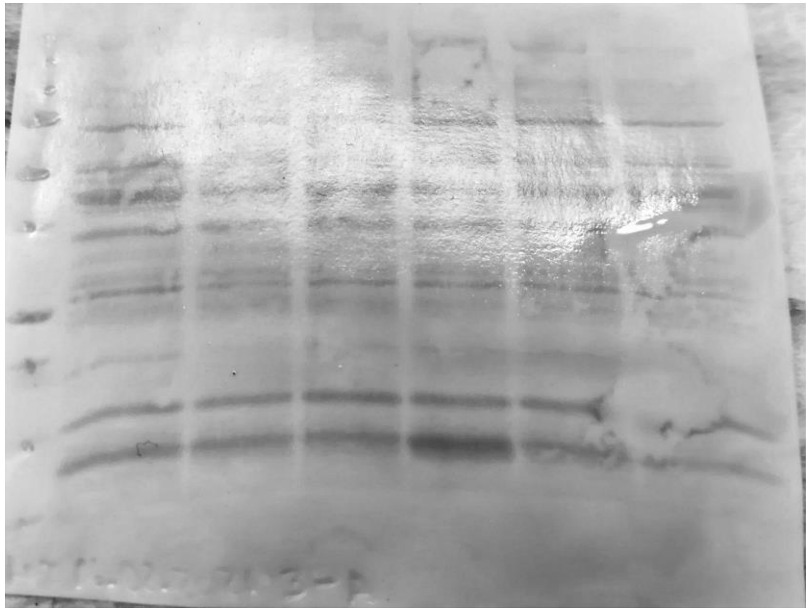

4

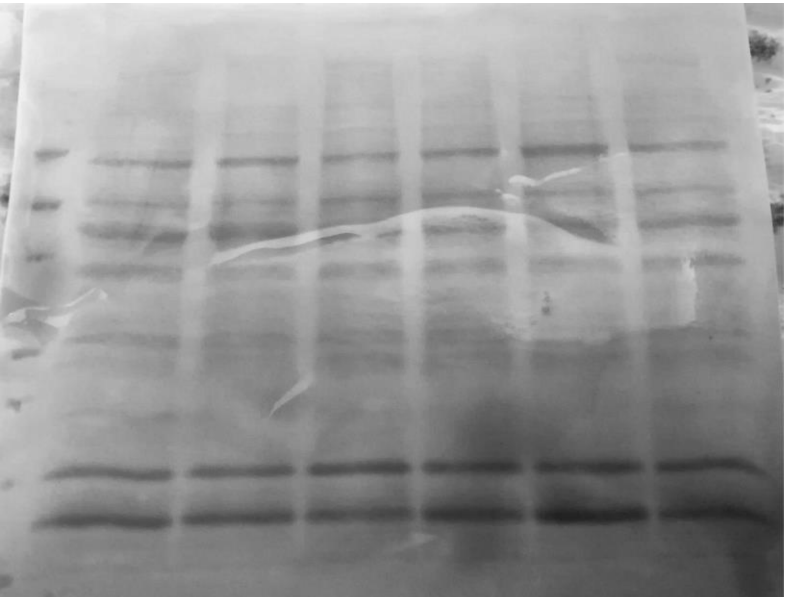

5

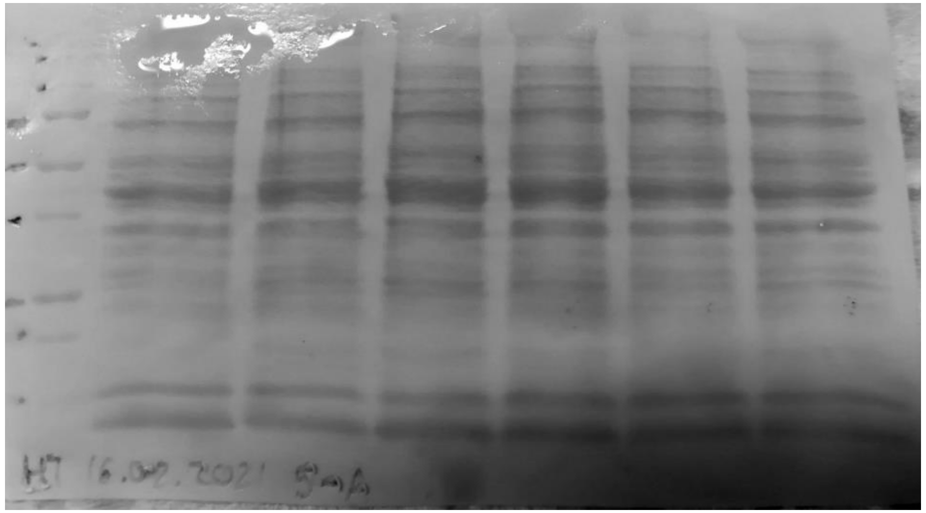

6

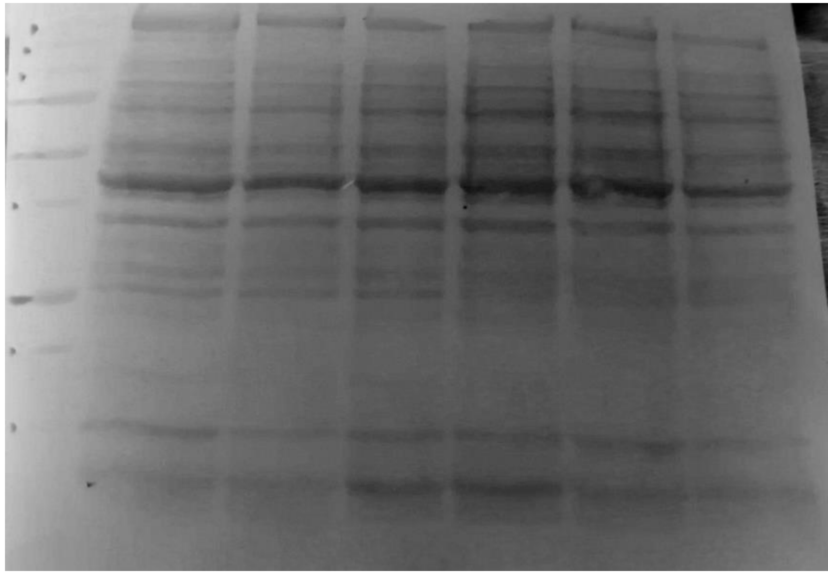

HSP70 - Blot

This study

Samples order from the molecular weight marker: **MTX | DOX18 | CTRL** | DOX9-1 | CTRL-2 | DOX9-2

Other study

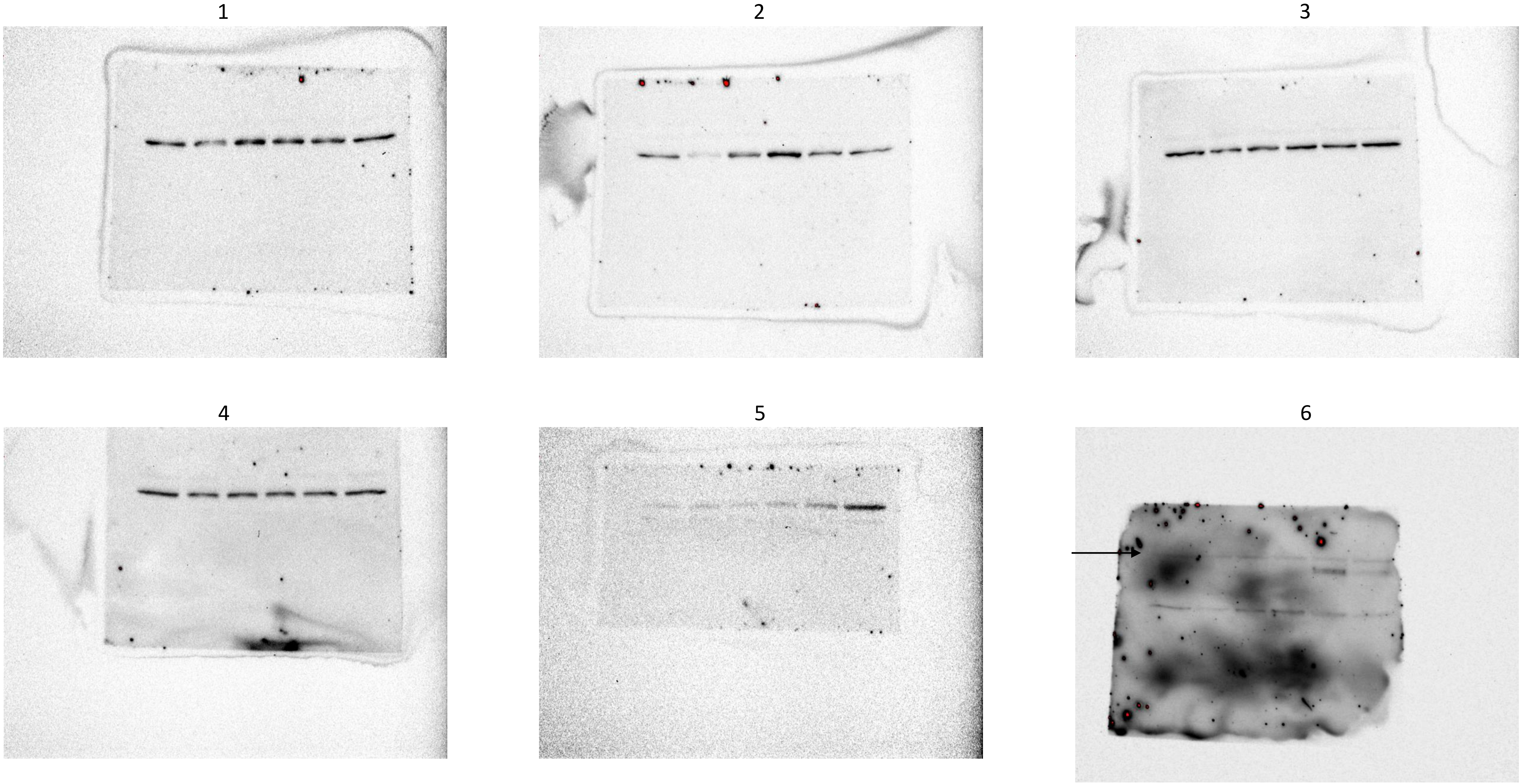

Supplement: Supplementary file 1 [file biomolecules-13-00921-s001.zip › Figure S2.pdf]
