# Supplementary material for: Cardiac Molecular Remodeling by Anticancer Drugs: Doxorubicin Affects More Metabolism While Mitoxantrone Impacts More Autophagy in Adult CD-1 Male Mice
Source: Biomolecules. 2023 May 31;13(6):921. doi: 10.3390/biom13060921 (PMC10296231; doi:10.3390/biom13060921)
Supplement: Supplementary file 1 [file biomolecules-13-00921-s001.zip › SuppData.pdf]

Supplementary data. Histological assessment of liver damage.

Methods:

Histological procedures were done as described [1] for hematoxylin and eosin staining in the liver. The images were taken with a Carl Zeiss Imager A1 light microscope equipped with an AxioCam MRc 5 digital camera (Oberkochen, Germany).

Results:

In the liver, DOX led to vacuolization of the cytoplasm, with signs of microvesicular steatosis, sinusoidal dilation, vascular congestion, as well as interstitial inflammatory cell infiltration, and necrosis (FigureS1). MTX also led to interstitial inflammatory cell infiltration, sinusoidal dilatation, and vascular congestion, but presented necrotic zones more pronounced than DOX. Moreover, MTX caused nuclear pyknosis. Concerning vacuolization, microvesicular steatosis was diffuse and more intense in the periportal region for the MTX group. Thus, overall, MTX seems to have a higher effect on liver structure than DOX.

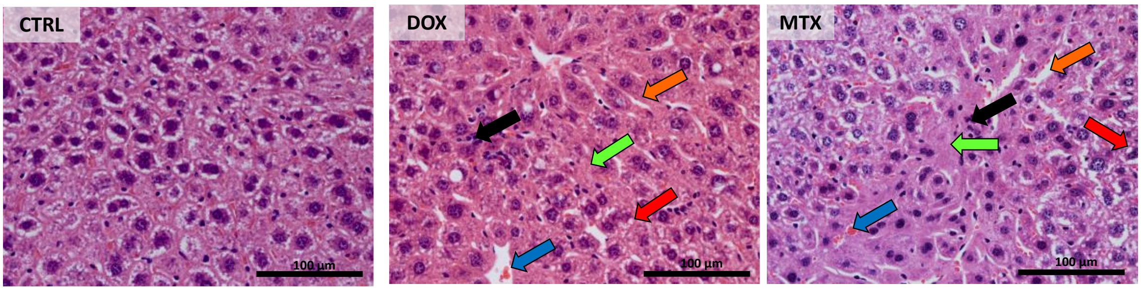

Figure S1. Effect of DOX and MTX on the liver structure assessed by light microscopy. Representative light micrographs obtained from three animals per group were subjected to hematoxylin and eosin staining. The CTRL group showed normal morphology and structure. DOX and MTX groups presented necrotic zones (green arrow), vacuolization (microvesicular steatosis, red arrow), sinusoidal dilation (orange arrow), inflammatory infiltration (black arrow), and vascular congestion (blue arrow). Images were taken at 40× magnification (scale bar = 100 µm).

References:

1. Rossato, L.G.; Costa, V.M.; Dallegrave, E.; Arbo, M.; Silva, R.; Ferreira, R.; Amado, F.; Dinis-Oliveira, R.J.; Duarte, J.A.; de Lourdes Bastos, M.; et al. Mitochondrial Cumulative Damage Induced by Mitoxantrone: Late Onset Cardiac Energetic Impairment. *Cardiovasc Toxicol* **2014**, *14*, 30–40, doi:10.1007/s12012-013-9230-2.
