# Supplementary material for: Cardiac Molecular Remodeling by Anticancer Drugs: Doxorubicin Affects More Metabolism While Mitoxantrone Impacts More Autophagy in Adult CD-1 Male Mice
Source: Biomolecules. 2023 May 31;13(6):921. doi: 10.3390/biom13060921 (PMC10296231; doi:10.3390/biom13060921)
Supplement: Supplementary file 1 [file biomolecules-13-00921-s001.zip › Table S1.pdf]

Supplementary Table S1. Specification of the dilutions and catalog number for each primary antibody.

Primary antibody	Catalog number	Dilution used	Primary antibody	Catalog number	Dilution used
**AMPK**	ab80039	1:1000	**HSP70**	SPA-810	1:1000
**ATG5**	ab108327	1:1000	**LC3B**	L7543	1:1000
**ATPB**	ab14730	1:1000	**Mfn1**	sc-166644	1:1000
**BAX**	ab32503	1:1000	**pAMPK**	ab23875	1:1000
**BCL2**	sc-7382	1:200	**Parkin**	sc-32282	1:1000 or 1:500
**Beclin1**	sc-48341	1:1000	**PFKM**	ab154804	1:1000
**BNIP3**	ab10433	1:1000	**PGC-1α**	ab191838	1:1000
**ETFDH**	ab91508	1:1000	**PPARα**	ab24509	1:1000
**GAPDH**	ab9485	1:1000	**PPARγ**	ab41928	1:1000 or 1:500
**GLUT4**	ab48547	1:1000 or 1:500	**SIRT3**	sc-49744	1:1000
**GSK-3β**	sc-377213	1:500	**TFAM**	sc-28200	1:1000
**HSP27**	sc-9012	1:500			
